# Integrating comparative genomics and risk classification by assessing virulence, antimicrobial resistance, and plasmid spread in microbial communities with gSpreadComp

**DOI:** 10.1093/gigascience/giaf072

**Published:** 2025-06-26

**Authors:** Jonas Coelho Kasmanas, Stefanía Magnúsdóttir, Junya Zhang, Kornelia Smalla, Michael Schloter, Peter F Stadler, André Carlos Ponce de Leon Ferreira de Carvalho, Ulisses Rocha

**Affiliations:** Department of Applied and Environmental Microbiology, Helmholtz Centre for Environmental Research–UFZ, 04318 Leipzig, Germany; Institute of Mathematics and Computer Sciences, University of São Paulo, 13566-590 São Carlos, Brazil; Department of Computer Science and Interdisciplinary Center of Bioinformatics, University of Leipzig, 04107 Leipzig, Germany; Department of Applied and Environmental Microbiology, Helmholtz Centre for Environmental Research–UFZ, 04318 Leipzig, Germany; State Key Laboratory of Regional Environment and Sustainability, Research Center for Eco-Environmental Sciences, Chinese Academy of Sciences, 100085 Beijing, China; Julius Kühn-Institut, Federal Research Centre for Cultivated Plants, Institute for Epidemiology and Pathogen Diagnostics, 38104 Braunschweig, Germany; Helmholtz Center Munich, National Research Center for Environmental Health, Institute for Comparative Microbiome Analysis, 85764 Neuherberg, Germany; Department of Computer Science and Interdisciplinary Center of Bioinformatics, University of Leipzig, 04107 Leipzig, Germany; Institute of Mathematics and Computer Sciences, University of São Paulo, 13566-590 São Carlos, Brazil; Department of Applied and Environmental Microbiology, Helmholtz Centre for Environmental Research–UFZ, 04318 Leipzig, Germany

**Keywords:** risk-ranking, comparative genomics, gene spread, human microbiome, virulence factors, horizontal transmission, metagenome-assembled genomes, antimicrobial resistance

## Abstract

**Background:**

Comparative genomics, genetic spread analysis, and context-aware ranking are crucial in understanding microbial dynamics’ impact on public health. gSpreadComp streamlines the path from *in silico* analysis to hypothesis generation. By integrating comparative genomics, genome annotation, normalization, plasmid-mediated gene transfer, and microbial resistance-virulence risk-ranking into a unified workflow, gSpreadComp facilitates hypothesis generation from complex microbial datasets.

**Findings:**

The gSpreadComp workflow works through 6 modular steps: taxonomy assignment, genome quality estimation, antimicrobial resistance (AMR) gene annotation, plasmid/chromosome classification, virulence factor annotation, and downstream analysis. Our workflow calculates gene spread using normalized weighted average prevalence and ranks potential resistance-virulence risk by integrating microbial resistance, virulence, and plasmid transmissibility data and producing an HTML report. As a use case, we analyzed 3,566 metagenome-assembled genomes recovered from human gut microbiomes across diets. Our findings indicated consistent AMR across diets, with diet-specific resistance patterns, such as increased bacitracin in vegans and tetracycline in omnivores. Notably, ketogenic diets showed a slightly higher resistance-virulence rank, while vegan and vegetarian diets encompassed more plasmid-mediated gene transfer.

**Conclusions:**

The gSpreadComp workflow aims to facilitate hypothesis generation for targeted experimental validations by the identification of concerning resistant hotspots in complex microbial datasets. Our study raises attention to a more thorough study of the critical role of diet in microbial community dynamics and the spread of AMR. This research underscores the importance of integrating genomic data into public health strategies to combat AMR. The gSpreadComp workflow is available at https://github.com/mdsufz/gSpreadComp/.

## Background

The microbial safety of food, water, and environmental matrices has been a critical concern for public health since the 1990s [1]. Different approaches, such as quantitative microbial risk assessment, have provided valuable insights and have been fundamental in evidence-based policymaking in public health. Typically, these approaches involve 4 steps: hazard identification, exposure assessment, dose–response analysis, and risk characterization [2]. However, traditional microbial safety approaches often focus on individual potential pathogens and may overlook community interactions.

Additionally, the advent of high-throughput sequencing technologies has improved our ability to study microbial communities with increased detail. Advances in sequencing technologies can potentially enhance our understanding of microbial ecology and improve microbial analysis’s accuracy, precision, and speed [3]. Concomitantly to the advances in understanding microbial ecology, there is a growing need for community-focused approaches to assess relative impacts across diverse microbial populations. When integrated with exposure and dose–response data, such an approach would equip decision-makers and stakeholders with a more robust risk statement. Specifically, identifying antimicrobial resistance (AMR) spread, virulence factor (VF) spread, and genetic mobility factors are crucial for enhanced microbial risk characterization [3, 4].

Genetic information is spread among entities by vertical gene transfer (VGT) and horizontal gene transfer (HGT). While VGT is relevant for preserving and stabilizing genetic material, HGT has a crucial role in the evolutionary and adaptive process [5]. Consequently, HGT allows microbes in microbial communities to perform functional leaps and rapidly adapt to new environments. There are 3 most recognized mechanisms of HGT in prokaryotes: conjugation, transformation, and transduction. Conjugation requires physical contact between the cells. Transformation is the uptake of exogenous DNA, mostly plasmids, from the environment. Transduction is the delivery of genetic material through viruses and virus-like agents [6]. However, even though transduction and transformation events are effective for gene exchange, plasmid-mediated conjugation is often recognized as the most impactful HGT mechanism [7]. Plasmids often carry genes that allow potential selective advantages (e.g., AMR or heavy metal resistance, VFs, and degradation of xenobiotics) [8, 9].

Specifically, the spread of AMR in clinical and natural environments is recognized as one of the most significant global threats [10, 11]. The misuse of antibiotics in agriculture, the environment, and human medicine creates selective pressure on antimicrobial-resistant bacteria (ARB), which may facilitate the HGT of those resistances. Antibiotics are extensively used for farm animal and plant production [12, 13]. In 2015, a notable trend emerged in the United States, where 62% of antibiotics initially intended for use in food-producing animals were ultimately used in human medicine. Additionally, 70% of medically relevant antibiotics were sold for animal use [14]. Furthermore, while the use of antibiotics in plant agriculture is generally considered lower than in human and veterinary medicine, recent studies suggest it may be more widespread than previously thought. Streptomycin, oxytetracycline, kasugamycin, oxolinic acid, and gentamicin are commonly used in crop protection, particularly in the American and Asian continents [15].

In addition, HGT events provide rapid adaptation to bacteria strains, including AMR, making the development of novel antimicrobials only a short-term palliative measure [16]. Minimizing problematic HGT and disseminating antimicrobial resistance genes (ARGs) is the potential long-term solution to the AMR problem. Inherently, advances in understanding plasmid-mediated HGT dynamics in complex microbiomes are a powerful tool to control horizontal dissemination [17, 18].

Although HGT events, specifically plasmid-mediated transfers, play a significant role in the evolution and adaptation of microbial populations, most of those events remain undetected. Consequently, several bioinformatics tools and algorithms were developed to tackle HGT events. For instance, GIST [19] and IslandViewer [20] use genome sequences’ features to assign HGT. DarkHorse [21] and HGTector [22] use the “best matches” approach to identify HGT events based on reference genomes. Other methods, such as Ranger-DTL [23] and AnGST [24], require the reconciliation of gene trees with the corresponding species trees to make the HGT prediction. Finally, the MetaCHIP [25] tool combines the results of the similarity and phylogenetic approaches.

A significant limitation of most current HGT detection methods is that they are not directly applicable to the entire microbiome but more for single bacteria taxa. In addition, most methods require reference genomes. For instance, the HGTector [22] is restricted to HGT events from a defined distal group to designated self-group members, while DarkHorse [21] requires a reference genome, a bottleneck for uncultured microorganisms. MetaCHIP [25] can be applied at the community level, given a set of recovered genomes. However, MetaCHIP [25] does not directly integrate its results into relevant sample metadata (i.e., biome, clinical data, environmental condition), reducing its usage for comparative genomics. In addition, none of the mentioned tools allows for direct integration of plasmid-mediated transfer of annotated genes to potential pathogenic bacteria by using, for example, comparative genomics, which creates a significant barrier for non-bioinformaticians, mainly clinicians, to use such datasets. Finally, plasmids have also been reported to be transferred over considerable taxonomic distances, adding complexity for HGT detection tools to identify plasmid-mediated transfer in complex microbial communities [25, 26].

**Figure 1:**
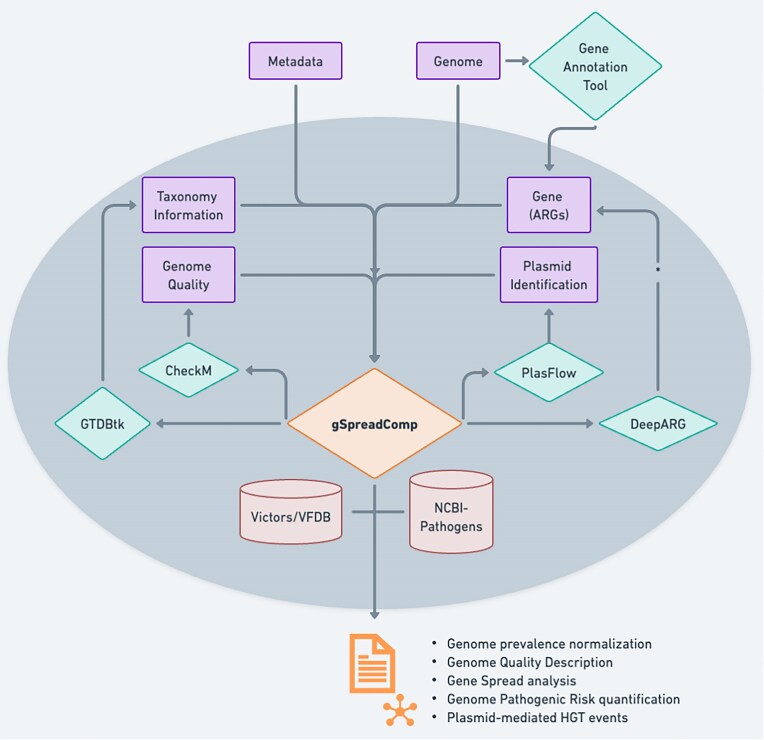
gSpreadComp workflow. The minimal input necessary for gSpreadComp is the genome and its associated metadata. gSpreadComp offers the possibility to use the built-in prokaryotic taxonomy assignment using GTDBtk, prokaryotic quality estimation using CheckM, plasmid identification using PlasFlow, and ARG annotation using DeepARG. Alternatively, any other tool could be used outside gSpreadComp and later used as input to estimate gene spread, microbial resistance-virulence risk, and gene plasmid-mediated HGT events. The gSpreadComp can use the Victors or the VFDB to annotate virulence potential on target genomes and the NCBI human Pathogens Species database as a reference to estimate potential pathogens.

We designed the gSpreadComp workflow to tackle the following bottlenecks: (i) reduce the barrier of comparative genomics by integrating genome annotation, normalization, and sequence comparison into a unified approach; (ii) create a systematic approach to quantify gene spread; (iii) integrate plasmid-mediated gene transfer annotation to target metadata with the whole-microbiome community in a genome-reference independent approach; and (iv) provide a resistance-virulence risk-ranking metric that considers gene spread, prokaryotic resistance potential, and virulence potential in the era of high-throughput microbial community sequencing. Consequently, gSpreadComp is a UNIX-based workflow for genome analysis (Fig. 1) that provides 6 modules to perform the following tasks: taxonomy assignment, genome quality estimation, ARG annotation, plasmid/chromosome classification, VF annotation, and in-depth downstream analysis.

To demonstrate the potential of the gSpreadComp workflow, we analyzed the spread of ARGs in the human gut microbiome from human subjects with different diets. To this end, we gathered publicly available metagenomes from the human gut containing information about the subjects’ diet: (i) ancient, diet based on the analysis of ancient human fecal remains; (ii) ketogenic, fecal samples from subjects with a high-fat, high-protein, low-carbohydrate diet; (iii) omnivore, fecal samples from subjects with a diverse diet, including both plant- and animal-derived foods; (iv) vegan, fecal samples from subjects with a plant-based diet, excluding all animal-derived products; and (v) vegetarian, fecal samples from subjects with diet excluding meat but may include other animal-derived products. We then recovered the metagenome-assembled genomes (MAGs) from those samples and annotated their ARGs and taxonomy. Finally, those MAGs were analyzed using gSpreadComp using the subjects’ diet as the target metadata. Notably, the primary objective of this use case is not to draw definitive conclusions about the relationship between diet and antimicrobial resistance or virulence but to exemplify how gSpreadComp can be applied to complex metagenomic datasets.

Our data revealed antimicrobial resistance, particularly to multidrug and glycopeptide classes, to be widespread across all diets, with specific resistances like bacitracin being more prevalent in vegans. Additionally, while all diets exhibited similar overall resistance spread, nuances like increased tetracycline resistance in omnivores were observed. The study also highlighted a complex relationship between diet and VFs, with specific diets showing heightened resistance-virulence risks, like ketogenic. Finally, vegans and vegetarians were associated with a higher potential to participate in plasmid-mediated HGT events, underscoring the significant role of diet in shaping microbial communities and antimicrobial resistance patterns. While further laboratory validation is required, gSpreadComp accelerates the identification of potential targets, streamlining the path from *in silico* analysis to hypothesis validation through experimental verification.

## Findings

### The gSpreadComp workflow

The gSpreadComp workflow is a UNIX-based integrated set of tools for genome analysis (Fig. 1). For such, it provides 6 modules to perform the following tasks: taxonomy assignment, genome quality estimation, ARG annotation, plasmid/chromosome classification, VF annotation, and in-depth downstream analysis. This downstream analysis includes target-based gene spread analysis, plasmid-mediated HGT of target genes and VFs, and a prokaryotic resistance-virulence risk-ranking within the analyzed genomes. It is important to note that gSpreadComp is essentially modular, allowing for the integration of new advances in its component methods and tools as they become available.

The spread of target genes was calculated using the genes’ weighted average prevalence (WAP), which estimates the gene spread at different taxonomical levels or target groups (e.g., omnivores, vegans, ketogenic). More details can be found in the Methods section. For resistance-virulence risk-ranking, we defined the “resistance-virulence potential factors” that consider target genes (ARGs, by default), virulence, and their plasmid transmissibility potential. Reference potential pathogens were identified by comparing genomes to the NCBI pathogens database [27]. Following, we used the average of the resistance-virulence factors from the reference potential pathogens, based on the NCBI Pathogens Organism groups, as weights and quantified the resistance-virulence risk using the Technique for Order Preference by Similarity to Ideal Solution (TOPSIS) [28], with the resistance-virulence factors serving as input vectors. After the complete downstream analysis, gSpreadComp produced an HTML report.

The gSpreadComp workflow includes an easy-to-use script that downloads and configures the required databases automatically. Consequently, if the user is interested in ARG spread, the only mandatory inputs for gSpreadComp are the genomes and their target metadata. Suppose the user is interested in a different target gene group. In that case, they should provide the annotation table formatted as described in the gSpreadComp documentation. A database update is scheduled to happen every January and July.

Part of gSpreadComp is a wrapper of several bioinformatic approaches. Its modular nature makes it possible to use the tools independently, allowing the use of the tools’ main analysis and the related report without the need to annotate it within the software completely. Additionally, the modular nature of the software facilitates its update and allows the more experienced user to integrate only pieces of gSpreadComp into their pipeline. Consequently, gSpreadComp modularity can give the researcher flexibility in their analysis and facilitate the investigator’s software management necessities. The gSpreadComp workflow was designed to support Linux x64 systems. The complete software installation requires approximately 15 GB. The whole database currently requires around 92 GB.

### Critical usage and key considerations

Before presenting the experimental results, it is crucial to address specific methodological considerations and limitations in the methods. The gSpreadComp workflow can be used with both complete genomes and MAGs. In our use case, we applied gSpreadComp to MAGs, which are prone to higher potential bias [29]; for example, MAGs are subject to detection bias, particularly for low-abundance organisms, which may lead to the underrepresentation of certain species and their associated ARGs. Additionally, even high-quality MAGs (completeness >90% and contamination <5%) may be exposed to contig binning error, causing contamination [30]. Finally, there are sample size effects. To mitigate the impact of sample size, gSpreadComp employs normalization techniques and weighted average prevalence for spread calculations [31]. Nevertheless, users should note that the resulting resistance-virulence risk-ranking is relative to the analyzed community and not an absolute measure across environments.

The ARG annotation module provided within gSpreadComp uses a machine learning–based classification tool named DeepARG [32]. While DeepARG has demonstrated high accuracy in ARG prediction, its performance can vary according to the antibiotic category and its representation in the training database. For long sequences (DeepARG-LS), the tool achieved precision and recall values equal to 0.99 in the prediction of different categories of ARGs. To minimize false positives, we followed benchmarked recommendations, including using a minimum 80% prediction probability, an e-value alignment lower than 1e-10, and a percent identity of 35% or higher [33]. It is important to note that the user can alter the hyperparameters (e.g., prediction probability, e-value alignment). Users should interpret results with these constraints in mind. Similarly, for plasmid detection, we currently use PlasFlow [34]. While effective, PlasFlow has limitations in classifying shorter sequences. We increased the classification threshold parameter (0.7 > threshold) in our analysis to improve precision while maintaining the high sensitivity, or recall, offered by PlasFlow’s models [34, 35]. However, it must be observed that automatically classifying plasmids remains complex, with significant advances currently in development. Those approaches were selected because of their ability to streamline large-scale annotation and detection while having higher recall, which is particularly important when dealing with MAGs.

The gSpreadComp workflow was designed to be modular and extendable, allowing a more straightforward incorporation of additional features in its future versions as the field rapidly evolves. For instance, ARG detection tools like ARG-SHINE [36] or CARD-RGI [37] or plasmid classification tools like PlasClass [35] or PLASMe [38] can be used, and their results are integrated into gSpreadComp downstream analysis, provided that the users format their data according to the gSpreadComp documentation. We encourage users to consider the strengths and limitations of each tool when interpreting results and to validate findings through complementary experimental approaches when possible. It is important to note that gSpreadComp’s downstream results rely on the tools’ annotations, and results for simulated communities would closely follow their benchmarked performance.

#### Use case: gSpreadComp in the human gut microbiome of subjects with different diets

To show the potential of gSpreadComp to generate hypotheses, we analyzed the spread of ARGs and virulence factors in the human gut microbiome from subjects with different diets. It is important to mention that the primary objective of this use case is not to draw definitive conclusions about the relationship between diet and antimicrobial resistance or virulence but to illustrate how gSpreadComp can be applied to complex metagenomic datasets to generate insights that could inform more comprehensive risk assessments.

We recovered MAGs of 17 ketogenic, 10 vegan, 40 vegetarian, and 140 omnivore subjects from the human gut. In addition, we recovered MAGs from 24 palaeofeces samples dating from 1,300 and 5,300 years old (Additional File 1: Supplementary Table S1). We recovered 3,566 MAGs (1,806 high and 1,760 medium quality) from 231 samples (Additional File 2: Supplementary Table S2). The taxonomic assignment indicated that the MAGs came from 637 species of 12 phyla (Additional File 2: Supplementary Table S2a). According to GTDB-tk, 594 recovered species were assigned to previously recovered genomes, and 43 species groups found are potentially new.

Our analysis included ancient DNA samples, which present unique challenges. Ancient DNA is typically degraded and fragmented, potentially affecting gene annotation accuracy. Moreover, these samples are highly susceptible to contamination from modern sources and postmortem microbial colonization. For instance, DNA degradation and potential contamination may lead to a skewed number of false negatives detected due to incomplete gene sequences or false positives due to modern contamination [39]. While we have taken steps to address these issues, distinguishing endogenous ancient DNA from contaminants remains challenging. These factors do not invalidate our findings but underscore the need for cautious interpretation, especially when comparing ancient and modern microbiomes [39].

**Figure 2:**
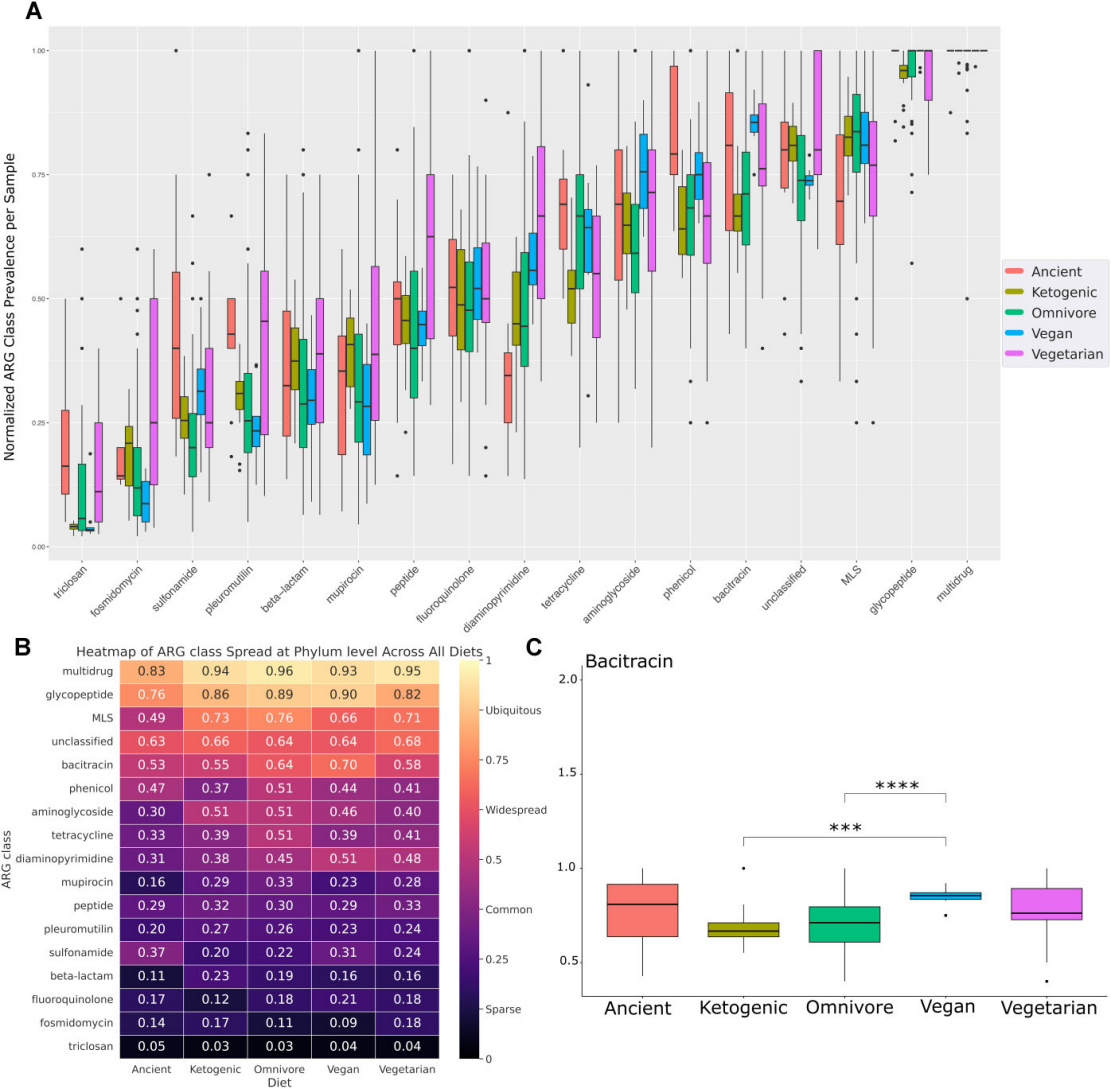
gSpreadComp estimated target gene spread in given metadata. (A) Boxplot from normalized ARG class prevalence per sample colored by diet. The ARG classes are sorted left to right in ascending order according to average ARG class prevalence. (B) Heatmap colored by WAP, used to estimate the spread at the phylum level across all analyzed diets. Values from 0 to 0.25 are considered sparse, 0.25 to 0.5 common, 0.5 to 0.75 widespread, and 0.75 to 1 ubiquitous. (C) Boxplot from normalized bacitracin prevalence per sample colored by diet. A pairwise comparison between the diets was made using the Bonferroni-adjusted *t*-test. Statistically significant comparisons (adjusted *P* < 0.05) are indicated by *. The higher the number of *, the closer to 0 the adjusted *P*-value.

We annotated 356 ARG subtypes distributed in 24 different ARG classes (Additional File 3: Supplementary Table S3a). In the ancient samples, we annotated 211 unique ARGs belonging to 22 unique ARG classes. In contrast, ketogenic had 234 and 18, omnivores had 320 and 22, vegans had 238 and 21, and vegetarians had 246 and 20, respectively, in their gut microbiome. We also normalized ARG class prevalence per sample (Additional File 3: Supplementary Table S3b). We kept only the samples that recovered more than 6 genomes for further prevalence analysis. Figure 2A shows the normalized prevalence of the ARG classes per sample for all eating habits. In addition, we performed pairwise ARG class prevalence comparisons for all diets (Additional File 3: Supplementary Table S3c and Additional File 4: Supplementary Fig. S1). The bacitracin resistance boxplot comparisons can be found in Fig. 2C.

Further, we estimated the ARG class spread at the phylum level in gut samples of subjects across the different diets (Additional File 5: Supplementary Table S4a). We defined the following ranges to describe the distribution of ARG classes: sparse (0–0.25), common (0.25–0.5), widespread (0.5–0.75), and ubiquitous (0.75–1). A heatmap with the distribution at the phylum level value per ARG class for all diets can be found in Fig. 2B. Multidrug and glycopeptide resistance were ubiquitous in all subjects, irrespective of the diet. For further analysis, we excluded ARG classes exhibiting a distribution of less than 0.1 across all dietary patterns. The results revealed that among the diets, omnivores exhibited the highest spread in six ARG classes: multidrug, MLS (macrolides, lincosamides, streptogramins), phenicol, aminoglycoside, tetracycline, and mupirocin. In contrast, vegans demonstrated the highest spread in 4 ARG classes: glycopeptide, bacitracin, diaminopyrimidine, and fluoroquinolone. For the remaining dietary patterns, the ketogenic diet had the highest spread in 2 ARG classes (pleuromutilin and beta-lactam), the vegetarian diet in 2 (peptide and fosmidomycin), and the ancient subjects in 1 (sulfonamide). However, considering only the ARG classes with at least a 5% difference between all other diets, bacitracin is more spread in vegans, tetracycline in omnivores, and sulfonamide in the ancient diet. When we compared ketogenic and omnivore (meat eaters) against vegans and vegetarians (not meat eaters) according to the mean spread value, we observed that meat eaters had a higher spread for MLS, aminoglycoside, and mupirocin, and non–meat eaters for diaminopyrimidine.

**Figure 3:**
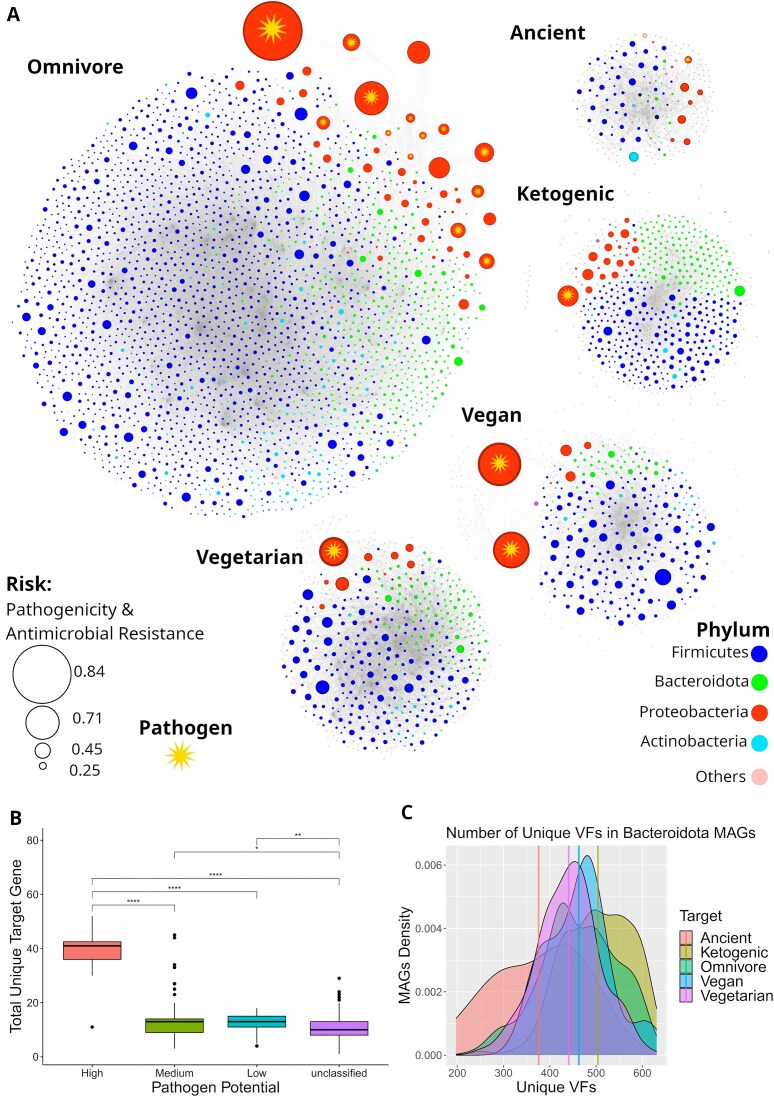
gSpreadComp estimates the resistance-virulence risk from MAGs. (A) Network representation from the recovered MAGs (nodes) distributed according to the co-occurrence of ARGs for the 5 different diets. The node size represents the resistance-virulence risk of a MAG. The node color represents the phylum. As expected, the potential pathogens (identified based on the NCBI Pathogen detection database), marked with a star, systematically have a high risk, but in the ancient diet. The highest resistance-virulence MAG was found in the omnivore diet, followed by *Proteobacteria* MAGs from vegans. Interestingly, the number of ARGs in plasmids is the most significant metric to calculate the risk, followed by VFs in plasmids. This result indicates that a higher resistance-virulence risk is associated with the presence of the observed genes in mobile elements. This may be intuitive, as those MAGs are more likely to participate in plasmid-mediated horizontal transmission and contribute to a resistant microbiome. (B) Boxplot from MAGs grouped by pathogen potential on the x-axis and the number of unique ARGs annotated in the MAG on the y-axis. A “high” pathogen potential indicates that the MAG is from a species present in the NCBI Pathogen Detection Database, and “medium” and “low” indicate a MAG from the same genus and family, respectively. The boxplot indicates high antimicrobial resistance from high potential pathogens compared with the other MAGs. (C) The density of MAGs from the *Bacteroidota* phylum, based on the total number of annotated unique VFs. The density plot shows a significant negative skew for the ketogenic diet, while the ancient diet has a positive skewness, and the other diets tend to have a normal distribution. This indicates that the ketogenic diet may potentially increase the resistance-virulence risk from *Bacteroidota*.

Finally, gSpreadComp also allowed us to individually compare the spread of ARGs among phyla (Additional File 5: Supplementary Table S4b–f and Additional File 6: Supplementary Fig. S2). The results are summarized in Table 1. The subsequent results that gSpreadComp provided were the annotation of VF (Additional File 7: Supplementary Table S5a). The average numbers of unique VFs annotated per diet were 479.75 ± 116.41 for ancient, 444.56 ± 88.03 for ketogenic, 444.54 ± 106.24 for omnivore, 475.86 ± 163.95 for vegan, and 438.13 ± 108.40 for vegetarian. We also verified the average number of unique VFs per phylum per diet (Additional File 7: Supplementary Table S5b). Specifically, *Bacteroidota* related to the ketogenic diet had statistically more unique VFs than all the other diets (Fig. 3C and Additional File 7: Supplementary Table S5c). Additionally, gSpreadComp calculated all the statistical significance comparisons associated with the unique number of VFs (Additional File 7: Supplementary Table S5c). We verified, as expected, that MAGs with high pathogenic potential, irrespective of the diet, have a higher number of unique VFs in the gut samples (Additional File 2: Supplementary Table S2a and Additional File 8: Supplementary Fig. S3a). More interestingly, we observed that, irrespective of the diet, highly virulent bacteria had statistically more ARGs in the respective gut samples (Fig. 3B and Additional File 7: Supplementary Table S5d).

**Table 1:** ARG class spread summary for the common phyla across the different diets. The values represent ARG classes with a spread difference greater than 0.05 in the respective diet for the respective phylum compared to other diets. While measures were taken to reduce false positives, some errors may still be present, particularly for ARGs underrepresented in databases (e.g., triclosan). Caution is advised when interpreting results from ancient samples due to potential DNA degradation and contamination issues. It is important to note that despite the 0.05 difference threshold used here, most ARG classes fell into the same spread category (e.g., sparse, common, widespread, or ubiquitous) across all diets, indicating a general consistency in ARG distribution patterns.

Diet	Phylum		
	Bacteroidota	Firmicutes	Proteobacteria
Omnivore	MLS, beta-lactam, fluoroquinolone, multidrug, mupirocin	MLS, aminoglycoside, mupirocin, tetracycline	Diaminopyrimidine
Vegan	Aminoglycoside, diaminopyrimidine, phenicol, pleuromutilin	Bacitracin, diaminopyrimidine	Aminoglycoside, bacitracin, fluoroquinolone, pleuromutilin, tetracycline
Ketogenic	Bacitracin, glycopeptide, peptide	—	—
Vegetarian	Fosmidomycin, tetracycline	Fluoroquinolone	Mupirocin, phenicol
Ancient	Sulfonamide	Phenicol, sulfonamide	MLS, beta-lactam, fosmidomycin, glycopeptide, multidrug, peptide, sulfonamide, triclosan

Finally, we rank the potential resistance-virulence risk for all recovered MAGs (Additional File 2: Supplementary Table S2a). Figure 3A shows a graph where the nodes are sized according to the risk criteria. For the risk criteria, we highlight the results found for the *Firmicutes* phylum, where statistically significant differences were found between omnivores vs. vegetarians and vegans, as well as between ketogenic vs. vegetarians and vegans, with an increased rank observed for the vegetarian and vegan MAGs. However, there was no difference between omnivores and ketogenic, or between vegans and vegetarians (Additional File 7: Supplementary Table S5e and Additional File 8: Supplementary Fig. S3b, c). Finally, gSpreadComp compiled all potential plasmid-mediated HGTs for the target gene (ARGs, in this use case) and the VFs at a defined taxonomical level (Additional File 9: Supplementary Table S6a for ARG HGT events and Additional File 9: Supplementary Table S6b for VF HGT events). We removed the libraries that recovered fewer than 12 MAGs before the HGT analysis to reduce comparison bias due to limited MAG reconstruction. After filtering, all diets had an average of 26 MAGs per sample. However, vegans and vegetarians had 12 ARG plasmid-mediated HGTs per sample, while omnivores had 3.88 and ketogenic 1.84 (Additional File 9: Supplementary Table S6c). We observed a significant increase in the ARGs and VFs involved in potential plasmid-mediated HGT in the vegans and vegetarians compared to ancient, omnivore, and ketogenic. Then, we performed pairwise Bonferroni statistical comparisons related to the HGT events between the diets (Additional File 9: Supplementary Table S6c–e and Additional File 8: Supplementary Fig. S3b–e). All pairwise comparisons against vegans or vegetarians were significant (adjusted *P* < 0.05), but there was no significant difference among any other comparison, or between vegans and vegetarians. Similarly, vegans and vegetarians had significantly more VFs plasmid-mediated HGT events per sample (Additional File 9: Supplementary Table S6d, e). Additionally, gSpreadComp allowed for the calculation of the pairwise comparisons related to the occurrence of HGT events per defined taxonomical level (family) per diet (Additional File 9: Supplementary Table S6f–h). We identified HGT events of VFs, and a significant difference was observed for the cases in Table 2. In the HGT events of ARGs, a significant difference was only accessed for *Ruminococcaceae* in omnivores and vegans and *Lachnospiraceae* in vegetarians and ketogenic.

**Table 2:** Pairwise comparison of the number of plasmid-mediated HGT events involving VFs in which specific bacterial families participated. The comparison is made between samples from individuals following different diets. The columns represent the 2 diets being compared, the adjusted *P*-value for statistical significance, and the bacterial family involved.

Diet 1	Diet 2	Adjusted *P*-value^a^	Family
Omnivore	Vegetarian	0.0014	*Lachnospiraceae*
Omnivore	Vegan	0.0030	*Lachnospiraceae*
Omnivore	Vegan	0.0032	*Ruminococcaceae*
Vegetarian	Ketogenic	0.0051	*Lachnospiraceae*
Omnivore	Vegetarian	0.0136	*Oscillospiraceae*
Vegan	Ketogenic	0.0142	*Ruminococcaceae*
Vegetarian	Ketogenic	0.0204	*Oscillospiraceae*
Vegan	Ketogenic	0.0433	*Lachnospiraceae*
Omnivore	Vegetarian	0.0439	*Ruminococcaceae*

a Bonferroni adjusted *t*-test.

## Discussion

### The gSpreadComp

gSpreadComp was designed for 2 main goals: (i) to facilitate comparative genomics and (ii) to integrate high-throughput sequencing information into microbiome relative resistance-virulence risk-ranking, with a focus on the potential presence of antimicrobial resistance genes and virulence factors.

At its core, gSpreadComp integrates genome annotation, gene prevalence normalization, and sequence comparison into a streamlined approach, thereby reducing the complexities often associated with disparate tools. Furthermore, the tool introduced a systematic methodology to quantify gene spread, a crucial aspect in understanding gene dispersion populations.

Second, gSpreadComp effectively uses whole-genome sequencing (WGS) data by providing a standardized method to rank potential microbial communities of concern using metagenomic samples. Highlighting hotspots of resistance and virulence factors narrows the focus for subsequent hypothesis testing through laboratory-based assessments. While not performing risk assessments directly, gSpreadComp may guide more targeted and efficient laboratory studies, ultimately improving resource allocation and preventive measures. Finally, tracking plasmid-mediated HGT can contribute insights into antimicrobial resistance, or any target gene, transfer routes that remain largely uncharted. gSpreadComp also contributes to identifying key disseminating taxa and potential propagation pathways. Such knowledge is vital for developing strategies to combat the rise of antimicrobial-resistant pathogens and constructing more comprehensive microbial risk assessment models [40].

While gSpreadComp’s main strengths lie in its downstream analysis and unified workflow, it has limitations and biases that should be considered when interpreting results. These may stem from genome recovery techniques, reference databases, or machine learning algorithms used in the tool. As with any bioinformatic approach, we recommend a critical usage.

### Critical usage and key considerations

While not a standalone risk assessment tool, gSpreadComp provides a framework for comparing the relative rank associated with resistance and virulence genes across microbial populations. When used with established microbial risk assessment guidelines, gSpreadComp can enhance the depth and precision of risk-rank evaluations. By integrating genomic data analysis with traditional risk assessment approaches, researchers may gain more comprehensive insights into potential microbial hazards, thereby supporting more informed decision-making in public health, environmental management, and food production contexts [1].

In particular, it is relevant to notice the distinction between relative resistance-virulence risk-ranking, which gSpreadComp provides, and risk assessment. While our tool offers insights into the comparative potential resistance-virulence risks within microbial populations based on their genomic profiles, it does not account for all factors considered in a full risk assessment, such as exposure routes, dose–response relationships, and specific environmental conditions [3]. Users should view gSpreadComp’s output as a starting point for prioritizing further investigation.

When considering ARG annotation using machine learning algorithms, one must know that ARG prediction accuracy varies per gene and class based on the representation and degree of similarity to known resistance genes in the training databases. For sequences with high identity scores (>50%) to the training data, both alignment-based methods, such as BLAST, and classification-based approaches, such as DeepARG or ARG-SHINE, perform well, with around 95% accuracy [36]. However, classification models tend to perform better for sequences with low identity scores. For instance, sequences conferring resistance to bacitracin, beta-lactams, and MLS are more represented in the databases and more accurately predicted by DeepARG than resistances such as triclosan or quinolone. The more drastic improvement of classification-based methods is in reducing false-negative rates while maintaining overall high precision. For long ARG-like sequences, DeepARG-LS achieved 0.97± 0.03 precision and 0.99 ± 0.01 recall for bacitracin, beta-lactamase, chloramphenicol, and aminoglycoside, while the best-hit approach achieved perfect precision but 0.48 ± 0.2 recall [32]. This significant difference in recall is particularly crucial when annotating MAGs, which are often fragmented. Importantly, the presence of an ARG does not necessarily equate to phenotypic resistance but also depends on gene expression and host factors and potential bias in the resistance genotype–phenotype concordance on less characterized taxa [41].

Generally, using machine learning–based methods for the classification of biological sequences, while promising, has challenges and limitations. Classifying plasmids can be particularly challenging since they usually exhibit high genetic diversity [38] and shared sequence segments between plasmids and chromosomes. Tools like PlasFlow and PlasClass provide a promising alternative for detecting more diverged plasmids via learning patterns beyond sequence similarity but tend to have decreased precision. On the other hand, hybrid methods, like PLASMe, tend to be computationally more costly. Consequently, users should be aware of these methodological differences when interpreting results and consider the strengths and limitations of each approach in the context of their specific research questions. For gSpreadComp, as an auxiliary tool for hypothesis generation, we decided to initially deploy it with the machine learning–based method PlasFlow for its comparative results with PlasClass, but with slightly higher recall [35]. However, as the plasmid detection tools rapidly evolve, we expect to update the gSpreadComp plasmid detection module in the future.

Similarly, machine learning–based methods have been used for VF annotation [42–44]. However, to the best of our knowledge, less work has been done on the reliability of those tools when applied to MAGs, specifically when looking for individual VF. Therefore, for VF annotation, we implemented a best hit–based method in gSpreadComp, potentially increasing the number of false negatives for the sake of precision.

#### Use case: gSpreadComp in the human gut microbiome of subjects with different diets

Previous studies have suggested potential links between diet and antibiotic resistance patterns, with some focusing on meat consumption [45–47]. Simultaneously, growing evidence shows that uncooked produce could contribute to higher HGT events and potential antibiotic resistance spread [48–51]. While these findings provide interesting hypotheses, our use of gSpreadComp aims to demonstrate a streamlined approach for analyzing resistance gene spread across diverse groups and draw attention to potential resistance-virulence transmissibility hotspots rather than to draw definitive conclusions about diet–resistance relationships.

### Antimicrobial resistance spread

We identified multidrug and glycopeptide resistance genes as ubiquitous in fecal samples from subjects of every diet, including ancient. Glycopeptide antibiotics have been mainly used to treat multidrug-resistant Gram-positive infections, and increased resistance occurrence has already become a cause of concern [52]. Specifically, its overuse in the livestock industry was pointed out almost 20 years ago [53]. Glycopeptide resistance genes were, however, also found in permafrost from >10,000 years ago [54]. In addition, an extensive metagenomic study of soil, ocean, and animal sources found that glycopeptide resistance–related genes were prevalent in all samples, accounting for 17% of global resistant sequences, second only to multidrug resistance efflux pumps [55].

When analyzing resistance with at least a 0.05 increase in the spread in one particular diet, we observed a specific increase in bacitracin resistance for vegans (0.7–widespread), followed by omnivores (0.64–widespread), and then the subjects from the other 3 diets (0.55 on average). Interestingly, bacitracin is not typically used orally but instead applied topically in ointments [56]. In addition, bacitracin has been extensively used as an animal feed additive [57]. Although still under the “low” widespread category previously established, tetracycline resistance genes were more disseminated in omnivores, 0.51, while subjects preferring the other diets had a similar spread of 0.40, considered “common.” Tetracycline is typically used for therapeutic purposes but is reportedly frequently added to livestock feed at doses below therapeutic levels, and it has been used as a growth enhancer for swine, poultry, and aquaculture mainly in the past century [58].

When we grouped the subjects with diets exposed to animal meat (ketogenic and omnivore) against the nonexposed (vegans and vegetarians), we saw an increase in spread for the MLS, aminoglycoside, and mupirocin resistance. It is relevant to notice that MLS was considered ubiquitous-widespread and aminoglycoside widespread-common in all diets. MLS has been used in European cattle and pig husbandry [59]. Similarly, a 2023 study has explored aminoglycoside detection in several animal muscles, tissues, honey, milk, and other food sources. They were able to detect the antibiotic in 17% of the samples. Most of these samples were retrieved from cattle and swine [60]. The mupirocin resistance was less spread than the others mentioned. We considered mupirocin in the sparse-common range for all diets.

In our investigation of ARG classes, we observed an elevated spread of diaminopyrimidines that exhibited a more pronounced distribution among vegetarians and vegans, closely followed by omnivores and a lower spread in the ketogenic diet group. A recent study found ubiquitously accumulating diaminopyrimidines, fluoroquinolones, and sulfonamides in rice farms [61]. The study found a higher accumulation of fluoroquinolones and sulfonamide. Consistent with our results, the ancient subjects exhibited the highest prevalence of sulfonamide, 0.37, followed by vegans, 0.31, and vegetarians, 0.24.

It is worth noticing that although there are specific differences in resistance spread, all modern diets showed a similar overall spread distribution. On the other hand, by calculating the average ARG class spread in the modern diets, we saw a systematic increase in spread in the modern samples compared to the ancient diet (10%–20% increase). These findings exemplify gSpreadComp’s capacity to quantify and compare ARG spread across diverse samples. However, it is crucial to emphasize that these observations showcase the tool’s capabilities rather than draw definitive conclusions about diet–resistance relationships. The patterns identified by gSpreadComp can serve as starting points for more comprehensive studies, incorporating additional data sources and experimental validation to fully understand the complex interplay between diet and antimicrobial resistance.

### Virulence factor and resistance-virulence risk-ranking

Our results revealed a nuanced relationship between diet, the distribution of VFs, and the calculated resistance-virulence potential risk in the human gut microbiome. The average number of unique VFs was statistically similar among the diets. However, *Bacteroidota* associated with subjects from the ketogenic diet had a statistically higher number of unique VFs than subjects with other diets. Moreover, bacteria with high virulence potential consistently exhibited the highest number of unique antibiotic resistances, irrespective of the subject’s diet. Although alarming, this might be expected, as pathogenic bacteria should constantly be exposed to selective pressure.

In ranking relative resistance-virulence potential risk in our dataset, the tool consistently ranked higher risk to known potential pathogenic species. Interestingly, the subtle effects of diet on risk are evidenced in the *Firmicutes* phylum. A risk difference emerged between omnivores and vegetarians/vegans, and similarly between those on the ketogenic diet and vegetarians/vegans. However, no significant risk disparity was observed when comparing meat-consuming and nonmeat diets. These observations demonstrate gSpreadComp’s ability to detect nuanced patterns that could inform more targeted investigations.

Finally, our data indicated that vegans and vegetarians have significantly more ARGs and VFs involved in potential plasmid-mediated HGT than ancient, omnivore, and ketogenic groups. Specifically, a higher HGT potential was observed for the *Ruminococcaceae* and *Lachnospiraceae* families. These findings echo some of the discoveries by Reid et al. [49], which highlighted the predilection of produce from supermarkets to harbor *Escherichia coli* strains endowed with virulence plasmid carriage, thereby providing a potential conduit for HGT. Reid et al. [49] also discussed the possibility of producing drug-resistant *E. coli* from animal manure fertilizers, contaminated irrigation water, and wildlife. Specifically, they characterized resistant *E. coli* from supermarket-bought, ready-to-eat cilantro, arugula, and mixed salad from 2 German cities [49]. Another study underscored produce as a reservoir of transferable antibiotic resistance genes, further elucidating the plausible link between plant-based diets and amplified incidences of ARG in plasmid-mediated HGT owing to higher exposure to the transferable resistome inherent in produce [48]. Blau et al. [48] found an impressive diversity of self-transmissible multiple resistance plasmids in bacteria associated with produce that is consumed raw. Finally, Blau et al. [48] discussed the possibility of multiple resistance plasmids being exogenously captured by *E. coli* and transferred to gut bacteria, thus spreading resistance.

Although, to the best of our knowledge, no direct study comparing the abundance of plasmids in the human gut and soil was made, several studies indicated the potential increase in the abundance of plasmids in soil environments [62, 63]. Therefore, we hypothesize that gut microbiomes from plant-based diets have a higher chance of participating in plasmid-mediated HGT and indicate that targeted research should be performed to confirm or deny this hypothesis.

### Tools comparison

In comparative genomics, gSpreadComp gives a step forward as a tool that integrates genome annotation, gene spread calculation, virulence factor identification, plasmid-mediated HGT detection, and antimicrobial resistance-virulence risk-ranking. While previously mentioned existing tools have limitations, such as applicability to single taxa or reliance on reference genomes, gSpreadComp offers a comprehensive approach to applying comparative genomics to the entire microbiome. To our knowledge, PathoFact [42] and MetaCHIP [25] are the closest counterparts to gSpreadComp; however, they have different focal points (Table 3). PathoFact focuses on virulence and resistance gene prediction, while MetaCHIP can detect HGT events directly in a microbiome community in a reference-independent way. gSpreadComp focuses on these approaches, while offering a comprehensive analysis platform for microbial genomic studies.

**Table 3:** Feature comparison of gSpreadComp, PathoFact, and MetaCHIP across 4 key dimensions. Each tool offers distinct capabilities: gSpreadComp provides integrated metadata analysis with resistance-virulence risk-ranking, comparative genomics, and plasmid-mediated gene transfer detection; PathoFact specializes in antimicrobial resistance, virulence factors, toxins, and mobile genetic elements annotation; and MetaCHIP focuses on robust horizontal gene transfer detection within microbial communities. This comparison highlights complementary strengths that researchers can select based on their specific research questions.

Tool	Inputs	Analysistypes	Key outputs	Interpretability
gSpreadComp	MAGs/genomes with target metadata	ARG/VF annotation, plasmid detection, gene spread calculation, resistance-virulence risk-ranking	ARG and VF annotation, target gene spread calculation within the metadata groups; potential plasmid-mediated HGT events of ARG/VF in the community, resistance-virulence risk-ranking	Integrates metadata context, statistical comparison among metadata groups, provides relative risk-ranking within communities, HTML visual reports accessible to nonspecialists
PathoFact	Assembly FASTA files	ARG/VF, bacterial toxins genes, plasmid and phages detection	ARG/VF/toxin predictions with confidence levels, secretion status	Detailed annotation table ready for further analysis
MetaCHIP	MAGs/genomes with taxonomic classifications	Robust community-level HGT identification	HGT events within the community	Focuses on technical HGT outputs

gSpreadComp and PathoFact both target ARG, VF, and MGE annotation in microbial genome analysis, sharing similar objectives. Both approaches use key tools like PlasFlow for plasmid identification, DeepARG for antimicrobial resistance gene annotation, and the Virulence Factors Database (VFDB) for annotating virulence factors, which yield similar results in these aspects. However, gSpreadComp adds a unique dimension with its resistance-virulence risk-ranking using TOPSIS, gene spread calculation, and detailed downstream analysis. PathoFact, on the other hand, emphasizes precision in virulence and toxin prediction through a blend of HMM profiles and machine learning approaches.

Against MetaCHIP, gSpreadComp focuses on plasmid-mediated HGT. While MetaCHIP provides robust HGT detection by combining similarity and phylogenetic approaches, gSpreadComp adds value by directly linking these events to sample metadata, which is crucial for comparative genomics and useful for nonspecialist users like clinicians. Naturally, the HGT events detected by gSpreadComp should be present in the results from MetaCHIP.

gSpreadComp’s streamlined approach makes it a versatile tool that addresses gaps left by existing methodologies. The approach is particularly advantageous for non-bioinformaticians, as it simplifies complex analyses, making the data accessible and actionable for a broader audience. While gSpreadComp offers a comprehensive approach, it is not intended to replace specialized tools. Instead, it aims to complement existing methodologies by providing an integrated approach for microbial genomic analysis. Users should consider their specific research questions and requirements when choosing the most appropriate tool or combination of tools for their studies. The analyses performed using gSpreadComp are not conclusive but serve to raise testable hypotheses and focus subsequent laboratory experimentation. By identifying potential antimicrobial resistance and virulence factors, along with their likely bacterial hosts, gSpreadComp narrows the search space for targeted experimental validation.

## Conclusion

gSpreadComp combines genome annotation, gene prevalence normalization, and target (i.e., diet) analysis into a comprehensive workflow for quantifying gene spread and assessing potential resistance-virulence risk-ranking in microbial communities. The tool’s modular design allows for flexibility and future updates. The tool’s application to explore dietary impacts on gut microbiome antibiotic resistance demonstrated its ability to identify complex patterns across different dietary groups. Moreover, nuanced evidence suggested that meat and uncooked produce influence resistance-virulence spread, particularly concerning plasmid-mediated HGT, emphasizing the intricate relationship between diet and microbial dynamics in the human gut. However, it is crucial to emphasize that these findings are intended to showcase gSpreadComp’s capabilities rather than draw definitive conclusions about diet–resistance relationships.

The patterns identified by gSpreadComp can serve as valuable starting points for more comprehensive studies, incorporating larger sample sizes or focused experiments, additional data sources, and experimental validation. As with any bioinformatics tool, results should be interpreted cautiously and used to guide hypothesis generation and further investigation. gSpreadComp aims to complement existing methodologies by providing an integrated platform for microbial genomic analysis, potentially benefiting a wide range of users.

## Data and Methods

### Implementation

#### The gSpreadComp

gSpreadComp is designed for UNIX-based systems. The user can refer to the manual [64] for detailed instructions. Fundamentally, our approach works in 6 modular steps: (i) prokaryotic genome taxonomy assignment, (ii) genome quality estimation, (iii) ARG annotation, (iv) plasmid and chromosome classification, (v) VF annotation, and (vi) downstream analysis, which involves target-based gene spread analysis, plasmid-mediated HGT of the target gene and VF, prokaryotic resistance-virulence risk-ranking, and report generation.

Each module can be applied separately. Consequently, as new sequence classification tools surge, gSpreadComp downstream analysis can continue to be used independently. Another advantage of a modular implementation is that the approach can be easily updated. Figure 1 indicates the gSpreadComp structure. The approach was written in Bash and R (version 4.2.2) [65]. Finally, we use conda [66] (conda 22.11.1) environments to install all necessary software dependencies and third-party software wherever possible. Using conda allows software management with different and potentially conflicting dependencies in the same system. In the future, we will develop a Singularity container [67] to facilitate installation and ensure reproducibility across diverse computing infrastructures.

In step (i), the user can directly assign taxonomy using GTDB-tk [68] and format the result table automatically. In step (ii), gSpreadComp orchestrates CheckM [69] to estimate prokaryotic genome quality and format the resulting files. Following step (iii), the user can automatically annotate ARG and format its resulting files. To minimize the risk of false-positive ARG prediction, gSpreadComp uses the DeepARG-LS [32] with the following parameter values: a minimum of 80% prediction probability, an e-value alignment lower than 1e-10, and a percent identity of 35% or higher [33].

In step (iv), plasmids are predicted using PlasFlow with default parameters (i.e., 0.7 probability threshold) [34]. PlasFlow uses only genomic signatures to identify bacterial plasmids using a neural network model with increased performance compared to similar tools [34]. In addition, this tool is also optimized for metagenomic data, the type of data we expect to use mainly with gSpreadComp. Then, in step (v), we use the Victors VF database (downloaded in December 2022) [70] and the Virulence Factors Database (downloaded in December 2022) [71] to annotate VF on provided genomes. We use the protein sequences from both databases from their core dataset associated with experimentally verified virulence factors. We use BLASTX [72] with an e-value of 1e-50 as the cutoff to locate the VFs.

Finally, in step (vi), gSpreadComp starts by optionally filtering out genomes based on the quality (Completeness – 5 * Contamination > 50). It can then remove samples based on the total number of genomes per sample (by default, no sample is removed). Next, we calculated the normalized prevalence of the target gene in a defined group (${{\boldsymbol{P}}}_{{\boldsymbol{group}},{\boldsymbol{\ \textit{gene}}}}$). It considers the presence or absence of the target gene in a genome divided by the total number of genomes in a group, similar to the definition used by Danko et al. [4]. A Bonferroni-adjusted *t*-test is used pairwise to compare the target gene prevalence across the groups. When the adjusted *P*-value was less than 0.05, we assigned a significant difference between the groups. The user can refer to the manual [64] for a detailed description of the intermediate files generated.


\begin{eqnarray*}
{{\boldsymbol{P}}}_{{\boldsymbol{group}},{\boldsymbol{\ \textit{gene}}}} = {\boldsymbol{\ }}\frac{{\sum {\boldsymbol{Genom}}{{\boldsymbol{e}}}_{{\boldsymbol{group}},{\boldsymbol{\ \textit{gene}}}}}}{{\sum {\boldsymbol{Genom}}{{\boldsymbol{e}}}_{{\boldsymbol{group}}}}}
\end{eqnarray*}

We use the defined WAP to estimate the gene spread per taxonomical level per target metadata group, as described by Magnúsdóttir et al. [31]. ${{\boldsymbol{P}}}_{{\boldsymbol{i\ }}}$ is the gene prevalence per specified taxonomical group, and ${\boldsymbol{T}}$ is the number of unique taxa in the defined taxonomical level.


\begin{eqnarray*}
{\boldsymbol{WAP\ }} = \mathop \sum \nolimits_{{\boldsymbol{i}} = 1}^{\boldsymbol{T}} \frac{{{{\boldsymbol{P}}}_{{\boldsymbol{i\ }}} \times {\boldsymbol{\ }}\sum {\boldsymbol{Genom}}{{\boldsymbol{e}}}_{\boldsymbol{i}}{\boldsymbol{\ }}}}{{\boldsymbol{T}}}
\end{eqnarray*}

Finally, gSpreadComp extracts what we defined as “resistance-virulence risk factors” for each genome. Those are the genetic potential related to the target gene, represented by the number of unique target genes; the virulence potential, represented by the number of unique VFs; the potential of transmitting the target gene, represented by the number of unique target genes located in plasmids; and the potential of transmitting virulence potential, represented by the number of unique VFs located in plasmids. We use the taxonomical distances to the species in the NCBI pathogens database [27] to define the reference potential pathogens. Finally, we use the TOPSIS [28] to rank the resistance-virulence risk from the genomes. Essentially, we extract from each genome (${{\boldsymbol{g}}}_{\boldsymbol{i}}$) its resistance-virulence risk factors (${{\boldsymbol{f}}}_{\boldsymbol{j}}$), ${{\boldsymbol{g}}}_{\boldsymbol{i}} = \{{{{\boldsymbol{f}}}_{{\boldsymbol{i}},1},{{\boldsymbol{f}}}_{{\boldsymbol{i}},2},\ldots,{{\boldsymbol{f}}}_{{\boldsymbol{i}},{\boldsymbol{n}}}} \}$, with *n* resistance-virulence risk factors.

Following this, we normalized the resistance-virulence risk factors using


\begin{eqnarray*}
{{\boldsymbol{f}}}_{{\boldsymbol{ij}}} = \frac{{{{\boldsymbol{f}}}_{{\boldsymbol{ij}}}}}{{\sqrt {\mathop \sum \nolimits_{{\boldsymbol{i}} = 1}^{\boldsymbol{m}} {\boldsymbol{f}}_{{\boldsymbol{ij}}}^2} }}
\end{eqnarray*}

where ${{\boldsymbol{f}}}_{{\boldsymbol{ij}}}$ is the value of the ${{\boldsymbol{j}}}^{{\boldsymbol{th}}}$ risk factor for the ${{\boldsymbol{i}}}^{{\boldsymbol{th}}}$ genome, and ${\boldsymbol{\ m}}$ is the total number of genomes. Then, we computed the weighted normalized decision matrix. The defined weights, ${\boldsymbol{W}} = \{ {{{\boldsymbol{w}}}_1,{{\boldsymbol{w}}}_2,\ldots{{\boldsymbol{w}}}_{\boldsymbol{n}}} \}$, are the average of the resistance-virulence risk factors extracted from the reference potential pathogens. The weighted normalized decision matrix is represented by


\begin{eqnarray*}
{{\boldsymbol{v}}}_{{\boldsymbol{ij}}} = {{\boldsymbol{w}}}_{\boldsymbol{j}} \times {{\boldsymbol{r}}}_{{\boldsymbol{ij}}}
\end{eqnarray*}

We defined the ideal, ${{\boldsymbol{A}}}^{\boldsymbol{*}} = \{ {{\boldsymbol{v}}_1^{\boldsymbol{*}},{\boldsymbol{v}}_2^{\boldsymbol{*}},\ldots{\boldsymbol{v}}_{\boldsymbol{n}}^{\boldsymbol{*}}} \}$, and the negative-ideal, ${{\boldsymbol{A}}}^ - = \{ {{\boldsymbol{v}}_1^ - ,{\boldsymbol{v}}_2^ - ,\ldots{\boldsymbol{v}}_{\boldsymbol{n}}^ - } \}$, solutions as ${\boldsymbol{v}}_{\boldsymbol{j}}^{\boldsymbol{*}} = \mathop {\max }\limits_{\boldsymbol{i}} ( {{{\boldsymbol{v}}}_{{\boldsymbol{ij}}}} )$ and ${\boldsymbol{v}}_{\boldsymbol{j}}^ - = \mathop {\min }\limits_{\boldsymbol{i}} ( {{{\boldsymbol{v}}}_{{\boldsymbol{ij}}}} )$.

Next, for each genome, we calculated the separation from the ideal solution $( {{\boldsymbol{S}}_{\boldsymbol{i}}^{\boldsymbol{*}}} )$ and from the negative-ideal solution $( {{\boldsymbol{S}}_{\boldsymbol{i}}^ - } )$ as


\begin{eqnarray*}
{\boldsymbol{S}}_{\boldsymbol{i}}^{\boldsymbol{*}} = \sqrt {\mathop \sum \limits_{{\boldsymbol{j}} = 1}^{\boldsymbol{n}} {{\left( {{{\boldsymbol{v}}}_{{\boldsymbol{ij}}} - {\boldsymbol{v}}_{\boldsymbol{j}}^{\boldsymbol{*}}} \right)}}^2}
\end{eqnarray*}

\begin{eqnarray*}
{\boldsymbol{S}}_{\boldsymbol{i}}^ - = \sqrt {\mathop \sum \limits_{{\boldsymbol{j}} = 1}^{\boldsymbol{n}} {{\left( {{{\boldsymbol{v}}}_{{\boldsymbol{ij}}} - {\boldsymbol{v}}_{\boldsymbol{j}}^ - } \right)}}^2}
\end{eqnarray*}

Finally, the prokaryotic risk (${{\boldsymbol{R}}}_{\boldsymbol{i}}$) is the relative closeness to the ideal solution.


\begin{eqnarray*}
{{\boldsymbol{R}}}_{\boldsymbol{i}} = \frac{{{\boldsymbol{S}}_{\boldsymbol{i}}^ - }}{{{\boldsymbol{S}}_{\boldsymbol{i}}^{\boldsymbol{*}} + {\boldsymbol{S}}_{\boldsymbol{i}}^ - }}
\end{eqnarray*}

The genome with the highest ${{\boldsymbol{R}}}_{\boldsymbol{i}}$ value ranks higher in the microbial community resistance-virulence risk scale. We used the TOPSIS implementation in the MCDA R package.

To extract the plasmid-mediated HGT events, we implemented a similar heuristic in gSpreadComp, as defined by Smillie et al. [73]. Briefly, 1 recent HGT event could be identified between 2 distantly related genomes (from a defined taxonomical level) through the shared region of DNA corresponding to an annotated sequence with 99% or greater similarity.

Lastly, gSpreadComp uses the files, metrics, and figures to generate an HTML report automatically from the rmarkdown [74] package.

#### Use case: gSpreadComp in the human gut microbiome of subjects with different diets

The gSpreadComp approach requires genomes or MAGs in fasta format; the genomes metadata table, including the identification of its source sample and the target feature to be compared; a genome taxonomic assignment table; a genome quality assignment table; and a target gene annotation table.

### Metagenome data selection

Initially, we selected metagenomic samples from the human gut of subjects over 18 years old containing information about the host diet using the HumanMetagenomeDB (HMgDB) [75]. We selected only WGS libraries available in the Sequence Read Archive (SRA) [76]. After filtering, we had metagenomic samples from the following BioProjects: PRJNA340216, PRJNA397112, PRJNA324129, and PRJNA529487. Afterward, we examined the sample’s metadata information on the original studies and assigned the libraries in “omnivore,” “vegetarian,” “vegan,” and “ketogenic” diet types according to the original studies’ definitions. Additionally, we included metagenomic libraries from the AncientMetagenomeDir v20.12 [77]. From the libraries provided on the ancientmetagenome-hostassociated file, we selected those with the following parameters: “sample_host” equal to “*Homo sapiens*,” “community_type” equal to “gut,” and “archive” equal to “ENA” or “SRA.” We assigned libraries that originated from the AncientMetagenomeDir as “ancient.” The complete table of libraries and accompanying metadata used is in Additional File 1: Supplementary Table S1. Finally, we downloaded the library reads from the SRA using the SRAtoolkit version 2.10.9 [78].

### Data preparation

The MAGs were recovered using the Multi-Domain Genome Recovery tool (MuDoGeR) [79]. The raw reads were quality-controlled using metaWrap [80] with default parameters. The reads trimming was performed using TrimGalore (RRID:SCR_011847) [81] with the default settings. After, BMTagger (RRID:SCR_014619) [82] was used with the human build 38 patch release 13 (GRCh38.p13 [83]) to remove potential host genomes using default parameters. Then, reads were assembled using metaSpades [84] from within the MuDoGeR approach. Once assembled, the sequence contigs were binned using Metabat2 [85], Maxbin2 [86], and CONCOCT [87]. Next, the recovered bins were refined and dereplicated using MuDoGeR. The bins were quality-checked using CheckM (RRID:SCR_016646) [69] and taxonomically assigned using GTDB-tk (RRID:SCR_019136) [68], and assembly statistics were calculated with BBTools [88]. Finally, the bins were filtered for MAGs based on the following criteria: at least 50% completeness, less than 10% contamination based on CheckM results, and a quality score higher than or equal to 50, where quality score = completeness − 5 * contamination [89]. High-quality MAGs were defined as completeness >90% and contamination <5%. Medium-quality MAGs were defined as completeness ≥50% and contamination <10%. Then, we used the ARG annotation workflow from gSpreadComp to annotate ARGs in each MAG. This annotation step means we used DeepARG-LS with a minimum of 80% prediction probability, an e-value alignment lower than 1e-10, and a percent identity of 35% or higher to minimize the risk of false positives. Next, we used the gSpreadComp methods described earlier to classify plasmid sequences and annotate and format VFs. We removed samples with fewer than 6 genome representatives to calculate the gene prevalence per sample, as a lower number of recovered genomes typically indicates insufficient sequencing depth [29], which can introduce statistical bias and skew prevalence analyses to values significantly different from those that would be obtained with adequate genome representation. Finally, we integrated the recovered MAGs and the following tables into the gSpreadComp approach: formatted taxonomic assessment, the prokaryotic quality estimation, the ARG annotation, the plasmid identification, the VF annotation, and the library metadata. In addition, we also used the gSpreadComp approach to estimate the spread of the ARG antibiotic resistance group (e.g., bacitracin and glycopeptide), hereafter referred to as ARG classes.

## Availability of Source Code and Requirements

Project name: gSpreadComp

Project homepage: https://github.com/mdsufz/gSpreadComp/

Operating system(s): Linux

Programming language: C, Shell, R, Python

Other requirements: Bash, Conda, Mamba, and other packages automatically installed with gSpreadComp

License: GNU GPL v3.0

RRID: SCR_026,798

A version of record snapshot of the GitHub repository has been archived in the Software Heritage Library (PID swh:1:dir:26ba1978f7b6cf8eb968e3728d4adee62fa4034e [90]).

The tool is also available via WorkflowHub [91].

## Supplementary Material

giaf072_Supplemental_Files

giaf072_Authors_Response_To_Reviewer_Comments_original_submission

giaf072_Authors_Response_To_Reviewer_Comments_Revision_1

giaf072_GIGA-D-24-00460_original_submission

giaf072_GIGA-D-24-00460_Revision_1

giaf072_GIGA-D-24-00460_Revision_2

giaf072_Reviewer_1_Report_Original_Submission Veronica Jarocki -- 12/9/2024

giaf072_Reviewer_1_Report_Revision_1 Veronica Jarocki -- 4/3/2025

## Data Availability

MAGs, plasmid, chromosomes identified sequences, ARG alignments and database sequences, and VF annotation and reference database sequences generated and used in this study can be downloaded at [92]. All MAGs are publicly available from the National Institutes of Health under the BioProject PRJNA1032156. The genomic sequence data used in this study are available under the following BioProject accessions: PRJNA340216, PRJNA397112, PRJNA324129, PRJNA529487. Supporting data are also available via the *GigaScience* repository, GigaDB [93].

## Additional Files

**Additional File 1**. 01_Kasmanas_gSpread_AddFile1_Table_S1.xlsx

**Additional File 2**. 02_Kasmanas_gSpread_AddFile2_Table_S2.xlsx

**Additional File 3**. 03_Kasmanas_gSpread_AddFile3_Table_S3.xlsx

**Additional File 4**. 04_Kasmanas_gSpread_AddFile4_Fig_S1.docx

**Additional File 5**. 05_Kasmanas_gSpread_AddFile5_Table_S4.xlsx

**Additional File 6**. 06_Kasmanas_gSpread_AddFile6_Fig_S2.docx

**Additional File 7**. 07_Kasmanas_gSpread_AddFile7_Table_S5.xlsx

**Additional File 8**. 08_Kasmanas_gSpread_AddFile8_Fig_S3.docx

**Additional File 9**. 09_Kasmanas_gSpread_AddFile9_Table_S6.xlsx

**Supplementary Fig. S1**. Boxplots from the ARG class prevalence per sample (y-axis) colored by Target Diet. The boxplot title is the ARG class. The statistically significant pairwise comparisons are indicated with the * symbol.

**Supplementary Fig. S2**. Heatmaps containing the spread, calculated as weighted average prevalence (WAP) of the antimicrobial resistance gene (ARG) classes (rows) per phylum (columns) per target diet (title). The number within parentheses after the phylum indicates the number of genomes used for the calculation from that phylum. The number within parentheses from the ARG classes is the average spread for that ARG class.

**Supplementary Fig. S3**. (a) Boxplots colored by target diet. The x-axis is grouped by pathogenic potential defined by the taxonomical distance to potential pathogens from the NCBI pathogen database. The y-axis is the number of unique virulence factors (VF) per sample. (b) Group of boxplots per phylum that are common to all target diets. The x-axis is grouped and colored by target diet. The y-axis has the calculated resistance-virulence risk metric. (c) Density plots of the resistance-virulence risk for each common phylum colored by target diet. The y-axis indicates the estimated probability density of the respective resistance-virulence risk on the x-axis. Density plots are calculated using the seaborn.kdeplot in Python 3.9. (d) Boxplot for the number of antimicrobial resistance genes (ARGs) involved in plasmid-mediated horizontal gene transfer (HGT) events found per sample on the y-axis. The x-axis is grouped and colored by target diet. (e) Boxplot for the number of VFs involved in plasmid-mediated HGT events found per sample on the y-axis. The x-axis is grouped and colored by target diet.

**Supplementary Table S1**. Metadata table from the selected whole-genome sequencing (WGS) samples. Columns are standardized as described by Kasmanas et al. [75]. Samples collected from the AncientMetagenomeDir had the host_diet assigned as “Ancient.” The “sample” column is equivalent to the SRA project_id.

**Supplementary Table S2**. (a) Summary information retrieved from the recovered metagenome-assembled genomes (MAGs). Completeness, Contamination, and Strain.heterogeneity are assigned with CheckM through MuDoGeR [79]. Quality and quality.score are determined as described in Methods. The Target column refers to the source patient’s diet. The taxonomical information was assigned with GTDBtk through MuDoGeR. Pathogen potential is determined based on the taxonomical distance to reference potential pathogens from the NCBI pathogen database. The risk_criteria ranks the relative resistance-virulence risk calculated as described in Methods. The columns named “unique_*” are defined as “Resistance-virulence Risk Factors” and are used to rank the relative resistance-virulence risk. The Factors are systematically named as follows: “unique_,” virulence factors (vf) or target gene (ARGs in our use case), “_in_,” sequence type location (i.e., chromosome, plasmids, or unclassified). The last 19 columns are assembly statistics extracted using BBTools [88]. (b) Distribution of the number of MAGs per diet per quality.

**Supplementary Table S3**. (a) DeepARG [32] antimicrobial resistance gene (ARG) annotation table. gSpreadComp expects to receive a gene annotation csv table in a similar format, indicating the Genome column as “Genome,” the target gene column as “Gene_id,” and the sequence name from the fasta file where the gene was annotated as “Gene_sequence_location.” The probability and identity columns are defined by DeepARG. The “probability” column is the probability that the gene annotation is correct according to their highly accurate ARG predicting model. (b) Target gene prevalence normalization table per sample (Library). The target gene was the ARG class (Gene_class) from the DeepARG annotation table. The present.gene column indicates how many metagenome-assembled genomes (MAGs) in that Library had the specified Gene_class annotated. The Target column indicates the diet from the Library. The t_mags column indicates the total number of MAGs recovered, and the gene.genome.prev column indicates the prevalence of the Gene_class. (c) Bonferroni-adjusted *t*-test pairwise comparison from the ARG class (Gene_class) prevalence per diet. The y column shows the variable’s name used in comparing group1 and group2. The n1 and n2 columns show the number of samples compared. The statistic column is the resulting *t*-test statistic, and df is the degrees of freedom associated with the test. The p is the *P*-value from the comparison, p.adj is the Bonferroni-adjusted result, and p.adj.signif is an indication of significance (*P* < 0.05).

**Supplementary Table S4**. (a) Antimicrobial resistance gene (ARG) class, as assigned by DeepARG [32], spread at the phylum level per target diet. The spread was calculated using the weighted average prevalence (WAP). (b) The ARG class spread, calculated using WAP per phylum for the Ancient diet. (c) The ARG class spread, calculated using WAP per phylum for the ketogenic diet. (d) The ARG class spread, calculated using WAP per phylum for the omnivore diet. (e) The ARG class spread, calculated using WAP per phylum for the vegan diet. (f) The ARG class spread, calculated using WAP per phylum for the vegetarian diet.

**Supplementary Table S5**. (a) Virulence factors (VFs) from the Victors virulence factors database [70] (downloaded in December 2022) annotated on the Genomes (Genome column) recovered from the whole-genome sequence (WGS) samples (Library) using BLASTX. Sequence_id indicates the sequence header where the VF (Victor_VF_found) was aligned. Victor_VF_class is the class of the VF given by Victors database. The e-value and bitscore are aligning metrics provided by BLASTX. (b) The average number of unique VFs per phylum per target diet (column avg_unique_VFs). The n column indicates the number of samples used for the calculation, and the column sd_unique_VFs shows the standard deviation from the calculated metrics. (c) All statistically significant Bonferroni-adjusted *t*-test pairwise comparisons from the unique number of VFs grouped per phylum per target diet. The comparison was made between the diets indicated in group1 and group2. The n1 and n2 columns show the number of samples compared. The p is the *P*-value from the comparison, p.adj is the Bonferroni-adjusted result, and p.adj.signif is an indication of significance (*P* < 0.05). The unique number of VFs per genome can be found in Supplementary Table S2a. (d) All statistically significant Bonferroni-adjusted *t*-test pairwise comparisons from the unique number of antimicrobial resistance genes (ARGs) grouped per pathogenic potential based on the NCBI pathogens database. The comparison was made between the pathogenic potential indicated in group1 and group2. The n1 and n2 columns show the number of samples compared for group1 and group 2, respectively. The p is the *P*-value from the comparison, p.adj is the Bonferroni-adjusted result, and p.adj.signif is an indication of significance (*P* < 0.05). Values equal to 0 were extremely close to 0. The unique number of ARGs per genome can be found in Supplementary Table S2a. (e) All statistically significant Bonferroni-adjusted *t*-test pairwise comparisons from the resistance-virulence risk per phylum grouped per target diet. The comparison was made between the target diets indicated in diet 1 and diet 2. The p.adj is the Bonferroni-adjusted *P*-value result. The resistance-virulence risk value per genome can be found in Supplementary Table S2a.

**Supplementary Table S6**. (a) List of identified antimicrobial resistance gene (ARG) plasmid-mediated horizontal gene transfer (HGT) events. The library is the sample where the event was found, and Family1 and Family2 are the taxonomical families involved in the event. The Gene_id column identifies the ARG name involved, and the Target column identifies the target diet from the respective library. (b) List of identified virulence factor (VF) plasmid-mediated HGT events. The Library is the sample where the event was found, and Family1 and Family2 are the taxonomical families involved in the event. The Gene_id column identifies the VF name from the Victors database [70] involved, and the Target column identifies the target diet from the respective library. (c) Summary from the HGT events per library per target diet after removing the libraries that recovered fewer than 12 metagenome-assembled genomes. (d) Bonferroni-corrected *t*-test pairwise comparison between the number of ARG HGT events grouped by the target diet after removing the libraries that recovered fewer than 12 metagenome-assembled genomes. Target 1 and Target 2 are the diets compared. *T*-statistic, *P*-value, and adjusted *P*-value are the statistical test results. (e) Bonferroni-corrected *t*-test pairwise comparison between the number of VF HGT events grouped by the target diet after removing the libraries that recovered fewer than 12 metagenome-assembled genomes. Target 1 and Target 2 are the diets compared. *T*-statistic, *P*-value, and adjusted *P*-value are the statistical test results. (f) Summary from the HGT events per family target diet after removing the libraries that recovered fewer than 12 metagenome-assembled genomes. (g) Bonferroni-corrected *t*-test pairwise comparison between the number of ARG HGT events per family grouped by the target diet after removing the libraries that recovered fewer than 12 metagenome-assembled genomes. Target 1 and Target 2 are the diets compared for the respective family. *T*-statistic, *P*-value, and adjusted *P*-value are the statistical test results. Sample sizes indicate the number of samples used for each target, respectively. (h) Bonferroni-corrected *t*-test pairwise comparison between the number of VF HGT events per family grouped by the target diet after removing the libraries that recovered fewer than 12 metagenome-assembled genomes. Target 1 and Target 2 are the diets compared for the respective family. *T*-statistic, *P*-value, and adjusted *P*-value are the statistical test results. Sample sizes indicate the number of samples used for each target, respectively.

## Abbreviations

AMR: antimicrobial resistance; ARB: antimicrobial-resistant bacteria; ARGs: antimicrobial resistance genes; HGT: horizontal gene transfer; MAGs: metagenome-assembled genomes; MLS: macrolides, lincosamides, streptogramines; SRA: Sequence Read Archive; TOPSIS: Technique for Order Preference by Similarity to Ideal Solution; VGT: vertical gene transfer; WAP: weighted average prevalence; WGS: whole-genome sequencing.
