# Supplementary material for: Integrating comparative genomics and risk classification by assessing virulence, antimicrobial resistance, and plasmid spread in microbial communities with gSpreadComp
Source: Gigascience. 2025 Jun 26;14:giaf072. doi: 10.1093/gigascience/giaf072 (PMC12199706; doi:10.1093/gigascience/giaf072)

Streamlining microbial community analysis for potential resistance, virulence, and plasmid-mediated spread through integrated comparative genomics and relative risk ranking using gSpreadComp

Jonas Coelho Kasmanas [a,b,c], Stefanía Magnúsdóttir [a], Junya Zhang [d], Kornelia Smalla [e], Michael Schloter[f], Peter F. Stadler [c], André Carlos Ponce de Leon Ferreira de Carvalho [b], Ulisses Rocha [a]#

[a] Department of Environmental Microbiology, Helmholtz Centre for Environmental Research – UFZ, Leipzig, Germany.

[b] Institute of Mathematics and Computer Sciences, University of São Paulo, São Carlos, Brazil.

[c] Department of Computer Science and Interdisciplinary Center of Bioinformatics, University of Leipzig, Leipzig, Germany.

[d] Department of Isotope Biogeochemistry, Helmholtz Centre for Environmental Research – UFZ, Leipzig, Germany.

[e] Julius Kühn-Institut, Federal Research Centre for Cultivated Plants, Institute for Epidemiology and Pathogen Diagnostics, Braunschweig, Germany

[f] Helmholtz Center Munich, National Research Center for Environmental Health, Insitute for Comparative Microbiome Analysis, Neuherberg, Germany

#Address correspondence to Ulisses Rocha, ulisses.rocha@ufz.de

FIG S1. Boxplots from the ARG class prevalence per sample (y-axis) colored by Target Diet. The boxplot title is the ARG class. The statistically significant pairwise comparisons are indicated with the * symbol.

FIG S1a.

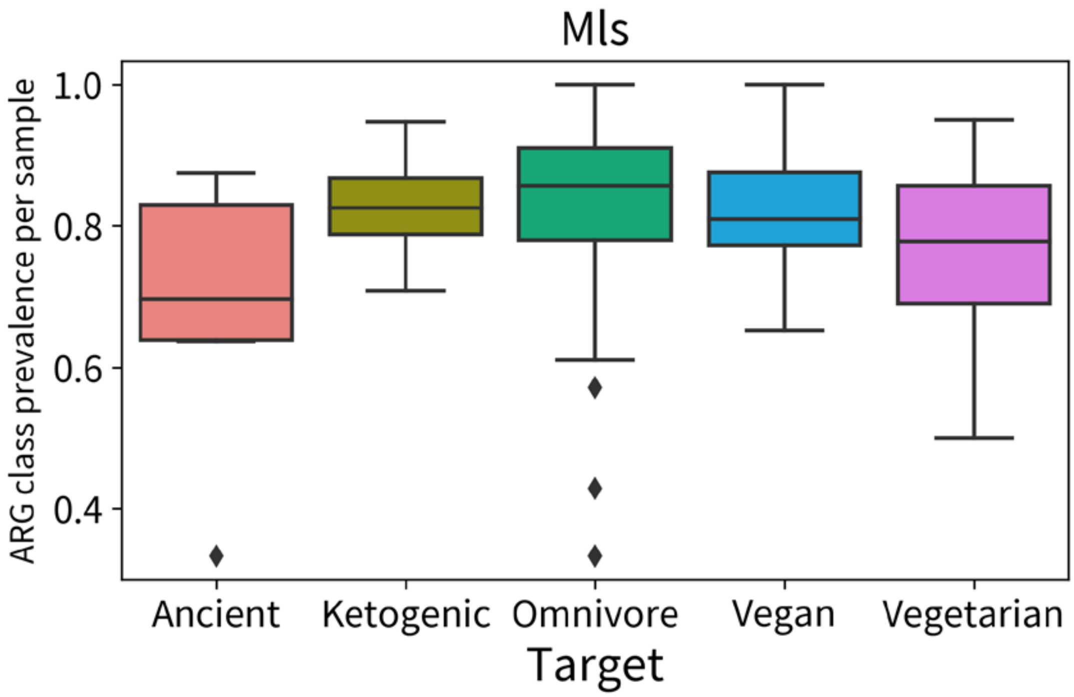

FIG S1b.

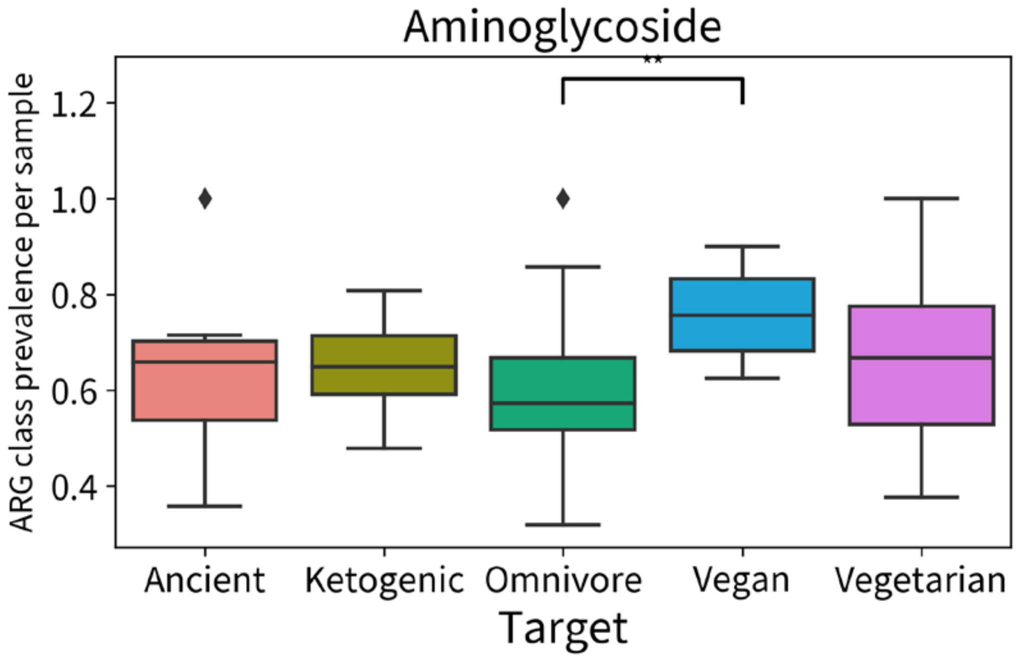

FIG S1c.

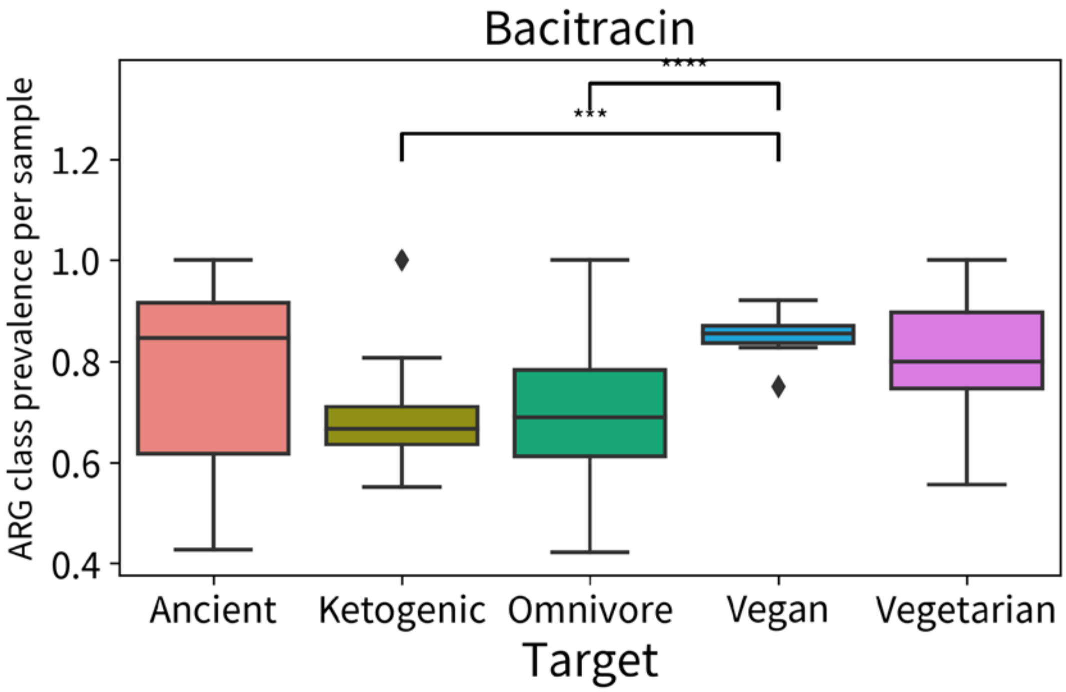

FIG S1d.

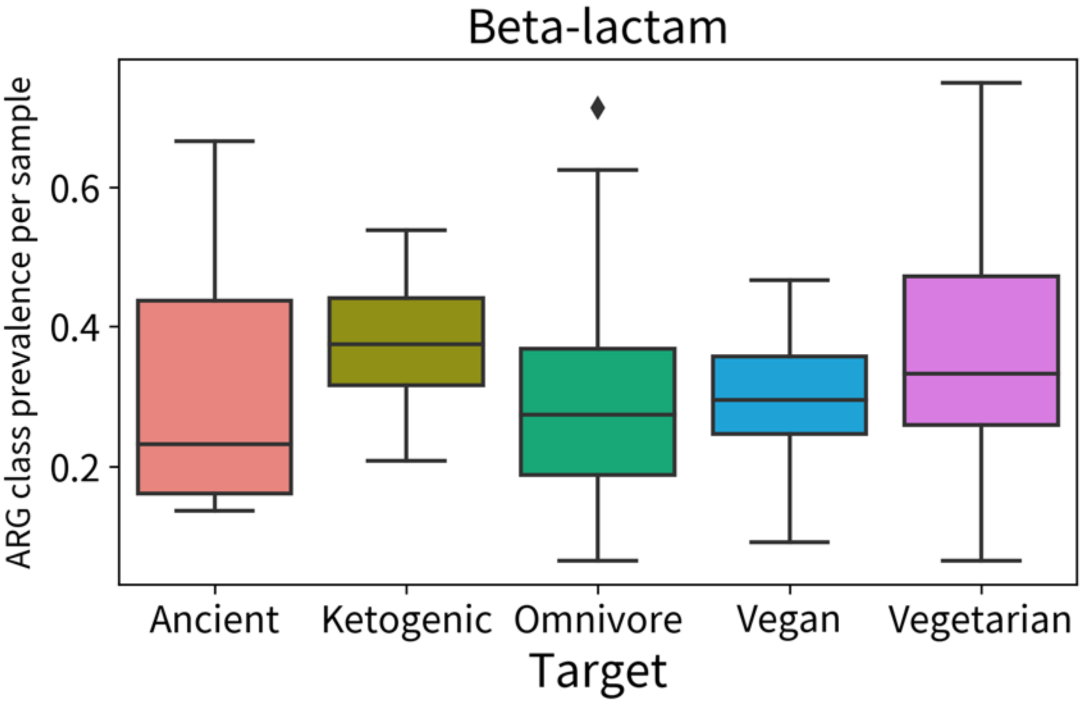
Beta-lactam
ARG class prevalence per sample
0.2
0.4
0.6
Ancient
Ketogenic
Omnivore
Vegan
Vegetarian
Target

FIG S1e.

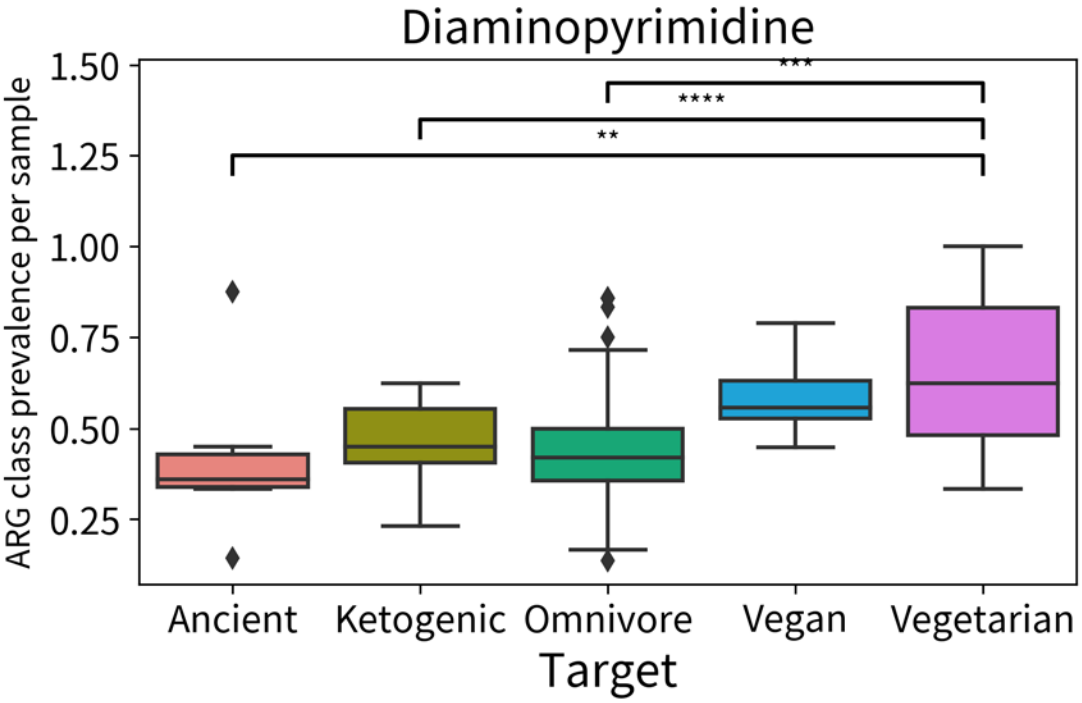
Diaminopyrimidine
ARG class prevalence per sample
0.25
0.50
0.75
1.00
1.25
1.50
***
****
**
Ancient
Ketogenic
Omnivore
Vegan
Vegetarian
Target

FIG S1f.

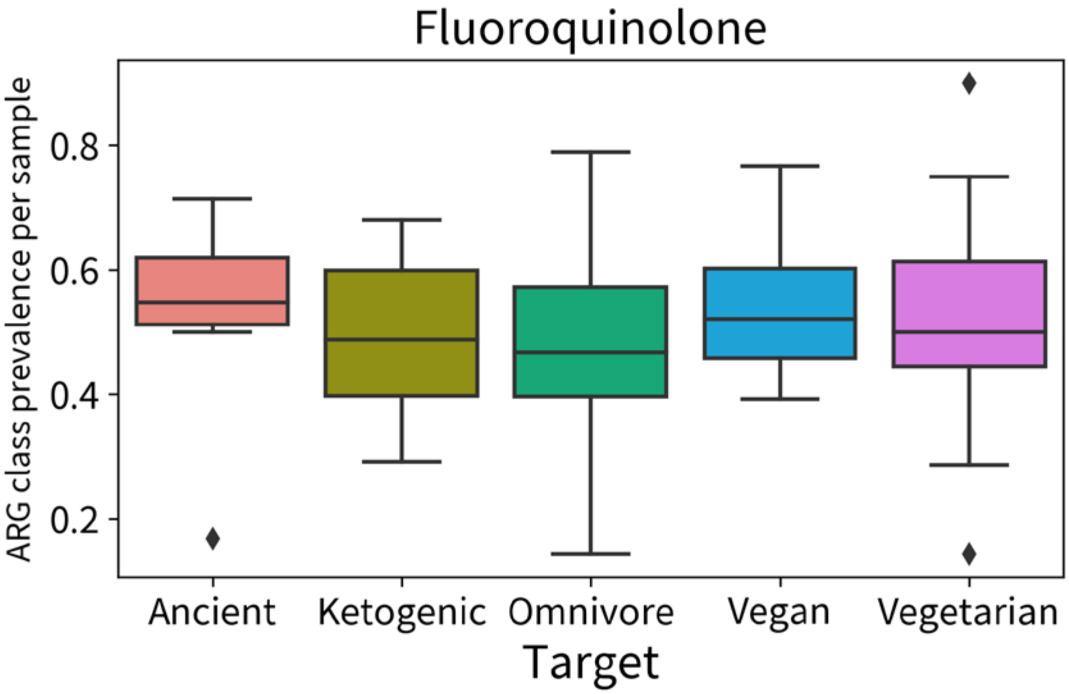

FIG S1g.

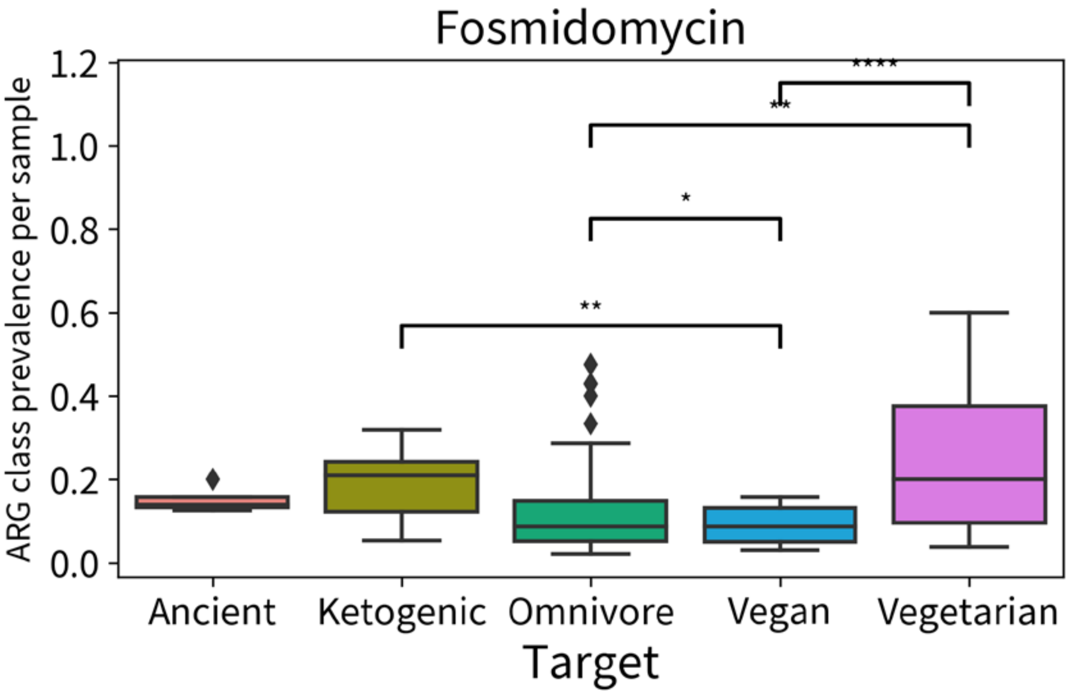

FIG S1h.

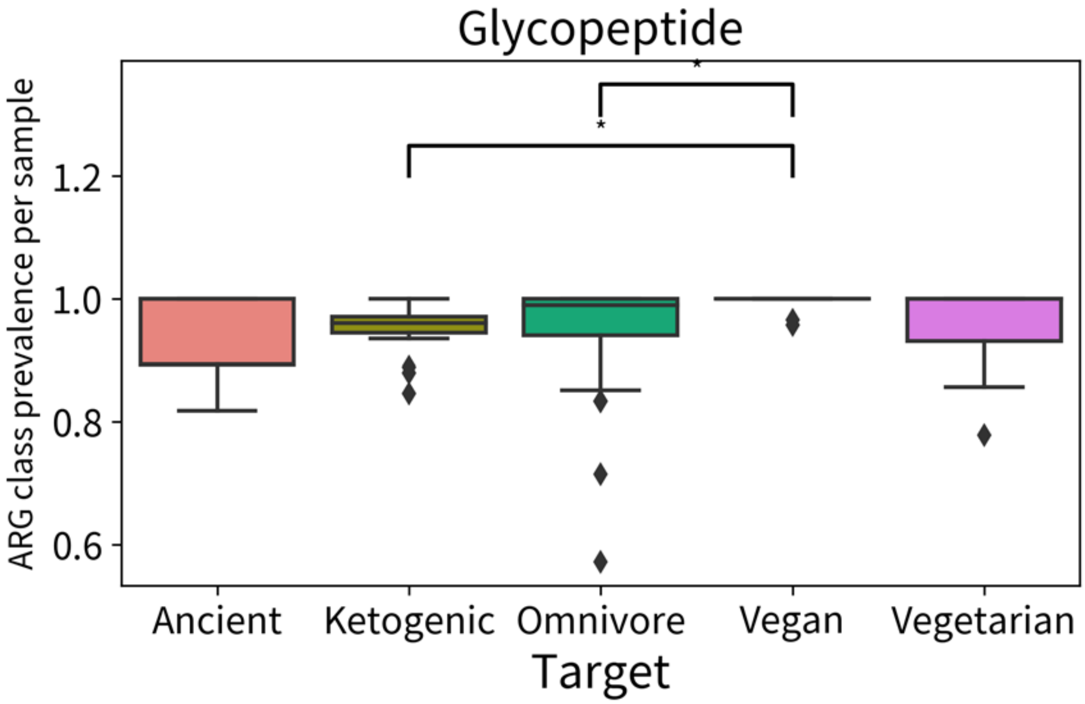

FIG S1i.

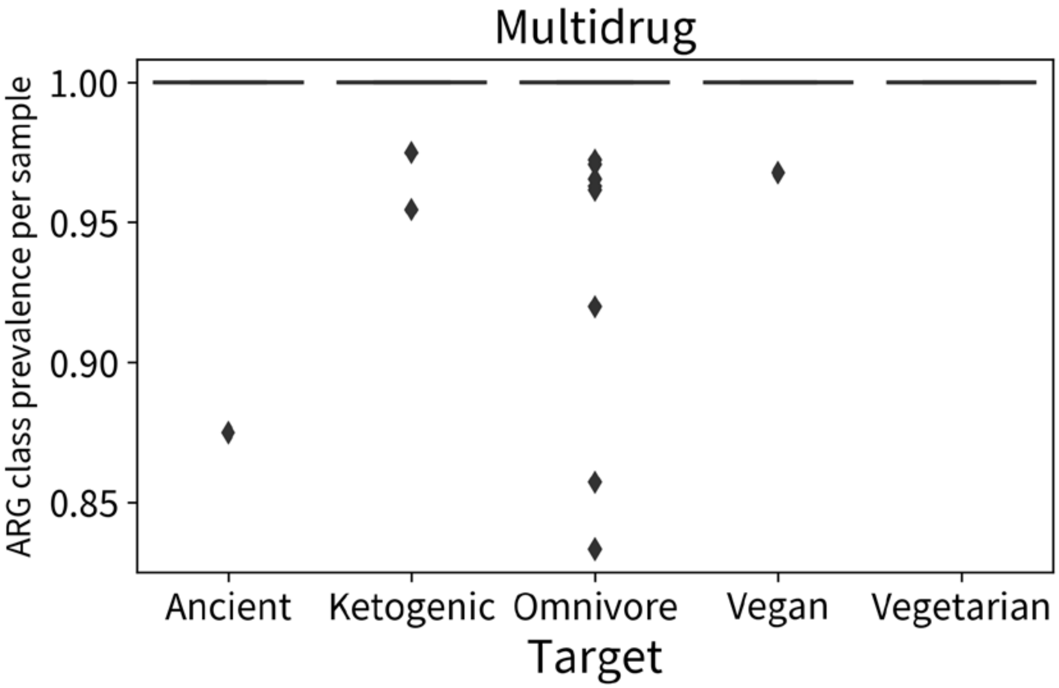

FIG S1j.

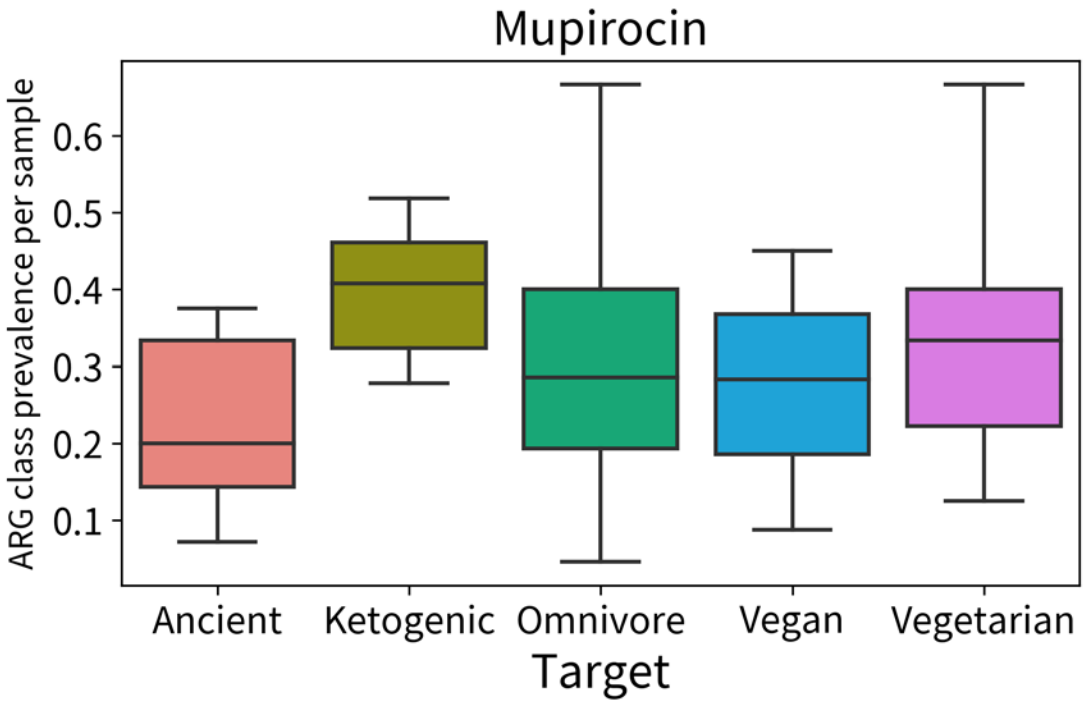

FIG S1k.

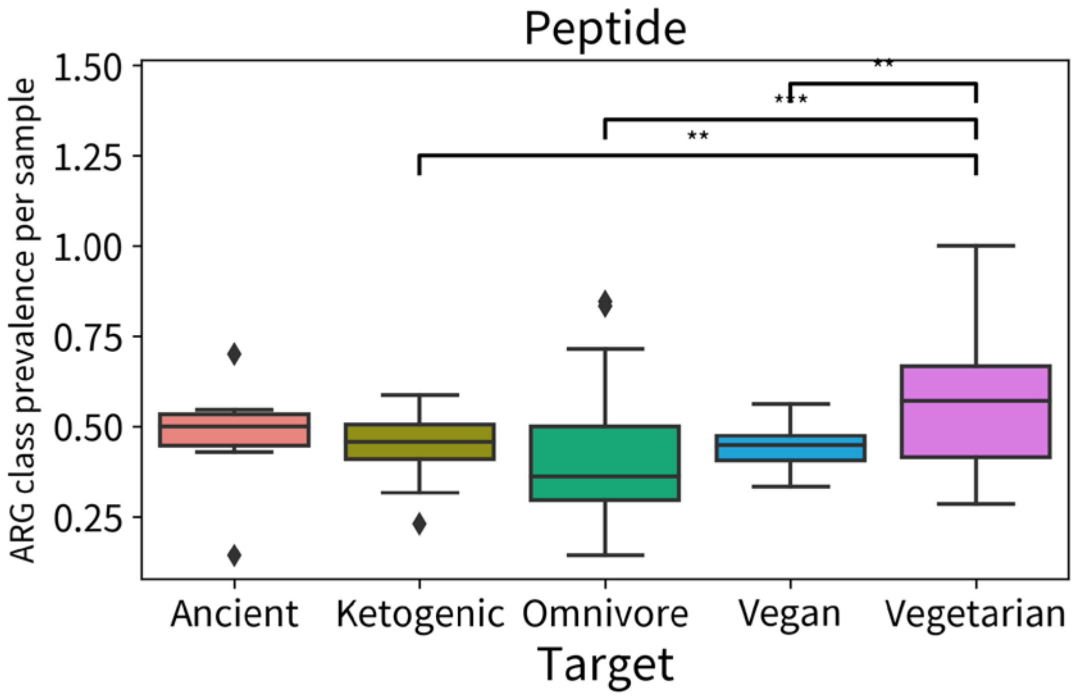

FIG S1l.

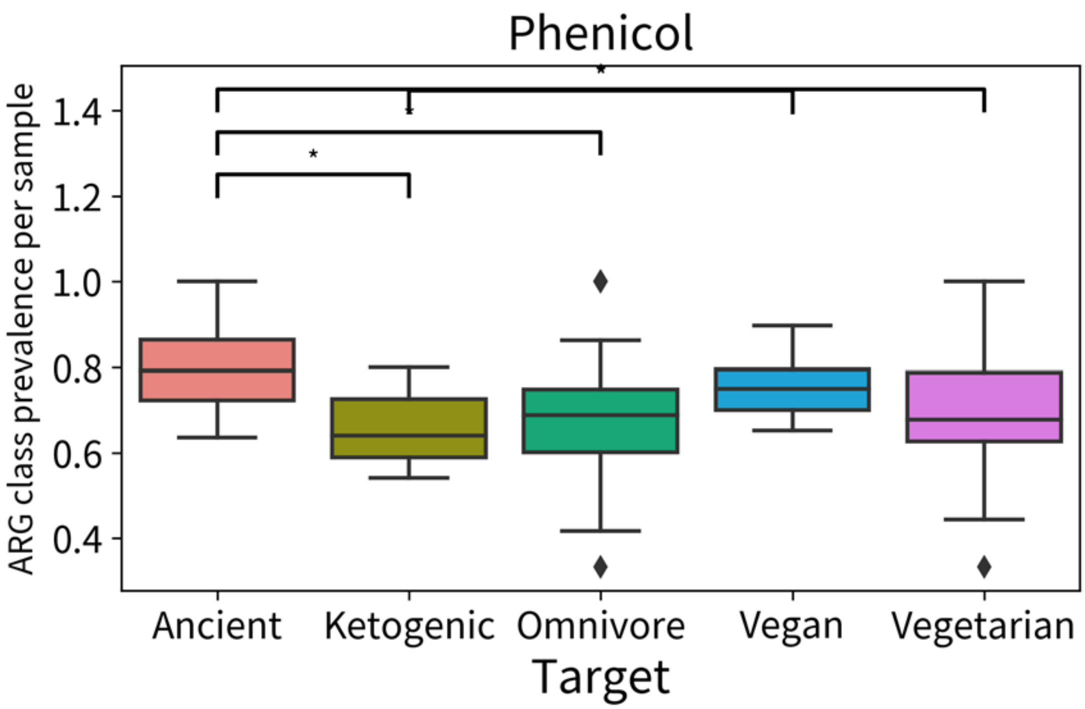
Phenicol
ARG class prevalence per sample
1.4
1.2
1.0
0.8
0.6
0.4
Ancient
Ketogenic
Omnivore
Vegan
Vegetarian
Target
*
*
*

FIG S1m.

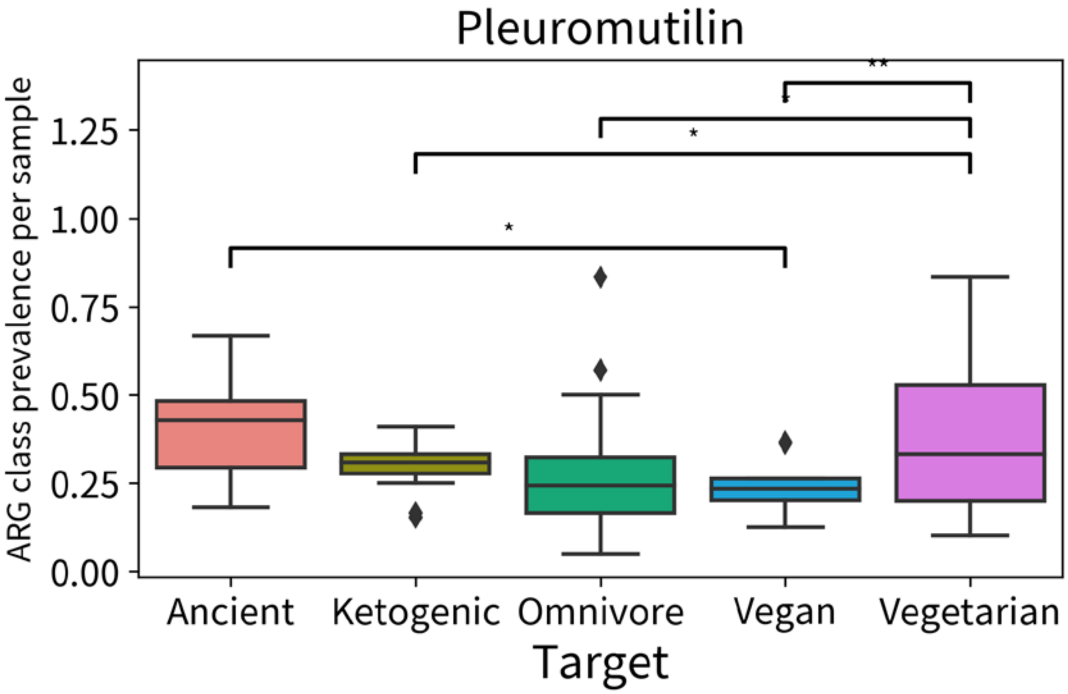

FIG S1n.

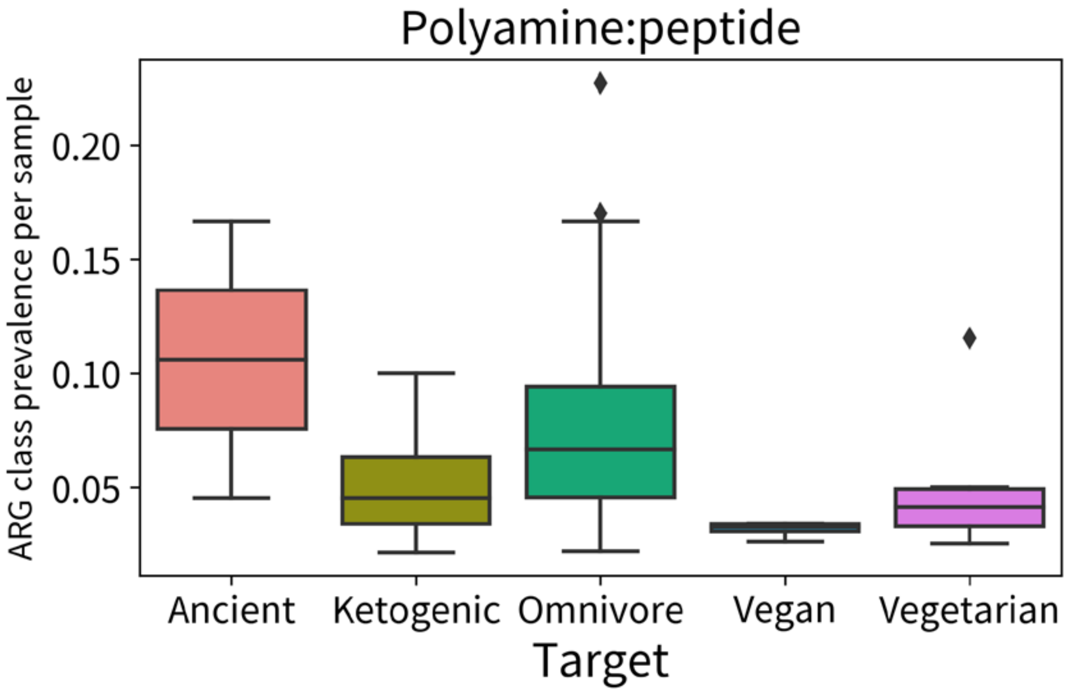

FIG S1o.

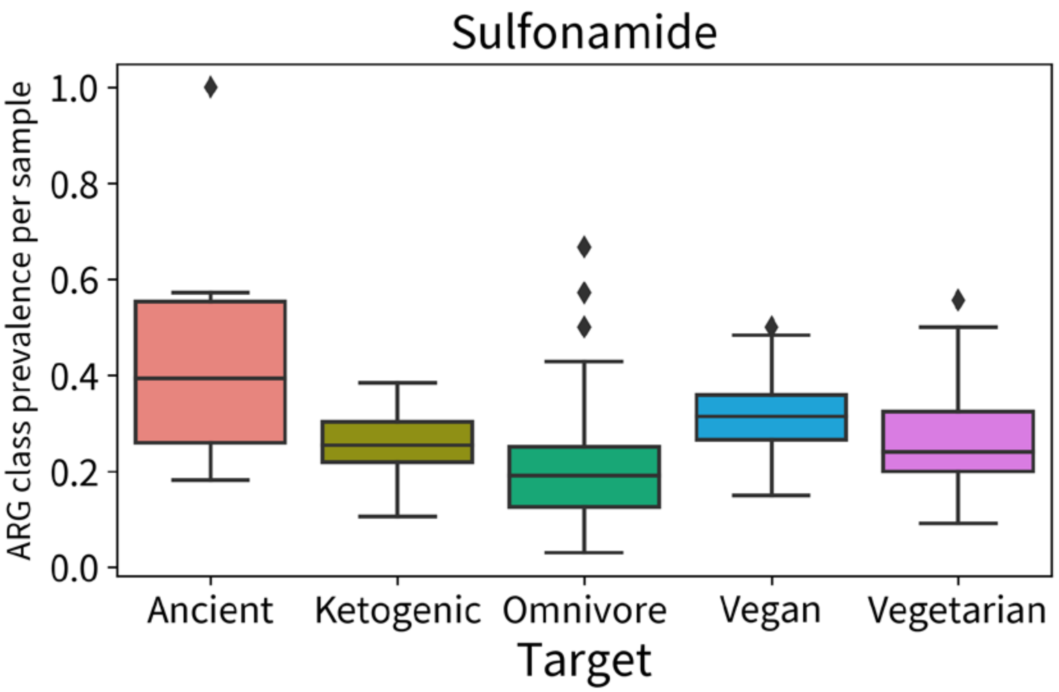

FIG S1p.

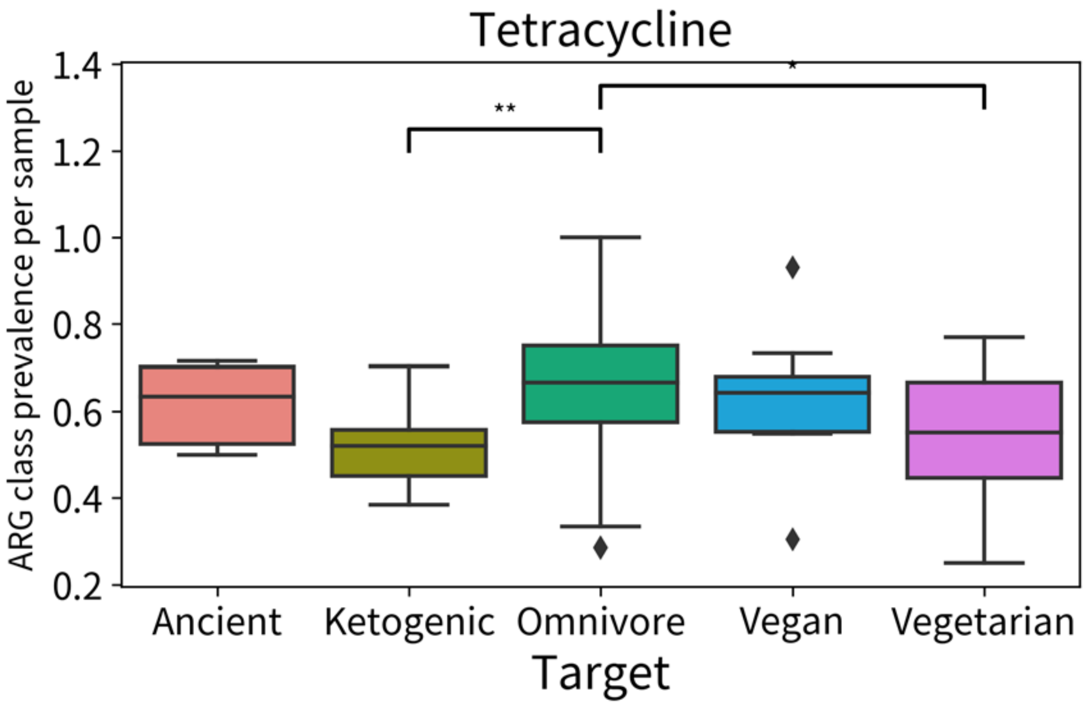

FIG S1q.

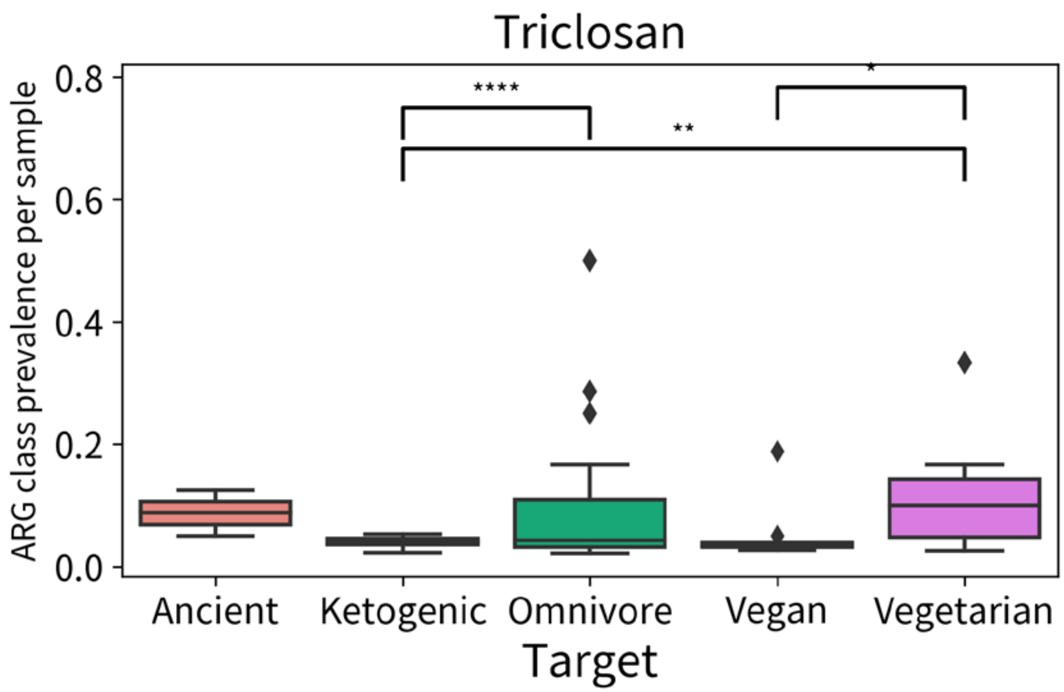

FIG S1r.

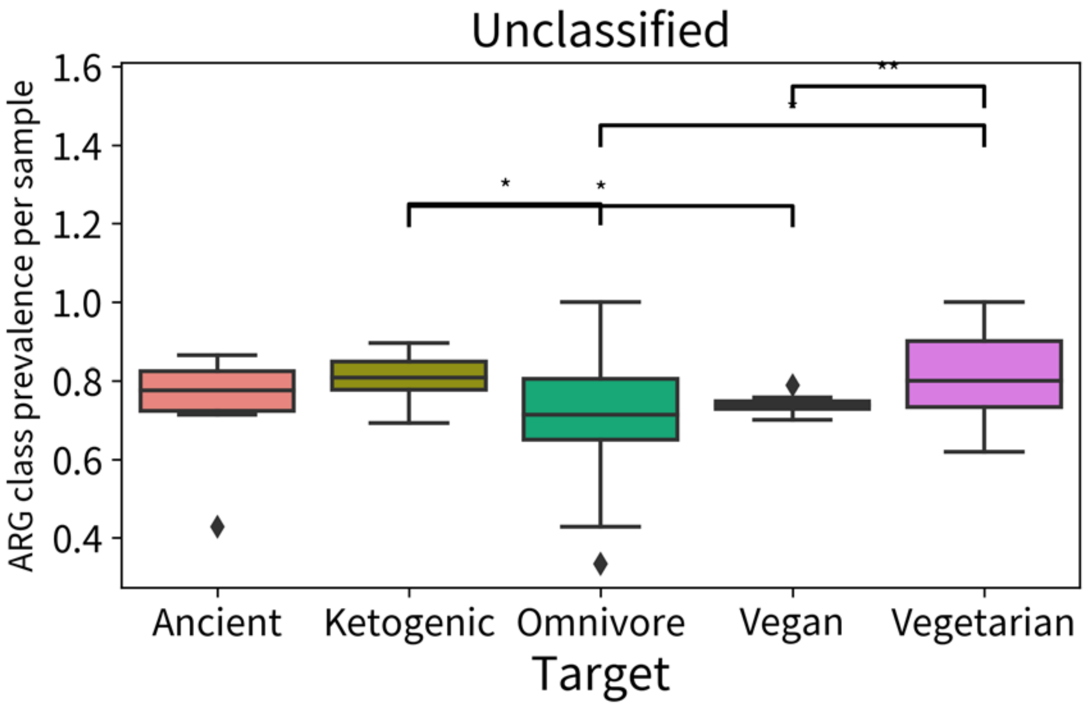

Supplement: giaf072_Supplemental_Files [file giaf072_supplemental_files.zip › 04_Kasmanas_gSpread_AddFile4_Fig_S1.pdf]
