# Supplementary material for: Integrating comparative genomics and risk classification by assessing virulence, antimicrobial resistance, and plasmid spread in microbial communities with gSpreadComp
Source: Gigascience. 2025 Jun 26;14:giaf072. doi: 10.1093/gigascience/giaf072 (PMC12199706; doi:10.1093/gigascience/giaf072)
Supplement: giaf072_Supplemental_Files [file giaf072_supplemental_files.zip › 06_Kasmanas_gSpread_AddFile6_Fig_S2.pdf]

Streamlining microbial community analysis for potential resistance, virulence, and plasmid-mediated spread through integrated comparative genomics and relative risk ranking using gSpreadComp

Jonas Coelho Kasmanas [a,b,c], Stefanía Magnúsdóttir [a], Junya Zhang [d], Kornelia Smalla [e], Michael Schloter[f], Peter F. Stadler [c], André Carlos Ponce de Leon Ferreira de Carvalho [b], Ulisses Rocha [a]#

[a] Department of Environmental Microbiology, Helmholtz Centre for Environmental Research – UFZ, Leipzig, Germany.

[b] Institute of Mathematics and Computer Sciences, University of São Paulo, São Carlos, Brazil.

[c] Department of Computer Science and Interdisciplinary Center of Bioinformatics, University of Leipzig, Leipzig, Germany.

[d] Department of Isotope Biogeochemistry, Helmholtz Centre for Environmental Research – UFZ, Leipzig, Germany.

[e] Julius Kühn-Institut, Federal Research Centre for Cultivated Plants, Institute for Epidemiology and Pathogen Diagnostics, Braunschweig, Germany

[f] Helmholtz Center Munich, National Research Center for Environmental Health, Insitute for Comparative Microbiome Analysis, Neuherberg, Germany

#Address correspondence to Ulisses Rocha, ulisses.rocha@ufz.de

FIG S2. Heatmaps containing the spread, calculated as weighted average prevalence (WAP) of the antimicrobial resistance genes (ARG) classes (rows) per phyla (columns) per target diet (title). The number between paratheses after the phyla indicates the number of genomes used for the calculation from that phylum. The number between parentheses from the ARG classes is the average spread for that ARG class.

FIG S2a.

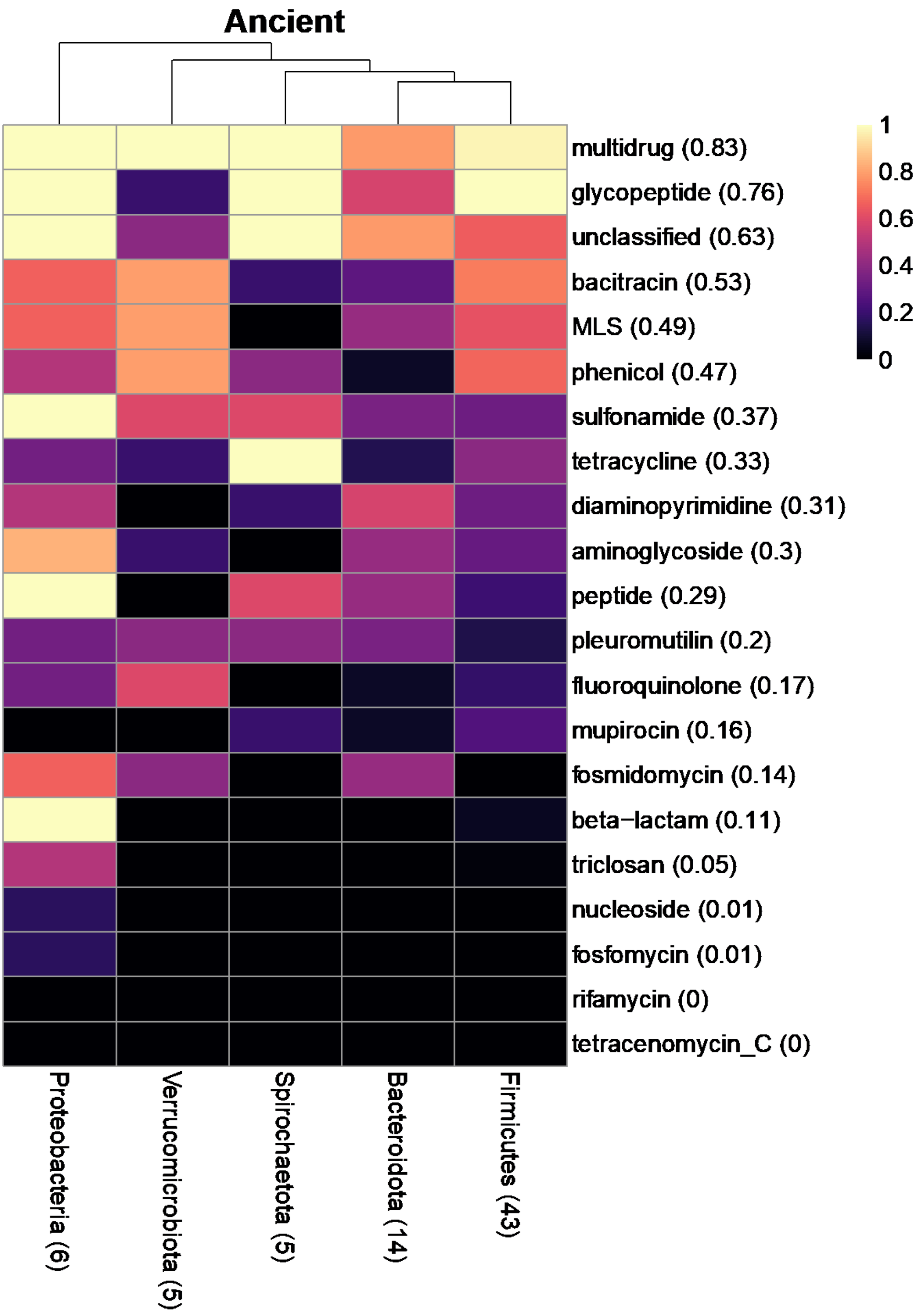
Ancient
multidrug (0.83)
glycopeptide (0.76)
unclassified (0.63)
bacitracin (0.53)
MLS (0.49)
phenicol (0.47)
sulfonamide (0.37)
tetracycline (0.33)
diaminopyrimidine (0.31)
aminoglycoside (0.3)
peptide (0.29)
pleuromutilin (0.2)
fluoroquinolone (0.17)
mupirocin (0.16)
fosmidomycin (0.14)
beta−lactam (0.11)
triclosan (0.05)
nucleoside (0.01)
fosfomycin (0.01)
rifamycin (0)
tetracenomycin_C (0)
1
0.8
0.6
0.4
0.2
0
Proteobacteria (6)
Verrucomicrobiota (5)
Spirochaetota (5)
Bacteroidota (14)
Firmicutes (43)

FIG S2b

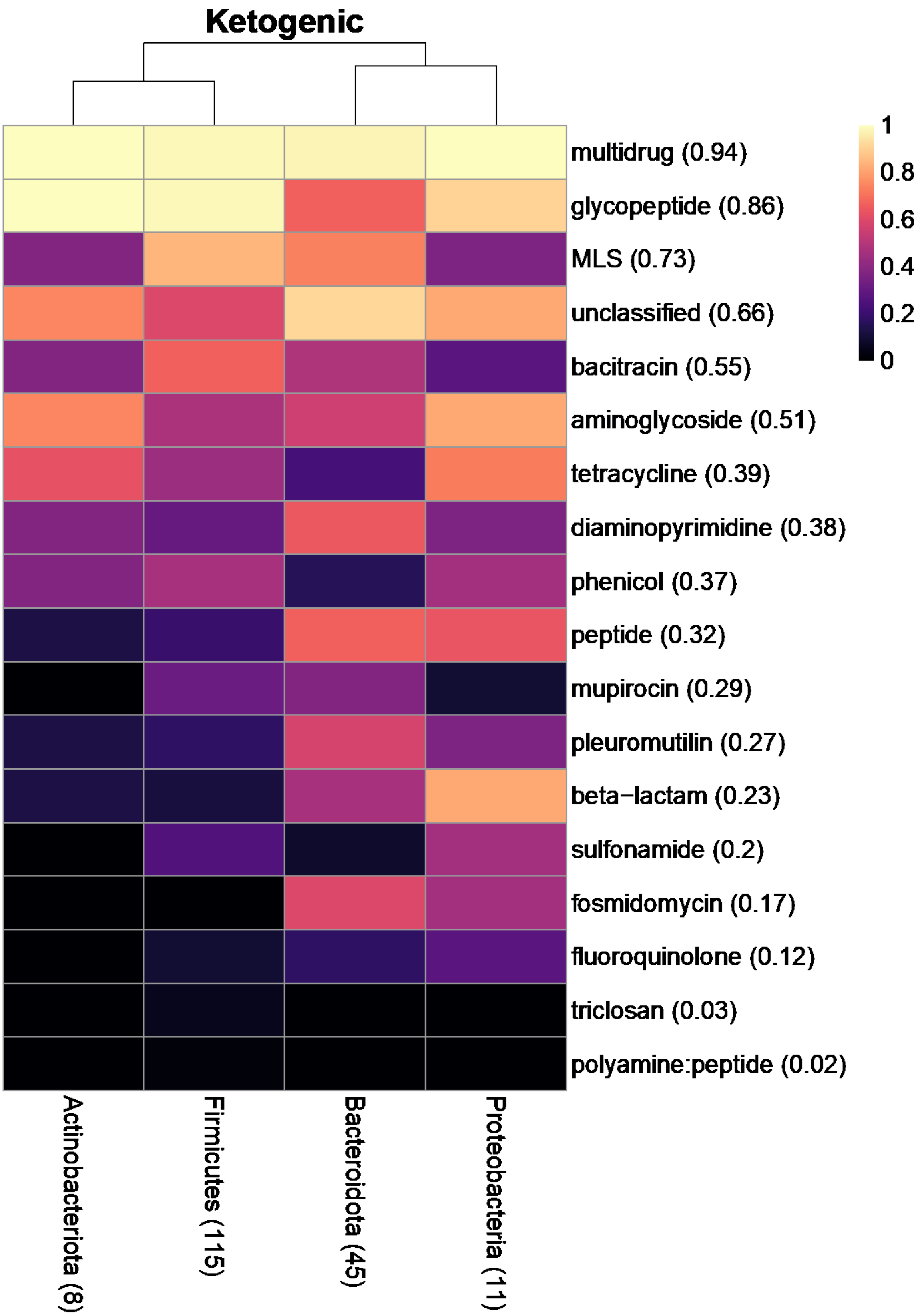
Ketogenic
multidrug (0.94)
glycopeptide (0.86)
MLS (0.73)
unclassified (0.66)
bacitracin (0.55)
aminoglycoside (0.51)
tetracycline (0.39)
diaminopyrimidine (0.38)
phenicol (0.37)
peptide (0.32)
mupirocin (0.29)
pleuromutilin (0.27)
beta−lactam (0.23)
sulfonamide (0.2)
fosmidomycin (0.17)
fluoroquinolone (0.12)
triclosan (0.03)
polyamine:peptide (0.02)
Actinobacteriota (8)
Firmicutes (115)
Bacteroidota (45)
Proteobacteria (11)
1
0.8
0.6
0.4
0.2
0

FIG S2c

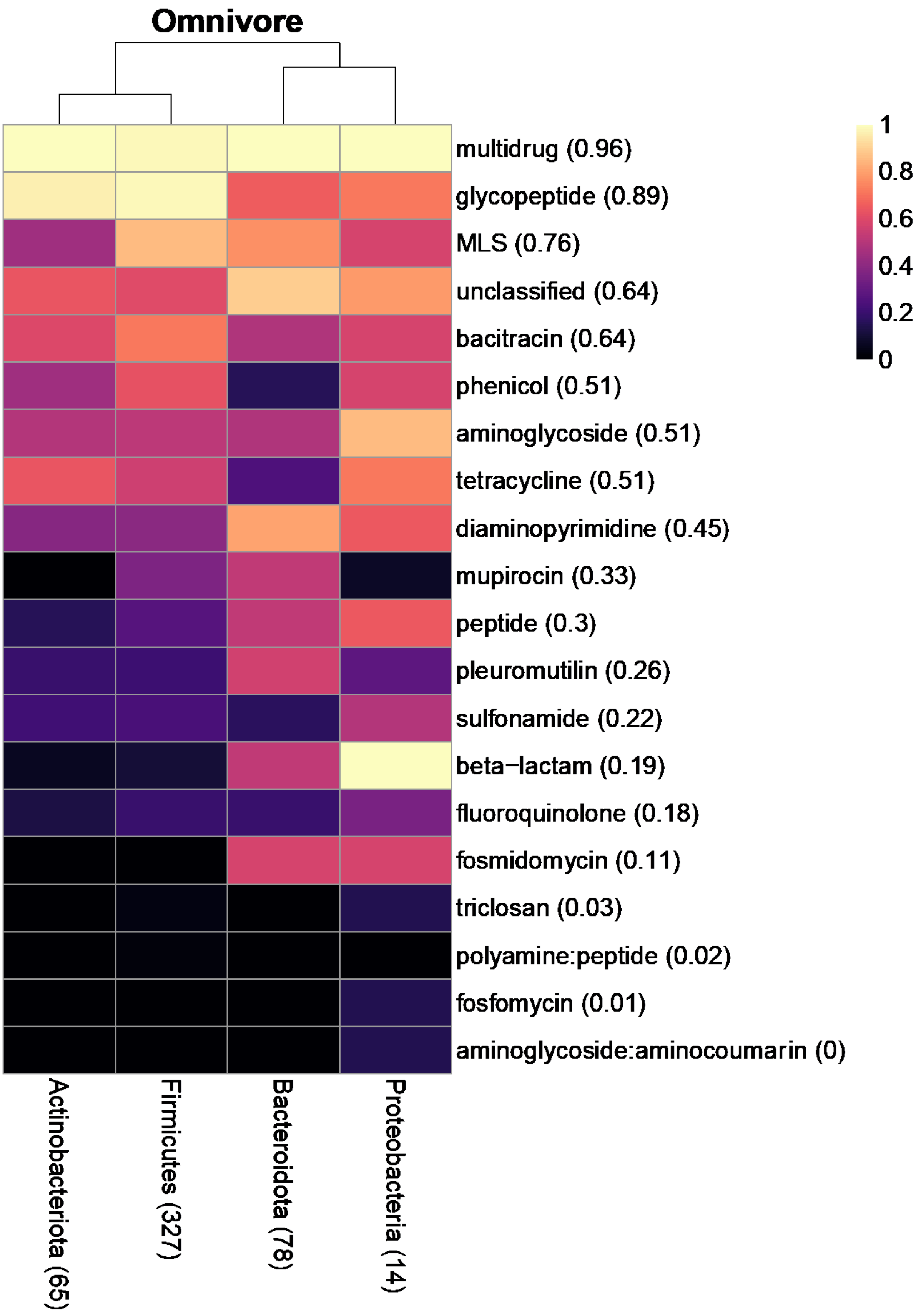

Omnivore
multidrug (0.96)
glycopeptide (0.89)
MLS (0.76)
unclassified (0.64)
bacitracin (0.64)
phenicol (0.51)
aminoglycoside (0.51)
tetracycline (0.51)
diaminopyrimidine (0.45)
mupirocin (0.33)
peptide (0.3)
pleuromutilin (0.26)
sulfonamide (0.22)
beta−lactam (0.19)
fluoroquinolone (0.18)
fosmidomycin (0.11)
triclosan (0.03)
polyamine:peptide (0.02)
fosfomycin (0.01)
aminoglycoside:aminocoumarin (0)
Actinobacteriota (65)
Firmicutes (327)
Bacteroidota (78)
Proteobacteria (14)
1
0.8
0.6
0.4
0.2
0

FIG S2d

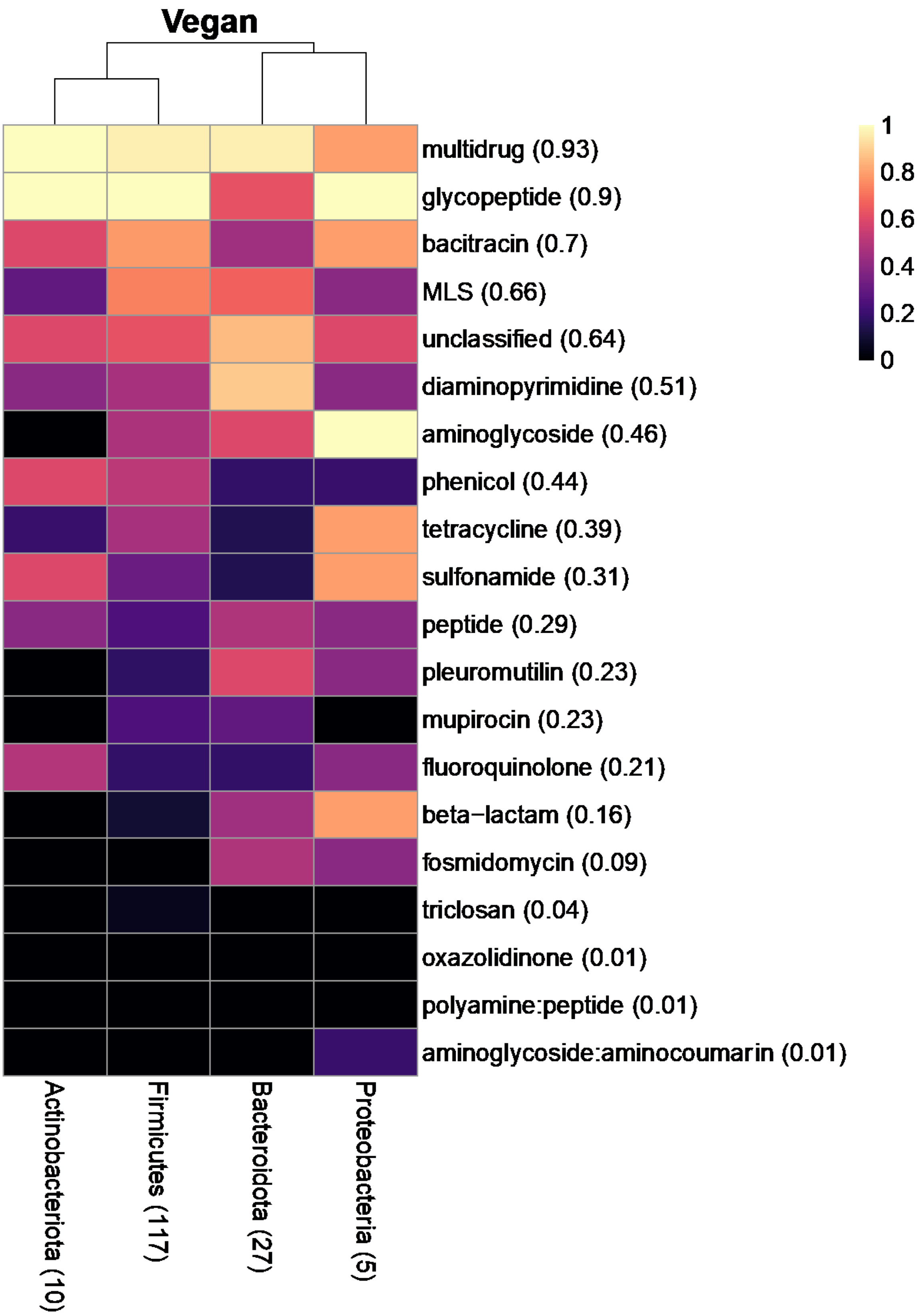

Vegan
multidrug (0.93)
glycopeptide (0.9)
bacitracin (0.7)
MLS (0.66)
unclassified (0.64)
diaminopyrimidine (0.51)
aminoglycoside (0.46)
phenicol (0.44)
tetracycline (0.39)
sulfonamide (0.31)
peptide (0.29)
pleuromutilin (0.23)
mupirocin (0.23)
fluoroquinolone (0.21)
beta−lactam (0.16)
fosmidomycin (0.09)
triclosan (0.04)
oxazolidinone (0.01)
polyamine:peptide (0.01)
aminoglycoside:aminocoumarin (0.01)
Actinobacteriota (10)
Firmicutes (117)
Bacteroidota (27)
Proteobacteria (5)
1
0.8
0.6
0.4
0.2
0

FIG S2e

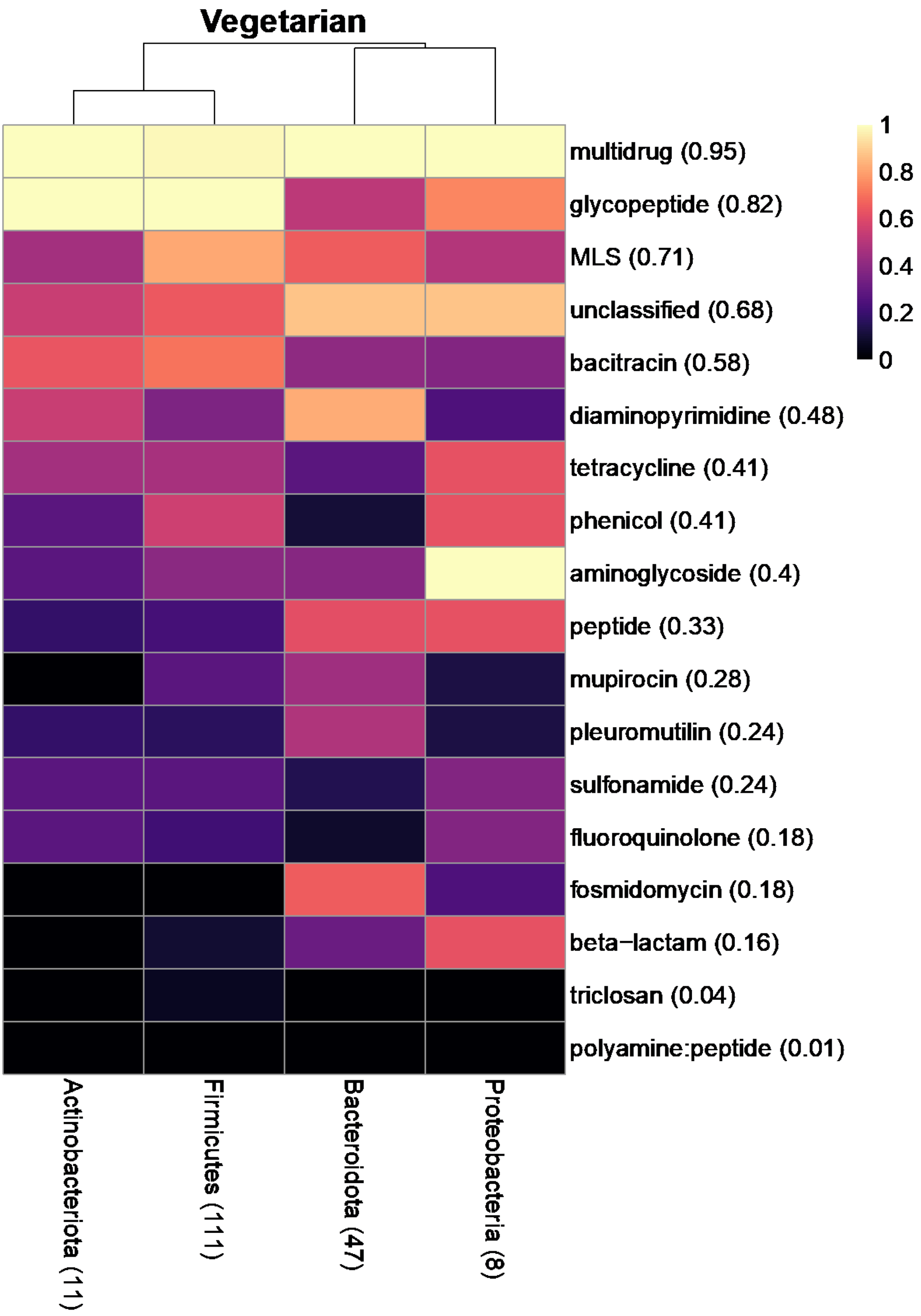
Vegetarian
multidrug (0.95)
glycopeptide (0.82)
MLS (0.71)
unclassified (0.68)
bacitracin (0.58)
diaminopyrimidine (0.48)
tetracycline (0.41)
phenicol (0.41)
aminoglycoside (0.4)
peptide (0.33)
mupirocin (0.28)
pleuromutilin (0.24)
sulfonamide (0.24)
fluoroquinolone (0.18)
fosmidomycin (0.18)
beta−lactam (0.16)
triclosan (0.04)
polyamine:peptide (0.01)
Actinobacteriota (11)
Firmicutes (111)
Bacteroidota (47)
Proteobacteria (8)
1
0.8
0.6
0.4
0.2
0
