# Supplementary material for: Integrating comparative genomics and risk classification by assessing virulence, antimicrobial resistance, and plasmid spread in microbial communities with gSpreadComp
Source: Gigascience. 2025 Jun 26;14:giaf072. doi: 10.1093/gigascience/giaf072 (PMC12199706; doi:10.1093/gigascience/giaf072)
Supplement: giaf072_Supplemental_Files [file giaf072_supplemental_files.zip › 08_Kasmanas_gSpread_AddFile8_Fig_S3.pdf]

Streamlining microbial community analysis for potential resistance, virulence, and plasmid-mediated spread through integrated comparative genomics and relative risk ranking using gSpreadComp

Jonas Coelho Kasmanas [a,b,c], Stefanía Magnúsdóttir [a], Junya Zhang [d], Kornelia Smalla [e], Michael Schloter[f], Peter F. Stadler [c], André Carlos Ponce de Leon Ferreira de Carvalho [b], Ulisses Rocha [a]#

[a] Department of Environmental Microbiology, Helmholtz Centre for Environmental Research – UFZ, Leipzig, Germany.

[b] Institute of Mathematics and Computer Sciences, University of São Paulo, São Carlos, Brazil.

[c] Department of Computer Science and Interdisciplinary Center of Bioinformatics, University of Leipzig, Leipzig, Germany.

[d] Department of Isotope Biogeochemistry, Helmholtz Centre for Environmental Research – UFZ, Leipzig, Germany.

[e] Julius Kühn-Institut, Federal Research Centre for Cultivated Plants, Institute for Epidemiology and Pathogen Diagnostics, Braunschweig, Germany

[f] Helmholtz Center Munich, National Research Center for Environmental Health, Insitute for Comparative Microbiome Analysis, Neuherberg, Germany

#Address correspondence to Ulisses Rocha, ulisses.rocha@ufz.de

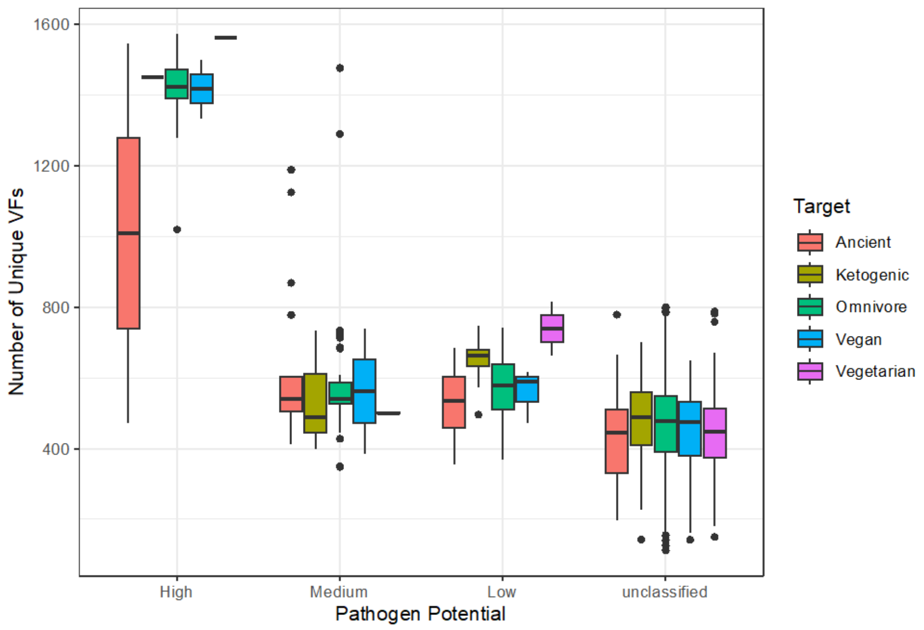

Fig S3a. Boxplots colored by Target diet. The x-axis is grouped by pathogenic potential defined by the taxonomical distance to potential pathogens from the NCBI pathogen database. The y-axis is the number of unique Virulence Factors (VF) per sample.

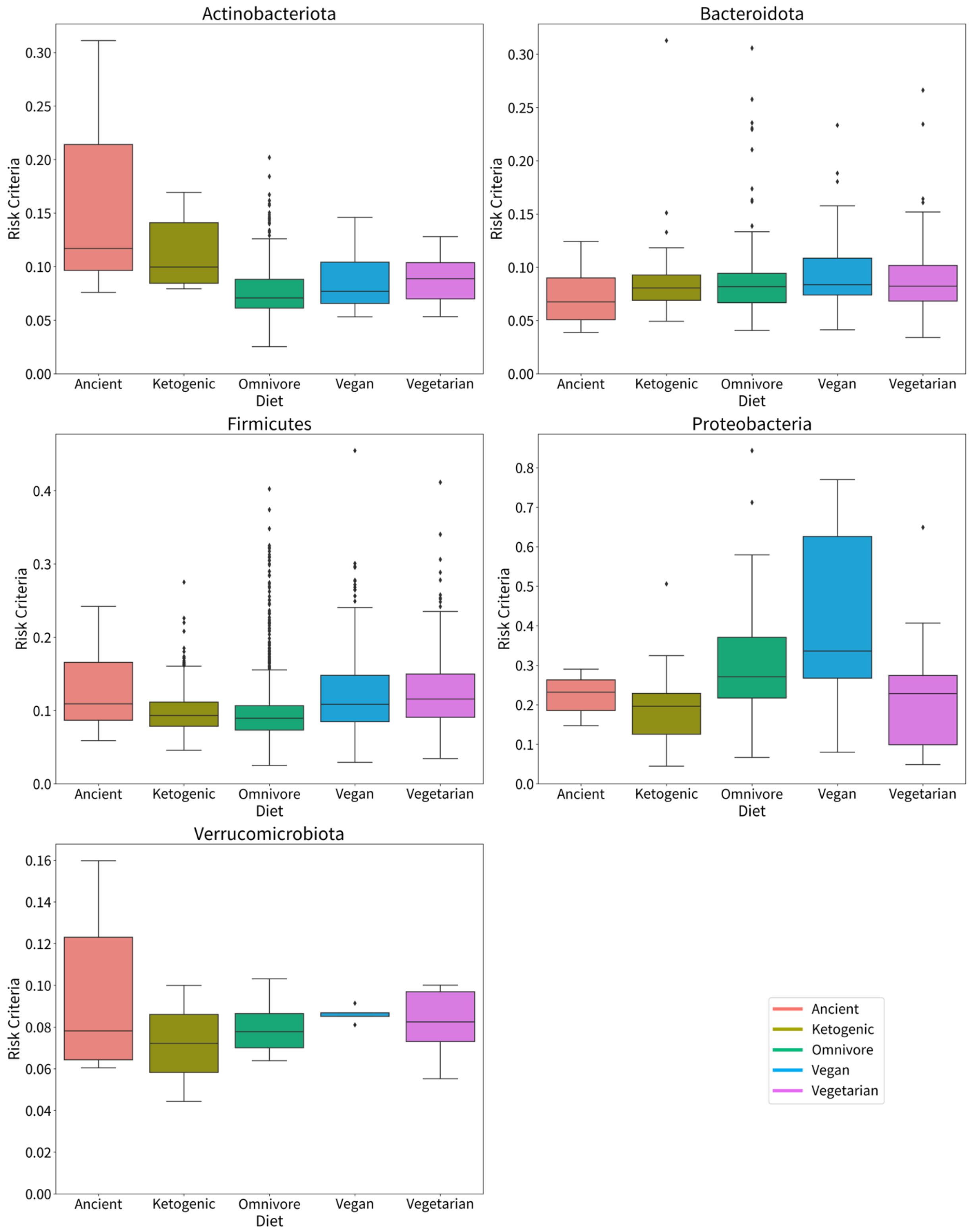

Fig S3b. Group of boxplots per Phylum that are common to all target diets. The x-axis is grouped and colored by target diet. The y-axis has the calculated resistance-virulence risk metric.

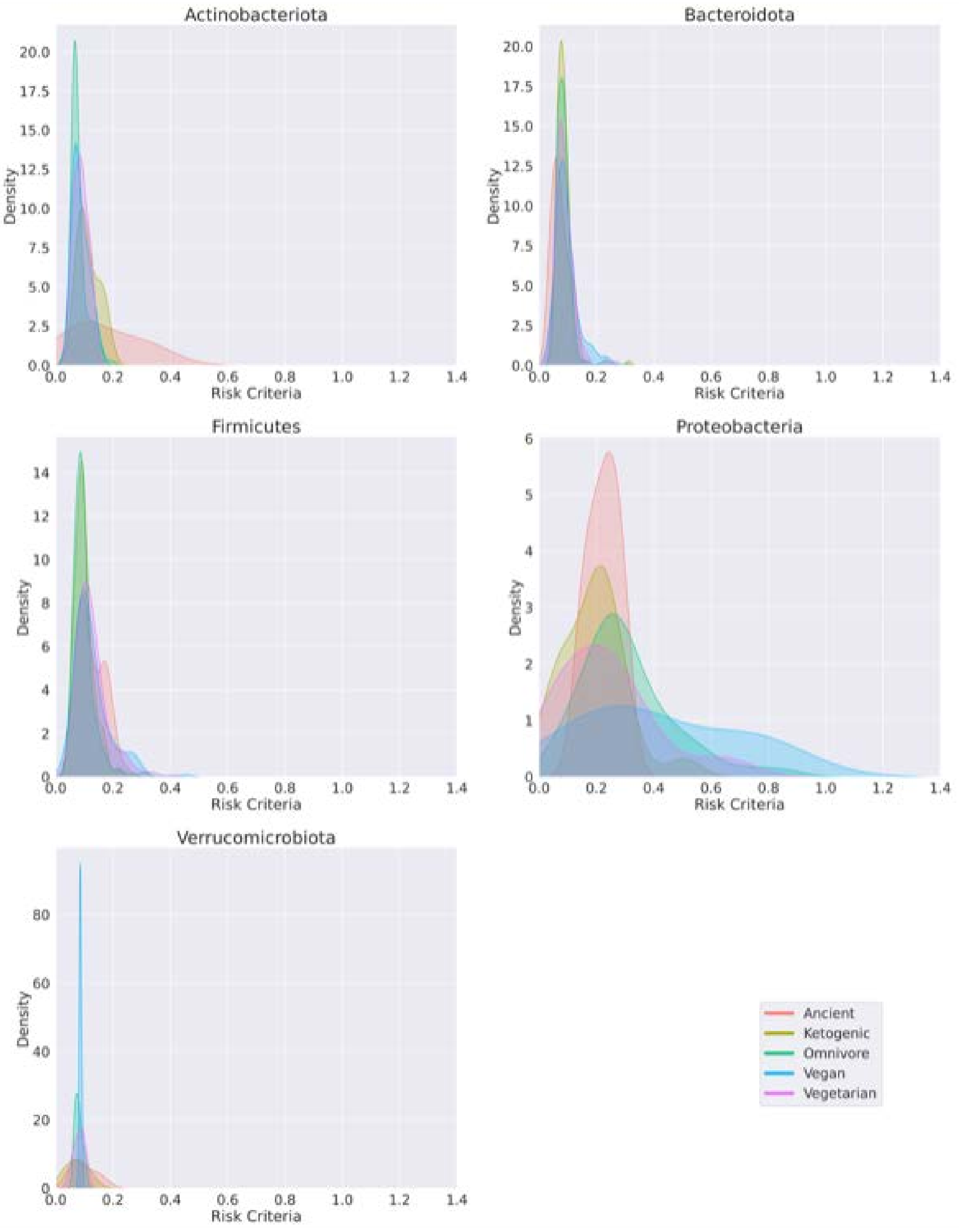

Fig S3c. Density plots of the resistance-virulence risk for each common Phylum colored by target diet. The y-axis indicates the estimated probability density of the respective resistance-virulence risk in the x-axis. Density plots are calculated using the seaborn.kdeplot in Python 3.9.

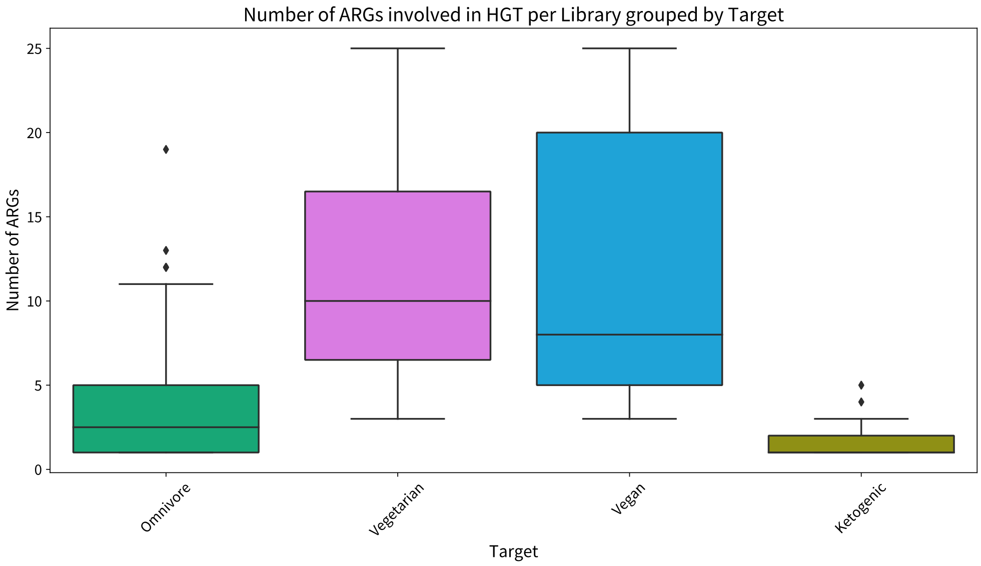

Fig S3d. Boxplot for the number of antimicrobial resistance genes (ARGs) involved in plasmid-mediated horizontal gene transfer (HGT) events found per sample on the y-axis. The x-axis is grouped and colored by target diet.

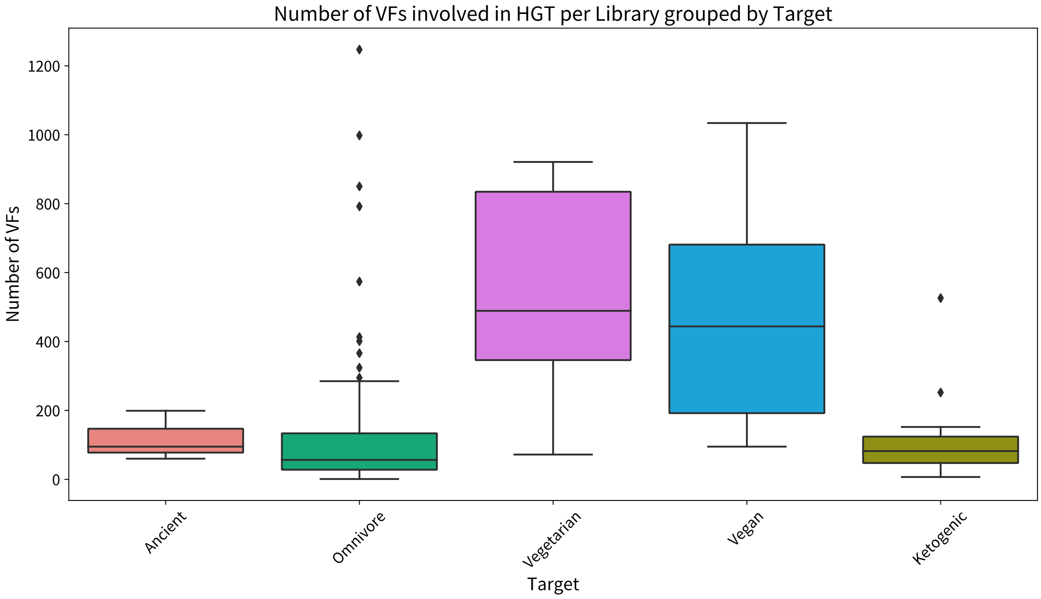

Fig S3e. Boxplot for the number of Virulence Factors (VFs) involved in plasmid-mediated horizontal gene transfer (HGT) events found per sample on the y-axis. The x-axis is grouped and colored by target diet.
