# Supplementary material for: Integrating comparative genomics and risk classification by assessing virulence, antimicrobial resistance, and plasmid spread in microbial communities with gSpreadComp
Source: Gigascience. 2025 Jun 26;14:giaf072. doi: 10.1093/gigascience/giaf072 (PMC12199706; doi:10.1093/gigascience/giaf072)
Supplement: giaf072_GIGA-D-24-00460_original_submission [file giaf072_giga-d-24-00460_original_submission.pdf]

GigaScience

Streamlining microbial community analysis for potential resistance, virulence, and plasmid-mediated spread through integrated comparative genomics and relative risk ranking using gSpreadComp

--Manuscript Draft--

**Manuscript Number:**	GIGA-D-24-00460
**Full Title:**	Streamlining microbial community analysis for potential resistance, virulence, and plasmid-mediated spread through integrated comparative genomics and relative risk ranking using gSpreadComp
**Article Type:**	Technical Note
**Funding Information:**	FAPESP (2019/03396-9) — Mr. Jonas Coelho Kasmanas<br>FAPESP (2022/03534-5) — Mr. Jonas Coelho Kasmanas<br>Helmholtz Association (VH-NG-1248 Micro' Big Data') — Dr. Ulisses Rocha<br>Deutsches Forschungsgemeinschaft (460129525) — Dr. Ulisses Rocha<br>Canada's International Development Research Centre (109981) — Prof. André Carlos Ponce de Leon Ferreira de Carvalho
**Abstract:**	Background<br><br>Comparative genomics, genetic spread analysis, and context-aware ranking are crucial in understanding microbial dynamics' impact on public health. gSpreadComp streamlines the path from in silico analysis to hypothesis generation. By integrating comparative genomics, genome annotation, normalization, plasmid-mediated gene transfer, and microbial resistance-virulence risk ranking into a unified workflow, gSpreadComp facilitates hypothesis generation from complex microbial datasets.<br><br>Findings<br><br>The gSpreadComp workflow works through six modular steps: taxonomy assignment, genome quality estimation, antimicrobial resistance (AMR) gene annotation, plasmid/chromosome classification, virulence factor annotation, and downstream analysis. Our workflow calculates gene spread using normalized weighted average prevalence and ranks potential resistance-virulence risk by integrating microbial resistance, virulence, and plasmid transmissibility data, and producing an HTML report. As a use case, we analyzed 3,566 metagenome-assembled genomes recovered from human gut microbiomes across diets. Our findings indicated consistent AMR across diets, with diet-specific resistance patterns, such as increased bacitracin in Vegans and tetracycline in Omnivores. Notably, Ketogenic diets showed a slightly higher resistance-virulence rank, while Vegan and Vegetarian encompassed more plasmid-mediated gene transfer.<br><br>Conclusion<br><br>The gSpreadComp workflow aims to facilitate hypothesis generation for targeted experimental validations by the identification of concerning resistant hotspots in complex microbial datasets. Our study raises attention to a more thorough study of the critical role of diet in microbial community dynamics and the spread of AMR. This research underscores the importance of integrating genomic data into public health strategies to combat AMR. The gSpreadComp workflow is available at https://gitfront.io/r/JonasK/VCX9CmPdTvk6/gSpreadComp/).
**Corresponding Author:**	Ulisses Rocha<br>Helmholtz-Centre for Environmental Research - UFZ: Helmholtz-Zentrum fur Umweltforschung UFZ<br>Leipzig, GERMANY

Powered by Editorial Manager® and ProduXion Manager® from Aries Systems Corporation

Corresponding Author Secondary Information:	
Corresponding Author's Institution:	Helmholtz-Centre for Environmental Research - UFZ: Helmholtz-Zentrum fur Umweltforschung UFZ
Corresponding Author's Secondary Institution:	
First Author:	Jonas Coelho Kasmanas
First Author Secondary Information:	
Order of Authors:	Jonas Coelho Kasmanas
	Stefanía Magnúsdóttir
	Junya Zhang
	Kornelia Smalla
	Michael Schloter
	Peter F. Stadler
	André Carlos Ponce de Leon Ferreira de Carvalho
	Ulisses Rocha
Order of Authors Secondary Information:	
Additional Information:	
**Question**	**Response**
Are you submitting this manuscript to a special series or article collection?	No
**Experimental design and statistics**<br><br>Full details of the experimental design and statistical methods used should be given in the Methods section, as detailed in our Minimum Standards Reporting Checklist. Information essential to interpreting the data presented should be made available in the figure legends.<br><br>Have you included all the information requested in your manuscript?	Yes
**Resources**<br><br>A description of all resources used, including antibodies, cell lines, animals and software tools, with enough information to allow them to be uniquely identified, should be included in the Methods section. Authors are strongly encouraged to cite Research Resource Identifiers (RRIDs) for antibodies, model	Yes

organisms and tools, where possible.<br><br>Have you included the information requested as detailed in our Minimum Standards Reporting Checklist?	
**Availability of data and materials**<br><br>All datasets and code on which the conclusions of the paper rely must be either included in your submission or deposited in publicly available repositories (where available and ethically appropriate), referencing such data using a unique identifier in the references and in the “Availability of Data and Materials” section of your manuscript.<br><br>Have you have met the above requirement as detailed in our Minimum Standards Reporting Checklist?	Yes
GigaScience has policies and guidelines in place for the use of generative AI-writing tools such as ChatGPT. If you have used such writing tools to assist with writing the manuscript this must be declared and cited in the text. Authors should not list AI-writing tools and other AI-assisted technologies as an author or co-author and should acknowledge that they are fully responsible for text generated or refined by AI-writing tools.<p><br><br>A summary of use (particularly in the introduction or among methods) needs to be included at the end of the paper, and the outputs should also be included as a supplementary file hosted in GigaDB or other open repositories. Please read our guidelines for more information. <p><br><br>By submitting to GigaScience, you are aware of the journal's AI-writing tools policy, and if you have declared use of such tools below, you have acknowledged this where appropriate in your manuscript and have made a summary of use and outputs available. <p><br><b>AI-assisted writing tools have been	No

used in the preparation of this manuscript?	

Powered by Editorial Manager® and ProduXion Manager® from Aries Systems Corporation

Manuscript unmarked
Click here to access/download;Manuscript;00_Kasmanas_gSpread_manusc

Streamlining microbial community analysis for potential resistance, virulence, and plasmid-mediated spread through integrated comparative genomics and relative risk ranking using gSpreadComp

Jonas Coelho Kasmanas[a,b,c], Stefanía Magnúsdóttir[a], Junya Zhang[d], Kornelia Smalla[e], Michael Schloter[f], Peter F. Stadler[c], André Carlos Ponce de Leon Ferreira de Carvalho[b], Ulisses Rocha[a]#

[a] Department of Applied and Environmental Microbiology, Helmholtz Centre for Environmental Research – UFZ, Leipzig, Germany.

[b] Institute of Mathematics and Computer Sciences, University of São Paulo, São Carlos, Brazil.

[c] Department of Computer Science and Interdisciplinary Center of Bioinformatics, University of Leipzig, Leipzig, Germany.

[d] Department of Isotope Biogeochemistry, Helmholtz Centre for Environmental Research – UFZ, Leipzig, Germany.

[e] Julius Kühn-Institut, Federal Research Centre for Cultivated Plants, Institute for Epidemiology and Pathogen Diagnostics, Braunschweig, Germany

[f] Helmholtz Center Munich, National Research Center for Environmental Health, Insitute for Comparative Microbiome Analysis, Neuherberg, Germany

#Address correspondence to Ulisses Rocha, ulisses.rocha@ufz.de

Abstract

Background

Comparative genomics, genetic spread analysis, and context-aware ranking are crucial in understanding microbial dynamics' impact on public health. gSpreadComp streamlines the path from in silico analysis to hypothesis generation. By integrating comparative genomics, genome annotation, normalization, plasmid-mediated gene transfer, and microbial resistance-virulence risk ranking into a unified workflow, gSpreadComp facilitates hypothesis generation from complex microbial datasets.

Findings

The gSpreadComp workflow works through six modular steps: taxonomy assignment, genome quality estimation, antimicrobial resistance (AMR) gene annotation, plasmid/chromosome classification, virulence factor annotation, and downstream analysis. Our workflow calculates gene spread using normalized weighted average prevalence and ranks potential resistance-virulence risk by integrating microbial resistance, virulence, and plasmid transmissibility data, and producing an HTML report. As a use case, we analyzed 3,566 metagenome-assembled genomes recovered from human gut microbiomes across diets. Our findings indicated consistent AMR across diets, with diet-specific resistance patterns, such as increased bacitracin in Vegans and tetracycline in Omnivores. Notably, Ketogenic diets showed a slightly higher resistance-virulence rank, while Vegan and Vegetarian encompassed more plasmid-mediated gene transfer.

Conclusion

The gSpreadComp workflow aims to facilitate hypothesis generation for targeted experimental validations by the identification of concerning resistant hotspots in complex microbial datasets. Our study raises attention to a more thorough study of the critical role of diet in microbial community dynamics and the spread of AMR. This research underscores the importance of integrating genomic data into public health strategies to combat AMR. The gSpreadComp workflow is available at https://gitfront.io/r/JonasK/VCX9CmPdTvk6/gSpreadComp/).

KEYWORDS Risk ranking, Comparative Genomics, Gene Spread, Human Microbiome, Virulence Factors, Horizontal Transmission, Metagenome-assembled Genomes, Antimicrobial Resistance.

Background

The microbial safety of food, water, and environmental matrices has been a critical concern for public health since the 1990s [1]. Different approaches, such as quantitative microbial risk assessment, have provided valuable insights and have been fundamental in evidence-based policy-making in public health. Typically, these approaches involve four steps: hazard identification, exposure assessment, dose-response analysis, and risk characterization [2]. However, traditional microbial safety approaches often focus on individual potential pathogens and may overlook community interactions.

Additionally, the advent of high-throughput sequencing technologies has improved our ability to study microbial communities with increased detail. Advances in sequencing technologies can potentially enhance our understanding of microbial ecology and improve microbial analysis's accuracy, precision, and speed [3]. Concomitantly to the advances in understanding microbial ecology, there is a growing need for community-focused approaches to assess relative impacts across diverse microbial populations. When integrated with exposure and dose-response data, such an approach would equip decision-makers and stakeholders with a more robust risk statement. Specifically, identifying antimicrobial resistance (AMR) spread, virulence factor (VF) spread, and genetic mobility factors are crucial for an enhanced microbial risk characterization [3, 4].

Essentially, genetic information is spread among entities by vertical gene transfer (VGT) and horizontal gene transfer (HGT). While VGT is relevant for preserving and stabilizing genetic material, HGT has a crucial role in the evolutionary and adaptive process [5]. Consequently, HGT allows microbes in microbial communities to perform functional leaps and rapidly adapt to new environments. There are three most recognized mechanisms of HGT in prokaryotes: conjugation, transformation, and transduction. Conjugation requires physical contact between the cells. Transformation is the uptake of exogenous DNA, mostly plasmids, from the environment. Transduction is the delivery of genetic material through viruses and virus-like agents[6]. However, even though transduction and transformation events are effective for gene exchange, plasmid-mediated conjugation is often

recognized as the most impactful HGT mechanism [7]. Plasmids often carry genes that allow potential selective advantages, e.g., AMR or heavy metal resistance, VFs, and degradation of xenobiotics [8, 9].

Specifically, the spread of AMR in clinical and natural environments is recognized as one of the most significant global threats [10, 11]. The misuse of antibiotics in agriculture, the environment, and human medicine creates selective pressure on antimicrobial-resistant bacteria (ARB), which may facilitate the HGT of those resistances. Antibiotics are extensively used for farm animals' and plant production [12, 13]. In 2015, a notable trend emerged in the USA, where 62% of antibiotics initially intended for use in food-producing animals were ultimately utilized in human medicine. Additionally, 70% of medically relevant antibiotics were sold for animal use. [14]. Furthermore, while the use of antibiotics in plant agriculture is generally considered lower than in human and veterinary medicine, recent studies suggest it may be more widespread than previously thought. Streptomycin, oxytetracycline, kasugamycin, oxolinic acid, and gentamicin are commonly used in crop protection, particularly in the American and Asian continents [15].

In addition, HGT events provide rapid adaptation to bacteria strains, including AMR, making the development of novel antimicrobials only a short-term palliative measure [16]. Minimizing problematic HGT and the dissemination of antimicrobial resistance genes (ARGs) is the potential long-term solution to the AMR problem. Inherently, advances in understanding plasmid-mediated HGT dynamics in complex microbiomes are a powerful tool to control horizontal dissemination [17, 18].

Although HGT events, specifically plasmid-mediated transfers, play a significant role in the evolution and adaptation of microbial populations, most of those events remain undetected. Consequently, several bioinformatics tools and algorithms were developed to tackle HGT events. For instance, GIST [19] and IslandViewer [20] use genome sequences' features to assign HGT. DarkHorse [21] and HGTector [22] use the "best matches" approach to identify HGT events based on reference genomes. Other methods, such as Ranger-DTL [23] and AnGST [24], require the reconciliation of gene trees with the corresponding species trees to make the HGT prediction. Finally, the MetaCHIP [25] tool combines the results of the similarity and phylogenetic approaches.

A significant limitation of most current HGT detection methods is that they are not directly applicable to the entire microbiome but more for single bacteria taxa. In addition, most methods require reference genomes. For instance, the HGTector [22] is restricted to HGT events from a defined distal group to designated self-group members, while DarkHorse [21] requires a reference genome, a bottleneck for uncultured microorganisms. MetaCHIP [25] can be applied at the community level, given a set of recovered genomes. However, MetaCHIP [25] does not directly integrate its results into relevant sample metadata (i.e., biome, clinical data, environmental condition), reducing its usage for comparative genomics. In addition, none of the mentioned tools allows for direct integration of plasmids-mediated transfer of annotated genes to potential pathogenic bacteria by using, e.g., comparative genomics, which creates a significant barrier for non-bioinformaticians, mainly clinicians, to use such data sets. Finally, plasmids have also been reported to be transferred over considerable taxonomic distances, adding complexity for HGT detection tools to identify plasmid-mediated transfer in complex microbial communities [25, 26].

We designed the gSpreadComp workflow to tackle the following bottlenecks: (a) reduce the barrier of comparative genomics by integrating genome annotation, normalization, and sequence comparison into a unified approach; (b) create a systematic approach to quantify gene spread; (c) integrate plasmid-mediated gene transfer annotation to target metadata with the whole microbiome community in a genome-reference independent approach; (d) provide a resistance-virulence risk ranking metric that considers gene spread, prokaryotic resistance potential, and virulence potential in the era of high-throughput microbial community sequencing. Consequently, gSpreadComp is a UNIX-based workflow for genome analysis (Fig. 1) that provides six modules to perform the following tasks: taxonomy assignment, genome quality estimation, ARGs annotation, plasmid/chromosome classification, VFs annotation, and in-depth downstream analysis.

To demonstrate the potential of the gSpreadComp workflow, we analyzed the spread of ARGs in the human gut microbiome from human subjects with different diets. To this end, we gathered publicly available metagenomes from the human gut containing information about the subjects` diet: (i) Ancient,

diet based on the analysis of ancient human fecal remains; (ii) Ketogenic, fecal samples from subjects with high-fat, and protein, low-carbohydrate diet; (iii) Omnivore, fecal samples from subjects with a diverse diet including both plant and animal-derived foods; (iv) Vegan, fecal samples from subjects with a plant-based diet excluding all animal-derived products; (v) Vegetarian, fecal samples from subjects with diet excluding meat but may include other animal-derived products. We then recovered the metagenomes-assembled genomes (MAGs) from those samples and annotated their ARGs and taxonomy. Finally, those MAGs were analyzed using gSpreadComp using the subjects's diet as the target metadata. Notably, the primary objective of this use case is not to draw definitive conclusions about the relationship between diet and antimicrobial resistance or virulence but to exemplify how gSpreadComp can be applied to complex metagenomic datasets.

Our data revealed antimicrobial resistance, particularly to multidrug and glycopeptide classes, to be widespread across all diets, with specific resistances like bacitracin being more prevalent in Vegans. Additionally, while all diets exhibited similar overall resistance spread, nuances like increased tetracycline resistance in Omnivores were observed. The study also highlighted a complex relationship between diet and VFs, with specific diets showing heightened resistance-virulence risks, like Ketogenic. Finally, Vegans and Vegetarians were associated with a higher potential to participate in plasmid-mediated HGT events, underscoring the significant role of diet in shaping microbial communities and antimicrobial resistance patterns. While further laboratory validation is required, gSpreadComp accelerates the identification of potential targets, streamlining the path from in silico analysis to hypothesis validation through experimental verification.

Findings

The gSpreadComp workflow. The gSpreadComp workflow is a UNIX-based integrated set of tools for genome analysis (Fig. 1). For such, it provides six modules to perform the following tasks: taxonomy assignment, genome quality estimation, ARGs annotation, plasmid/chromosome classification, VFs annotation, and in-depth downstream analysis. This downstream analysis includes target-based gene spread analysis, plasmid-mediated HGT of target genes and VFs, and a prokaryotic resistance-virulence

risk ranking within the analyzed genomes. It is important to note that gSpreadComp is essentially modular, allowing for the integration of new advances in its component methods and tools as they become available.

The spread of target genes was calculated using the genes' weighted average prevalence (WAP), which estimates the gene spread at different taxonomical levels or target groups (e.g., Omnivores, Vegans, Ketogenic). More details can be found in the methods section. For resistance-virulence risk ranking, we defined the "Resistance-virulence potential Factors" that consider target genes (ARGs, by default), virulence, and their plasmid transmissibility potential. Reference potential pathogens were identified by comparing genomes to the NCBI pathogens database [27]. Following, we used the average of the resistance-virulence factors from the reference potential pathogens, based on the NCBI Pathogens Organism groups, as weights and quantified the resistance-virulence risk using the Technique for Order Preference by Similarity to Ideal Solution (TOPSIS) [28], with the resistance-virulence factors serving as input vectors. After the complete downstream analysis, gSpreadComp produced an HTML report.

The gSpreadComp workflow includes an easy-to-use script that downloads and configures the required databases automatically. Consequently, if the user is interested in ARG spread, the only mandatory inputs for gSpreadComp are the genomes and their target metadata. Suppose the user is interested in a different target gene group. In that case, they should provide the annotation table formatted as described in the gSpreadComp documentation. A database update is scheduled to happen every January and July.

Part of gSpreadComp is a wrapper of several bioinformatic approaches. Its modular nature makes it possible to use the tools independently, allowing the use of the tools' main analysis and the related report without the need to annotate it within the software completely. Additionally, the modular nature of the software facilitates its update and allows the more experienced user to integrate only pieces of gSpreadComp into their pipeline. Consequently, gSpreadComp modularity can give the researcher flexibility in their analysis and facilitate the investigator's software management necessities. The

gSpreadComp workflow was designed to support Linux x64 systems. The complete software installation requires approximately 15 GB. The whole database currently requires around 92 GB.

Critical Usage and Key Considerations

Before presenting the experimental results, it is crucial to address specific methodological considerations and limitations in the methods. The gSpreadComp workflow can be used with both complete genomes and MAGs. In our use case, we applied gSpreadComp to MAGs, which are prone to higher potential bias [29], e.g., MAGs are subject to detection bias, particularly for low-abundance organisms, which may lead to the underrepresentation of certain species and their associated ARGs. Additionally, even high-quality MAGs (completeness > 90% and contamination < 5) may be exposed to contig binning error, causing contamination [30]. Finally, there are sample size effects. To mitigate the impact of sample size, gSpreadComp employs normalization techniques and weighted average prevalence for spread calculations [31]. Nevertheless, users should note that the resulting resistance-virulence risk ranking is relative to the analyzed community and not an absolute measure across environments.

The ARG annotation module provided within gSpreadComp uses a machine learning-based classification tool, named DeepARG [32]. While DeepARG has demonstrated high accuracy in ARG prediction, its performance can vary according to the antibiotic category and its representation in the training database. For long sequences (DeepARG-LS), the tool achieved precision and recall values equal to 0.99 in the prediction of different categories of ARGs. To minimize false positives, we followed benchmarked recommendations, including using a minimum 80% prediction probability, an e-value alignment lower than 1e-10, and a percent identity of 35% or higher [33]. It is important to note that the hyperparameters (e.g., prediction probability, e-value alignment) can be altered by the user. Users should interpret results with these constraints in mind. Similarly, for plasmid detection, we currently use PlasFlow [34]. While effective, Plasflow has limitations in classifying shorter sequences. We increased the classification threshold parameter (0.7 > threshold) in our analysis to improve precision, while maintaining the high sensitivity, or recall, offered by PlasFlow's models [34, 35]. However, it must be

observed that automatically classifying plasmids remains complex, with significant advances currently in development. Those approaches were selected because of their ability to streamline large-scale annotation and detection while having higher recall, which is of particular importance when dealing with MAGs.

The gSpreadComp workflow was designed to be modular and extendable, allowing a more straightforward incorporation of additional features in future versions, as the field is rapidly evolving. For instance, ARG detection tools like ARG-SHINE [36]or CARD-RGI [37] or plasmid classification tools like PlasClass [35] or PLASMe [38] can be used, and their results are integrated into gSpreadComp downstream analysis, provided that the users format their data according to the gSpreadComp documentation. We encourage users to consider the strengths and limitations of each tool when interpreting results and to validate findings through complementary experimental approaches when possible. It is important to note that gSpreadComp's downstream results rely on the tools' annotations, and results for simulated communities would closely follow their benchmarked performance.

Use Case: gSpreadComp in the human gut microbiome of subjects with different diets.

To show the potential of gSpreadComp to generate hypotheses, we analyzed the spread of ARGs and virulence factors in the human gut microbiome from subjects with different diets. It is important to mention that the primary objective of this use case is not to draw definitive conclusions about the relationship between diet and antimicrobial resistance or virulence, but to illustrate how gSpreadComp can be applied to complex metagenomic datasets to generate insights that could inform more comprehensive risk assessments.

We recovered MAGs of 17 Ketogenic, 10 Vegans, 40 Vegetarians, and 140 Omnivores subjects from the human gut. In addition, we recovered MAGs from 24 palaeofaeces samples dating from 1300 and 5300 years old (Additional file 1: Table S1). We recovered 3566 MAGs (1806 high and 1760 medium quality) from 231 samples (Additional file 2: Table S2). The taxonomic assignment indicated that the MAGs came from 637 species of 12 Phylum (Additional file 2: Table S2a). According to GTDB-

tk, 594 recovered species were assigned to previously recovered genomes, and 43 species groups found are potentially new.

Our analysis included ancient DNA samples, which present unique challenges. Ancient DNA is typically degraded and fragmented, potentially affecting gene annotation accuracy. Moreover, these samples are highly susceptible to contamination from modern sources and post-mortem microbial colonization. While we have taken steps to address these issues, distinguishing endogenous ancient DNA from contaminants remains challenging. These factors do not invalidate our findings but underscore the need for cautious interpretation, especially when comparing ancient and modern microbiomes [39].

We annotated 356 ARG subtypes distributed in 24 different ARG classes (Additional file 3: Table S3a). In the Ancient samples, we annotated 211 unique ARGs belonging to 22 unique ARG classes. In contrast, Ketogenic had 234 and 18, Omnivores had 320 and 22, Vegans had 238 and 21, and Vegetarians 246 and 20, respectively, in their gut microbiome. We also normalized ARG class prevalence per sample (Additional file 3: Table S3b). We kept only the samples that recovered more than six genomes for further prevalence analysis. Fig. 2A shows the normalized prevalence of the ARG classes per sample for all eating habits. In addition, we performed pairwise ARG class prevalence comparisons for all diets (Additional file 3: Table S3c and Additional file 4: Fig. S1). The bacitracin resistance boxplot comparisons can be found in Fig. 2C.

Further, we estimated the ARG class spread at the Phylum level in gut samples of subjects across the different diets (Additional file 5: Table S4a). We defined the following ranges to describe the distribution of ARG classes: Sparse (0 – 0.25), Common (0.25 – 0.5), Widespread (0.5 – 0.75), and Ubiquitous (0.75 – 1). A heatmap with the distribution at Phylum level value per ARG class for all diets can be found in Fig. 2B. Multidrug and glycopeptide resistance were ubiquitous in all subjects, irrespective of the diet. For further analysis, we excluded ARG classes exhibiting a distribution of less than 0.1 across all dietary patterns. The results revealed that among the diets, Omnivores exhibited the highest spread in seven ARG classes: multidrug, MLS (Macrolides, Lincosamides, Streptogramins),

phenicol, aminoglycoside, tetracycline, and mupirocin. In contrast, Vegans demonstrated the highest spread in four ARG classes: glycopeptide, bacitracin, diaminopyrimidine, and fluoroquinolone. For the remaining dietary patterns, the Ketogenic diet had the highest spread in two ARG classes (pleuromutilin and beta-lactam), the Vegetarian diet in two (peptide and fosmidomycin), and the Ancient subjects in one (sulfonamide). However, considering only the ARG classes with at least a 5% difference between all other diets, bacitracin is more spread in Vegans, tetracycline in Omnivore, and sulfonamide in Ancient. When we compared Ketogenic and Omnivore (Meat eaters) against Vegans and Vegetarians (Not meat eaters) according to the mean spread value, we observed that meat eaters had a higher spread for MLS, aminoglycoside, and mupirocin, and non-meat eaters for diaminopyrimidine.

Finally, gSpreadComp also allowed us to individually compare the spread of ARGs among Phylum (Additional file 5: Tables S4b-f and Additional file 6: Fig S2). The results are summarised in Table 1. The subsequent results that gSpreadComp provided were the annotation of VF. (Additional file 7: Table S5a). The average number of unique VFs annotated per diet were: 479.75 ± 116.41 for Ancient, 444.56 ± 88.03 for Ketogenic, 444.54 ± 106.24 for Omnivore, 475.86 ± 163.95 for Vegan, and 438.13 ± 108.40 for Vegetarian. We also verified the average number of unique VFs per Phylum per diet (Additional file 7: Table S5b). Specifically, *Bacteroidota* related to the Ketogenic diet had statistically more unique VFs than all the other diets (Fig. 3C and Additional file 7: Table S5c). Additionally, gSpreadComp calculated all the statistical significance comparisons associated with the unique number of VFs (Additional file 7: Table S5c). We verified, as expected, that MAGs with high pathogenic potential, irrespective of the diet, have a higher number of unique VFs in the gut samples (Additional file 2: Table S2a and Additional file 8: Fig S3a). More interestingly, we observed that irrespective of the diet, highly virulent bacteria were also the ones with statistically more ARGs in the respective gut samples (Fig. 3B and Additional file 7: Table S5d).

Finally, we rank the potential resistance-virulence risk for all recovered MAGs (Additional file 2: Table S2a). Fig. 3A shows a graph where the nodes are sized according to the risk criteria. For the risk criteria, we highlight the results found for the *Firmicutes* Phylum, where statistically significant

differences between Omnivores vs. Vegetarians and Vegans and between Ketogenic vs Vegetarians and Vegans, with an increased rank observed for the Vegetarians and Vegans MAGs were found. However, there was no difference between Omnivores and Ketogenic, nor between Vegans and Vegetarians (Additional file 7: Table S5e and Additional file 8: Fig. S3b-c). Finally, gSpreadComp compiled all potential plasmid-mediated HGT for the target gene (ARGs, in this use case) and the VFs at a defined taxonomical level (Additional file 9: Tables S6a for ARGs HGT events and Additional file 9: Table S6b for VFs HGT events). We removed the libraries that recovered less than 12 MAGs before the HGT analysis to reduce comparison bias due to limited MAG reconstruction. After filtering, all diets had an average of 26 MAGs per sample. However, Vegans and Vegetarians had 12 ARG plasmid-mediated HGT per sample, while Omnivores had 3.88 and Ketogenic 1,84 (Additional file 9: Table S6c). We observed a significant increase in the ARGs and VFs involved in potential plasmid-mediated HGT in the Vegans and Vegetarians compared to Ancient, Omnivore, and Ketogenic. Following, we performed pairwise Bonferroni statistical comparisons related to the HGT events between the diets (Additional file 9: Table S6c-e and Additional file 8: Fig. S3b-e). All pairwise comparisons against Vegans or Vegetarians were significant (adjusted p-value < 0.05), but there was no significant difference among any other comparison, nor between Vegans and Vegetarians. Similarly, Vegans and Vegetarians had significantly more VFs plasmid-mediated HGT events per sample (Additional file 9: Table S6d-e). Additionally, gSpreadComp allowed for the calculation of the pairwise comparisons related to the occurrence of HGT events per defined taxonomical level (Family) per diet (Additional file 9: Table S6f-h). We identified HGT events of VFs, and a significant difference was observed for the cases in Table 2. In the HGT events of ARGs, a significant difference was only accessed for *Ruminococcaceae* in Omnivore and Vegans and *Lachnospiraceae* in Vegetarians and Ketogenic.

Discussion

The gSpreadComp. gSpreadComp was designed for two main goals: (i) to facilitate comparative genomics and (ii) to integrate high throughput sequencing information into microbiome relative

resistance-virulence risk ranking, with a focus on the potential presence of antimicrobial resistance genes and virulence factors.

At its core, gSpreadComp integrates genome annotation, gene prevalence normalization, and sequence comparison into a streamlined approach, thereby reducing the complexities often associated with disparate tools. Furthermore, the tool introduced a systematic methodology to quantify gene spread, a crucial aspect in understanding gene dispersion populations.

Second, gSpreadComp effectively utilizes whole-genome sequencing (WGS) data by providing a standardized method to rank potential microbial communities of concern using metagenomic samples. By highlighting hotspots of resistance and virulence factors, it narrows the focus for subsequent hypothesis testing through laboratory-based assessments. While not performing risk assessments directly, gSpreadComp may guide more targeted and efficient laboratory studies, ultimately improving resource allocation and preventive measures. Finally, tracking plasmid-mediated HGT can contribute insights into antimicrobial resistance, or any target gene, transfer routes that remain largely uncharted. gSpreadComp also contributes to identifying key disseminating taxa and potential propagation pathways. Such knowledge is vital for developing strategies to combat the rise of antimicrobial-resistant pathogens and constructing more comprehensive microbial risk assessment models [40].

Naturally, while gSpreadComp's main strengths lie in its downstream analysis and unified workflow, it has limitations and biases that should be considered when interpreting results. These may stem from genome recovery techniques, reference databases, or machine learning algorithms used in the tool. As with any bioinformatic approach, we recommend a critical usage.

Critical Usage and Key Considerations

While not a standalone risk assessment tool, gSpreadComp provides a framework for comparing the relative rank associated with resistance and virulence genes across microbial populations. When used with established microbial risk assessment guidelines, gSpreadComp can enhance the depth and precision of risk-rank evaluations. By integrating genomic data analysis with traditional risk assessment

approaches, researchers may gain more comprehensive insights into potential microbial hazards, thereby supporting more informed decision-making in public health, environmental management, and food production contexts [1].

In particular, notice the distinction between relative resistance-virulence risk ranking, which gSpreadComp provides, and risk assessment. While our tool offers insights into the comparative potential resistance-virulence risks within microbial populations based on their genomic profiles, it does not account for all factors considered in a full risk assessment, such as exposure routes, dose-response relationships, and specific environmental conditions [3]. Users should view gSpreadComp's output as a starting point for prioritizing further investigation.

When considering ARG annotation using machine learning algorithms, one must know that ARG prediction accuracy varies per gene and class based on the representation and degree of similarity to known resistance genes in the training databases. For sequences with high identity scores (>50%) to the training data, both alignment-based methods, such as BLAST, and classification-based approaches, such as DeepARG or ARG-SHINE perform well, with around 95% of accuracy [36]. However, classification models tend to perform better for sequences with low identity scores. For instance, sequences conferring resistance to bacitracin, beta-lactams, and MLS are more represented in the databases and more accurately predicted by DeepARG than resistances such as triclosan or quinolone. The more drastic improvement of classification-based methods is in reducing false negative rates, while keeping overall high precision. For long ARG-like sequences, DeepARG-LS achieved 0.97 ± 0.03 precision and 0.99 ± 0.01 recall for bacitracin, beta-lactamase, chloramphenicol, and aminoglycoside, while the best hit approach achieved perfect precision, but 0.48 ± 0.2 recall [32]. This significant difference in recall is particularly crucial when annotating MAGs, which are often fragmented. Importantly, the presence of an ARG does not necessarily equate to phenotypic resistance but also depends on gene expression and host factors and potential bias in the resistance genotype–phenotype concordance on less characterized taxa [41].

Generally, using machine learning-based methods for the classification of biological sequences, while promising, have challenges and limitations. Classifying plasmids can be particularly challenging since they usually exhibit high genetic diversity [38] and shared sequence segments between plasmids and chromosomes. Tools like Plasflow and PlasClass provide a promising alternative for detecting more diverged plasmids via learning patterns beyond sequence similarity but tend to have decreased precision. On the other hand, hybrid methods, like PLASMe, tend to be computationally more costly. Consequently, users should be aware of these methodological differences when interpreting results and consider the strengths and limitations of each approach in the context of their specific research questions. For gSpreadComp, as an auxiliary tool for hypothesis generation, we decided to initially deploy it with the machine learning-based method Plasflow for its comparative results with PlasClass, but with slightly higher recall [35]. However, as the plasmid detection tools rapidly evolve, we expect to update the gSpreadComp plasmid detection module in the future.

Similarly, machine learning-based methods have been used for VF annotation [42–44]. However, to the best of our knowledge, less work has been done on the reliability of those tools when applied to MAGs, specifically when looking for individual VF. Therefore, for VF annotation, we implemented a best-hit-based method in gSpreadComp, potentially increasing the number of false negatives for the sake of precision.

Use Case: gSpreadComp in the human gut microbiome of subjects with different diets. Previous studies have suggested potential links between diet and antibiotic resistance patterns, with some focusing on meat consumption [45–47]. Simultaneously, growing evidence shows that uncooked produce could contribute to higher HGT events and potential antibiotic resistance spread [48–51]. While these findings provide interesting hypotheses, our use of gSpreadComp aims to demonstrate a streamlined approach for analyzing resistance gene spread across diverse groups and draw attention to potential resistance-virulence transmissibility hotspots rather than to draw definitive conclusions about diet-resistance relationships.

Antimicrobial resistance spread

We identified multidrug and glycopeptide resistance genes as ubiquitous in fecal samples from subjects of every diet, including Ancient. Glycopeptide antibiotics have been mainly used to treat multidrug-resistant Gram-positive infections, and increased resistance occurrence has already become a cause of concern [52]. Specifically, its overuse in the livestock industry has already been pointed out almost 20 years ago [53]. Glycopeptide resistance genes were, however, also found in permafrost from > 10,000 years ago [54]. In addition, an extensive metagenomic study of soil, ocean, and animal sources found that glycopeptide resistance-related genes were prevalent in all samples, accounting for 17% of global resistant sequences, second only to multidrug resistance efflux pumps [55].

When analyzing resistance with at least a 0.05 increase in the spread in one particular diet, we observed a specific increase in bacitracin resistance for Vegans (0.7 – widespread), followed by Omnivore (0.64 – widespread), and then the subjects from the other three diets (0.55 on average). Interestingly, bacitracin is not typically used orally but instead applied topically in ointments [56]. In addition, bacitracin has been extensively used as an animal feed additive [57]. Although still under the "low" widespread category previously established, tetracycline resistance genes were more disseminated in Omnivores, 0.51, while subjects preferring the other diets had a similar spread of 0.40, considered "common." Tetracycline is typically used for therapeutic purposes but is reportedly frequently added to livestock feed at doses below therapeutic levels, and it has been used as a growth enhancer for swine, poultry, and aquaculture mainly in the last century [58].

When we grouped the subjects with diets exposed to animal meat (Ketogenic and Omnivore) against the non-exposed (Vegans and Vegetarians), we saw an increase in spread for the MLS, aminoglycoside, and mupirocin resistance. It is relevant to notice that MLS was considered ubiquitous-widespread and aminoglycoside widespread-common in all diets. MLS has been used in cattle and pig husbandry in Europe [59]. Similarly, a 2023 study has explored aminoglycoside detection in several animal muscles, tissues, honey, milk, and other food sources. They were able to detect the antibiotic in 17% of the samples. Most of these samples were retrieved from cattle and swine [60]. The mupirocin

resistance was less spread than the others mentioned. We considered mupirocin in the sparse-common range for all diets.

In our investigation of ARG classes, we observed an elevated spread of diaminopyrimidines that exhibited a more pronounced distribution among vegetarians and vegans, closely followed by omnivores and a lower spread in the ketogenic diet group. A recent study found ubiquitously accumulating diaminopyrimidines, fluoroquinolones, and sulfonamides in rice farms [61]. The study found a higher accumulation of fluoroquinolones and sulfonamide. Consistent with our results, the ancient subjects exhibited the highest prevalence of sulfonamide, 0.37, followed by Vegans, 0.31, and Vegetarians, 0.24.

It is worth noticing that although there are specific differences in resistance spread, all modern diets showed a similar overall spread distribution. On the other hand, by calculating the average ARG class spread in the modern diets, we saw a systematic increase in spread in the modern samples compared to the Ancient diet (10-20% increase). These findings exemplify gSpreadComp's capacity to quantify and compare ARG spread across diverse samples. However, it's crucial to emphasize that these observations showcase the tool's capabilities rather than draw definitive conclusions about diet-resistance relationships. The patterns identified by gSpreadComp can serve as starting points for more comprehensive studies, incorporating additional data sources and experimental validation to fully understand the complex interplay between diet and antimicrobial resistance.

Virulence factor and resistance-virulence risk ranking

Our results revealed a nuanced relationship between diet, the distribution of VFs, and the calculated resistance-virulence potential risk in the human gut microbiome. The average number of unique VFs was statistically similar among the diets. However, *Bacteroidota* associated with subjects from the Ketogenic diet had a statistically higher number of unique VFs than subjects with other diets. Moreover, bacteria with high virulence potential consistently exhibited the highest number of unique antibiotic resistances, irrespective of the subject's diet. Although alarming, this might be expected, as pathogenic bacteria should constantly be exposed to selective pressure.

In ranking relative resistance-virulence potential risk in our dataset, the tool consistently ranked higher risk to known potential pathogenic species. Interestingly, the subtle effects of diet on risk are evidenced in the *Firmicutes* Phylum. A risk difference emerged between Omnivores and Vegetarians/Vegans, and similarly between those on the Ketogenic diet and Vegetarians/Vegans. However, no significant risk disparity was observed when comparing meat-consuming and non-meat diets. These observations demonstrate gSpreadComp's ability to detect nuanced patterns that could inform more targeted investigations.

Finally, our data indicated that Vegans and Vegetarians have significantly more ARGs and VFs involved in potential plasmid-mediated HGT than Ancient, Omnivore, and Ketogenic groups. Specifically, a higher HGT potential was observed for the *Ruminococcaceae* and *Lachnospiraceae* families. These findings echo some of the discoveries of Reid et al. [49], which highlighted the predilection of produce from supermarkets to harbor *E. coli* strains endowed with virulence plasmid carriage, thereby providing a potential conduit for HGT. Raid et al. also discussed the possibility of producing drug-resistant *E. coli* from animal manure fertilizers, contaminated irrigation water, and wildlife. Specifically, they characterized resistant *E. coli* from supermarket-bought, ready-to-eat cilantro, arugula, and mixed salad from two German cities [49]. Another study underscored produce as a reservoir of transferable antibiotic resistance genes, further elucidating the plausible link between plant-based diets and amplified incidences of ARG in plasmid-mediated HGT owing to higher exposure to the transferable resistome inherent in produce [48]. Blau et al. found an impressive diversity of self-transmissible multiple resistance plasmids in bacteria associated with produce that is consumed raw. Finally, Blau et al. discussed the possibility of multiple resistance plasmids being exogenously captured by *E. coli* and transferred to gut bacteria, thus spreading resistance [48].

Although, to the best of our knowledge, no direct study comparing the abundance of plasmids in the human gut and soil was made, several studies indicated the potential increase in the abundance of plasmids in soil environments [62, 63]. Therefore, we hypothesize that gut microbiomes from plant-

based diets have a higher chance of participating in plasmid-mediated HGT and indicate that targeted research should be performed to confirm or deny this hypothesis.

Tools Comparison. In comparative genomics, gSpreadComp gives a step forward as a tool that integrates genome annotation, gene spread calculation, virulence factor identification, plasmid-mediated HGT detection, and antimicrobial resistance-virulence risk ranking. While previously mentioned existing tools have limitations, such as applicability to single taxa or reliance on reference genomes, gSpreadComp offers a comprehensive approach to applying comparative genomics to the entire microbiome. To our knowledge, PathoFact [42] and MetaCHIP [25] are the closest counterparts to gSpreadComp; however, they have different focal points. PathoFact focuses on virulence and resistance gene prediction, while MetaCHIP can detect HGT events directly in a microbiome community in a reference-independent way. gSpreadComp bridges, while focusing on these approaches, offering a comprehensive analysis platform for microbial genomic studies.

gSpreadComp and PathoFact both target ARGS, VF, and MGE annotation in microbial genome analysis, sharing similar objectives. Both approaches utilize key tools like PlasFlow for plasmid identification, DeepARG for antimicrobial resistance genes annotation, and the Virulence Factors Database (VFDB) for annotating virulence factors, which yield similar results in these aspects. However, gSpreadComp adds a unique dimension with its resistance-virulence risk ranking using TOPSIS, and gene spread calculation, and detailed downstream analysis. PathoFact, on the other hand, emphasizes precision in virulence and toxin prediction through a blend of HMM profiles and machine-learning approaches.

Against MetaCHIP, gSpreadComp focuses on plasmid-mediated HGT. While MetaCHIP provides robust HGT detection by combining similarity and phylogenetic approaches, gSpreadComp adds value by directly linking these events to sample metadata, which is crucial for comparative genomics and useful for non-specialist users like clinicians. Naturally, the HGT events detected by gSpreadComp should be present in the results from MetaCHIP.

gSpreadComp's streamlined approach makes it a versatile tool that addresses gaps left by existing methodologies. The approach is particularly advantageous for non-bioinformaticians, as it simplifies complex analyses, making the data accessible and actionable for a broader audience. While gSpreadComp offers a comprehensive approach, it is not intended to replace specialized tools. Instead, it aims to complement existing methodologies by providing an integrated approach for microbial genomic analysis. Users should consider their specific research questions and requirements when choosing the most appropriate tool or combination of tools for their studies. The analyses performed using gSpreadComp are not conclusive, but serve to raise testable hypotheses and focus subsequent laboratory experimentation. By identifying potential antimicrobial resistance and virulence factors, along with their likely bacterial hosts, gSpreadComp narrows the search space for targeted experimental validation.

Conclusion

gSpreadComp combines genome annotation, gene prevalence normalization, and target (i.e., diet) analysis into a comprehensive workflow for quantifying gene spread and assessing potential resistance-virulence risk-ranking in microbial communities. The tool's modular design allows for flexibility and future updates. The tool's application to explore dietary impacts on gut microbiome antibiotic resistance demonstrated its ability to identify complex patterns across different dietary groups. Moreover, nuanced evidence suggested that meat and uncooked produce influence resistance-virulence spread, particularly concerning plasmid-mediated HGT, emphasizing the intricate relationship between diet and microbial dynamics in the human gut. However, it is crucial to emphasize that these findings are intended to showcase gSpreadComp's capabilities, rather than draw definitive conclusions about diet-resistance relationships.

The patterns identified by gSpreadComp can serve as valuable starting points for more comprehensive studies, incorporating larger sample sizes or focused experiments, additional data sources, and experimental validation. As with any bioinformatics tool, results should be interpreted cautiously and used to guide hypothesis generation and further investigation. gSpreadComp aims to

complement existing methodologies by providing an integrated platform for microbial genomic analysis, potentially benefiting a wide range of users.

Data and Methods

Implementation

The gSpreadComp. gSpreadComp is designed for UNIX-based systems. The user can refer to the Manual (https://gitfront.io/r/JonasK/VCX9CmPdTvk6/gSpreadComp/) for detailed instructions. Fundamentally, our approach works in six modular steps. (i) Prokaryotic genome taxonomy assignment, (ii) genome quality estimation, (iii) ARGs annotation, (iv) Plasmid and chromosome classification, (v) Virulence Factors annotation, and (vi) downstream analysis, which involves target-based gene spread analysis, plasmid-mediated HGT of the target gene and VF, prokaryotic resistance-virulence risk-ranking and report generation.

Each module can be applied separately. Consequently, as new sequence classification tools surge, gSpreadComp downstream analysis can continue to be used independently. Another advantage of a modular implementation is that the approach can be easily updated. Fig. 1 indicates the gSpreadComp structure. The approach was written in Bash and R (version 4.2.2) [64]. Finally, we use conda [65] (conda 22.11.1) environments to install all necessary software dependencies and third-party software wherever possible. Using conda allows software management with different and potentially conflicting dependencies in the same system.

In step (i), the user can directly assign taxonomy using GTDB-tk [66] and format the result table automatically. In step (ii), gSpreadComp orchestrates CheckM [67] to estimate prokaryotic genome quality and format the resulting files. Following step (iii), the user can automatically annotate ARG and format its resulting files. To minimize the risk of false positive ARG prediction, gSpreadComp uses the DeepARG-LS [32] with the following parameter values: a minimum of 80% prediction probability, an e-value alignment lower than 1e-10, and a percent identity of 35% or higher [33].

In step (iv), plasmids are predicted using PlasFlow with default parameters (i.e., 0.7 probability threshold) [34]. PlasFlow uses only genomic signatures to identify bacterial plasmids using a neural network model with increased performance compared to similar tools [34]. In addition, this tool is also optimized for metagenomic data, the type of data we expect to use mainly with gSpreadComp. Following, in step (v), we use the Victors' Virulence Factors (VF) database (Downloaded in December 2022) [68] and the Virulence Factors Database (Downloaded in December 2022) [69] to annotate VF on provided genomes. We use the protein sequences from both databases from their core dataset associated with experimentally verified virulence factors. We use BLASTX [70] with an e-value of 1e-50 as the cutoff to locate the VFs.

Finally, in step (vi), gSpreadComp starts by optionally filtering out genomes based on the quality (Completeness – 5*Contamination > 50). It can then remove samples based on the total number of genomes per sample (by default, no sample is removed). Next, we calculated the normalized prevalence of the target gene in a defined group ($\boldsymbol{P_{group,\,gene}}$). It considers the presence or absence of the target gene in a genome divided by the total number of genomes in a group, similar to the definition used by Danko et al. [4]. A Bonferroni-adjusted t-test is used pairwise to compare the target gene prevalence across the groups. When the adjusted p-value was less than 0.05, we assigned a significant difference between the groups. The user can refer to the Manual (https://gitfront.io/r/JonasK/VCX9CmPdTvk6/gSpreadComp/) for a detailed description of the intermediate files generated.

$$\boldsymbol{P_{group,\,gene} = \frac{\sum Genome_{group,gene}}{\sum Genome_{group}}}$$

We use the defined weighted average prevalence (WAP) to estimate the gene spread per taxonomical level per target metadata group, as described by Magnúsdóttir et al. [31]. $\boldsymbol{P_i}$ is the gene prevalence per specified taxonomical group, $\boldsymbol{T}$ is the number of unique taxa in the defined taxonomical level.

$$\boldsymbol{WAP} = \sum_{i=1}^{T} \frac{P_i \times \sum Genome_i}{T}$$

Finally, gSpreadComp extracts what we defined as "Resistance-virulence Risk Factors" for each genome. Those are the genetic potential related to the target gene – represented by the number of unique target genes – the virulence potential – represented by the number of unique VFs – the potential of transmitting the target gene – represented by the number of unique target genes located in plasmids – the potential of transmitting virulence potential – represented by the number of unique VFs located in plasmids. We use the taxonomical distances to the species in the NCBI pathogens database [27] to define the reference potential pathogens. Finally, we use the Technique for Order Preference by Similarity to Ideal Solution (TOPSIS) [28] to rank the resistance-virulence risk from the genomes. Essentially, we extract from each genome ($\boldsymbol{g_i}$) its resistance-virulence risk factors ($\boldsymbol{f_j}$), $\boldsymbol{g_i} = \{\boldsymbol{f_{i,1}}, \boldsymbol{f_{i,2}}, \ldots, \boldsymbol{f_{i,n}}\}$, with n resistance-virulence risk factors.

Following this, we normalized the resistance-virulence risk factors using:

$$\boldsymbol{f_{ij}} = \frac{\boldsymbol{f_{ij}}}{\sqrt{\sum_{i=1}^{m} \boldsymbol{f_{ij}^2}}}$$

Where $\boldsymbol{f_{ij}}$ is the value of the $\boldsymbol{j^{th}}$ risk factor for the $\boldsymbol{i^{th}}$ genome, an $\boldsymbol{m}$ is the total number of genomes. Then, we computed the weighted normalized decision matrix. The defined weights, $\boldsymbol{W} = \{\boldsymbol{w_1}, \boldsymbol{w_2}, \ldots \boldsymbol{w_n}\}$, as the average of the resistance-virulence risk factors extracted from the reference potential pathogens. The weighted normalized decision matrix is represented by

$$\boldsymbol{v_{ij}} = \boldsymbol{w_j} \times \boldsymbol{r_{ij}}$$

We defined the ideal, $\boldsymbol{A^*} = \{\boldsymbol{v_1^*}, \boldsymbol{v_2^*}, \ldots \boldsymbol{v_n^*}\}$, and the negative-ideal, $\boldsymbol{A^-} = \{\boldsymbol{v_1^-}, \boldsymbol{v_2^-}, \ldots \boldsymbol{v_n^-}\}$, solutions as $\boldsymbol{v_j^*} = \max_i(\boldsymbol{v_{ij}})$ and $\boldsymbol{v_j^-} = \min_i(\boldsymbol{v_{ij}})$.

Next, for each genome, we calculate the separation from the ideal solution ($\boldsymbol{S_i^*}$) and from the negative-ideal solution ($\boldsymbol{S_i^-}$) as:

$$S_i^* = \sqrt{\sum_{j=1}^{n} (v_{ij} - v_j^*)^2}$$

$$S_i^- = \sqrt{\sum_{j=1}^{n} (v_{ij} - v_j^-)^2}$$

Finally, the prokaryotic risk (R_i) is the relative closeness to the ideal solution.

$$R_i = \frac{S_i^-}{S_i^* + S_i^-}$$

The genome with the highest R_i value ranks higher in the microbial community resistance-virulence risk scale. We used the TOPSIS implementation in the MCDA R package.

To extract the plasmid-mediated HGT events, we implemented a similar heuristic in gSpreadComp as defined by Smillie et al. [71]. Briefly, one recent HGT event could be identified between two distantly related genomes (from a defined taxonomical level) through the shared region of DNA corresponding to an annotated sequence with 99% or greater similarity.

Lastly, gSpreadComp uses the files, metrics, and figures to generate an HTML report automatically from the rmarkdown [72] package.

Use Case: gSpreadComp in the human gut microbiome of subjects with different diets.

The gSpreadComp approach requires genomes or MAGs in fasta format, the genomes metadata table, including the identification of its source sample and the target feature to be compared, a genome taxonomic assignment table, a genome quality assignment table, and a target gene annotation table.

Metagenome data selection. Initially, we selected metagenomic samples from the human gut of subjects over 18 years old containing information about the host diet using the HumanMetagenomeDB (HMgDB) [73]. We selected only WGS libraries available in the Sequence Read Archive (SRA) (https://www.ncbi.nlm.nih.gov/sra/). After filtering, we remained with metagenomic samples from the

following BioProjects: PRJNA340216, PRJNA397112, PRJNA324129, and PRJNA529487. Afterward, we examined the sample's metadata information on the original studies and assigned the libraries in "Omnivore", "Vegetarian", "Vegan", and "Ketogenic" diet types according to the original studies' definitions. Additionally, we included metagenomic libraries from the AncientMetagenomeDir v20.12 [74]. From the libraries provided on the ancientmetagenome-hostassociated file, we selected those with the following parameters: "sample_host" equal to "Homo sapiens", "community_type" equal to "gut", and "archive" equal to "ENA" or "SRA". We assigned libraries that originated from the AncientMetagenomeDir as "Ancient". The complete table of libraries and accompanying metadata used is in Additional File 1: Table S1. Finally, we downloaded the library reads from the SRA using the SRAtoolkit version 2.10.9 (https://github.com/ncbi/sra-tools).

Data preparation. The Metagenome-assembled genomes (MAGs) were recovered using the Multi-Domain Genome Recovery tool (MuDoGeR) [75]. The raw reads were quality-controlled using metaWrap [76] with default parameters. The reads trimming was performed using TrimGalore [77] with the default settings. After, BMTagger [78] was used with the human build 38 patch release 13 (GRCh38.p13 - https://www.ncbi.nlm.nih.gov/data-hub/genome/GCF_000001405.39/) to remove potential host genomes using default parameters. Following, reads were assembled using metaSpades [79] from within the MuDoGeR approach. Once assembled, the sequence contigs were binned using Metabat2 [80], Maxbin2 [81], and CONCOCT [82]. Then, the recovered bins were refined and dereplicated using MuDoGeR. The bins were quality-checked using CheckM [67], taxonomically assigned using GTDB-tk [66], and assembly statistics calculated with BBTools [83]. Finally, the bins were filtered for MAGs based on the following criteria: at least 50% completeness, less than 10% contamination based on CheckM results, and a quality score higher or equal to 50, where quality score = completeness-5*contamination" [84]. High-quality MAGs were defined as completeness > 90% and contamination < 5. Medium-quality MAGs were defined as completeness >=50 and contamination < 10%. Following, we used the ARG annotation workflow from gSpreadComp to annotate ARGs in each MAG. This means we used DeepARG-LS with a minimum of 80% prediction probability, an e-

value alignment lower than 1e-10, and a percent identity of 35% or higher to minimize the risk of false positives. Next, we used the gSpreadComp methods described in 2.1 to classify plasmid sequences and annotate and format VFs. We removed samples with less than six genome representatives to calculate the gene prevalence per sample. Finally, we integrated the recovered MAGs and the following tables into the gSpreadComp approach: formatted taxonomic assessment, the prokaryotic quality estimation, the ARGs annotation, the plasmid identification, the VFs annotation, and the library metadata. In addition, we also used the gSpreadComp approach to estimate the spread of the ARGs antibiotic resistance group, e.g., bacitracin and glycopeptide, hereafter referred to as ARGs classes.

Availability and requirements

Project name: gSpreadComp

Project home page: https://gitfront.io/r/JonasK/VCX9CmPdTvk6/gSpreadComp/

Operating system(s): Linux.

Programming language: C, Shell, R, Python

Other requirements: Bash, Conda, Mamba, and other packages automatically installed with gSpreadComp

License: GNU GPL v3.0

Data Availability

Metagenome-assembled genomes (MAGs), plasmid, chromosomes identified sequences, antimicrobial resistance genes (ARGs) alignments and database sequences, and virulence factor (VF) annotation and reference database sequences generated and used in this study can be downloaded at https://www.ufz.de/record/dmp/archive/14212 (DOI: 10.48758/ufz.14212). All MAGs are publicly available on the NIH under the BioProject PRJNA1032156.

Additional Files

Additional File 1: 01_Kasmanas_gSpread_AddFile1_Table_S1.xlsx

Table S1. Metadata table from the selected Whole-genome Sequencing (WGS) samples. Columns are standardized as described by Kasmanas et al. (https://webapp.ufz.de/hmgdb/). Samples collected from

the AncientMetagenomeDir had the host_diet assigned as "Ancient". The "sample" column is equivalent to the SRA project_id.

Additional File 2: 02_Kasmanas_gSpread_AddFile2_Table_S2.xlsx

Table S2a. Summary information retrieved from the recovered metagenome-assembled genomes (MAGs). Completeness, Contamination, and Strain.heterogeneity are assigned with CheckM through MuDoGeR (https://github.com/mdsufz/MuDoGeR). Quality and quality.score are determined as described in Methods. The Target column refers to the source patient's diet. The taxonomical information was assigned with GTDBtk through MuDoGeR. Pathogen potential is determined based on the taxonomical distance to reference potential pathogens from the NCBI pathogen database. The risk_criteria ranks the relative resistance-virulence risk calculated as described in Methods. The columns named "unique_*" are defined as "Resistance-virulence Risk Factors" and are used to rank the relative resistance-virulence risk. The Factors are systematically named as follows: "unique_", virulence factors (vf), or target gene (ARGs in our use case), "_in_", sequence type location (i.e., chromosome, plasmids, or unclassified). The last 19 columns are assembly statistics extracted using BBTools (https://sourceforge.net/projects/bbmap/).

Table S2b. Distribution of the number of metagenome-assembled genomes (MAGs) per diet per quality.

Additional File 3: 03_Kasmanas_gSpread_AddFile3_Table_S3.xlsx

Table S3a. DeepARG (https://github.com/gaarangoa/deeparg) antimicrobial resistance gene (ARG) annotation table. gSpreadComp expects to receive a gene annotation csv table in a similar format, indicating the Genome column as "Genome", the target gene column as "Gene_id", and the sequence name from the fasta file where the gene was annotated as "Gene_sequence_location". The probability and identity columns are defined by DeepARG. The "probability" column is the probability that the gene annotation is correct according to their highly accurate ARG predicting model.

Table S3b. Target gene prevalence normalization table per sample (Library). The target gene was the antimicrobial resistance gene (ARG) class (Gene_class) from the DeepARG annotation table. The

present.gene column indicates how many metagenome-assembled genomes (MAGs) in that Library had the specified Gene_class annotated. The Target column indicated the diet from the Library. The t_mags column indicates the total number of MAGs recovered and the gene.genome.prev column indicates the prevalence of the Gene_class

Table S3c. Bonferroni-adjusted t-test pairwise comparison from the antimicrobial resistance genes (ARG) class (Gene_class) prevalence per diet. The y column shows the variable's name used in comparing group1 and group2. The n1 and n2 columns show the number of samples compared. The statistic column is the resulting t-test statistic, and df is the degree of freedom associated with the test. The p is the p-value from the comparison, p.adj is the Bonferroni-adjusted result, and p.adj.signif is an indication of significance ($p < 0.05$).

Additional File 4: 04_Kasmanas_gSpread_AddFile4_Fig_S1.docx

FIG S1. Boxplots from the ARG class prevalence per sample (y-axis) colored by Target Diet. The boxplot title is the ARG class. The statistically significant pairwise comparisons are indicated with the * symbol.

Additional File 5: 05_Kasmanas_gSpread_AddFile5_Table_S4.xlsx

Table S4a. Antimicrobial resistance genes (ARG) class, as assigned by DeepARG (https://github.com/gaarangoa/deeparg), spread at the Phylum level per target diet. The spread was calculated using the weighted average prevalence (WAP).

Table S4b. The antimicrobial resistance genes (ARG) class spread, calculated using weighted average prevalence (WAP) per phyla for the Ancient diet

Table S4c. The antimicrobial resistance genes (ARG) class spread, calculated using weighted average prevalence (WAP) per phyla for the Ketogenic diet

Table S4d. The antimicrobial resistance genes (ARG) class spread, calculated using weighted average prevalence (WAP) per phyla for the Omnivore diet

Table S4e. The antimicrobial resistance genes (ARG) class spread, calculated using weighted average prevalence (WAP) per phyla for the Vegan diet

Table S4f. The antimicrobial resistance genes (ARG) class spread, calculated using weighted average prevalence (WAP) per phyla for the Vegetarian diet

Additional File 6: 06_Kasmanas_gSpread_AddFile6_Fig_S2.docx

FIG S2. Heatmaps containing the spread, calculated as weighted average prevalence (WAP) of the antimicrobial resistance genes (ARG) classes (rows) per phyla (columns) per target diet (title). The number between paratheses after the phyla indicates the number of genomes used for the calculation from that phylum. The number between parentheses from the ARG classes is the average spread for that ARG class.

Additional File 7: 07_Kasmanas_gSpread_AddFile7_Table_S5.xlsx

Table S5a. Virulence Factors (VFs) from the Victors' virulence factors database (https://phidias.us/victors/download.php) (downloaded on December 2022) annotated on the Genomes (Genome column) recovered from the whole-genome sequence (WGS) samples (Library) using BLASTX. Sequence_id indicates the sequence header where the VF (Victor_VF_found) was aligned. Victor_VF_class is the class of the VF given by Victor's database. The values evalue, and bitscore are aligning metrics provided by BLASTX.

Table S5b. The average number of unique Virulence Factors (VFs) per Phylum per Target diet (column avg_unique_VFs). The n column indicates the number of samples used for the calculation, and the column sd_unique_VFs shows the standard deviation from the calculated metrics.

Table S5c. All statistically significant Bonferroni-adjusted t-test pairwise comparisons from the unique number of Virulence Factors (VFs) grouped per Phylum per Target diet. The comparison was made between the diets indicated in group1 and group2. The n1 and n2 columns show the number of samples compared. The p is the p-value from the comparison, p.adj is the Bonferroni-adjusted result, and

p.adj.signif is an indication of significance ($p < 0.05$). The unique number of VFs per Genome can be found in Table S2a.

Table S5d. All statistically significant Bonferroni-adjusted t-test pairwise comparisons from the unique number of antimicrobial resistance genes (ARGs) grouped per pathogenic potential based on the NCBI pathogens database. The comparison was made between the Pathogenic potential indicated in group1 and group2. The n1 and n2 columns show the number of samples compared for group1 and group 2, respectively. The p is the p-value from the comparison, p.adj is the Bonferroni-adjusted result, and p.adj.signif is an indication of significance ($p < 0.05$). Values equal to 0 were extremely close to 0. The unique number of ARGs per Genome can be found in Table S2a.

Table S5e. All statistically significant Bonferroni-adjusted t-test pairwise comparisons from the resistance-virulence risk per Phylum grouped per target diet. The comparison was made between the target diets indicated in Diet 1 and Diet 2. The p.adj is the Bonferroni-adjusted p-value result. The resistance-virulence risk value per Genome can be found in Table S2a.

Additional File 8: 08_Kasmanas_gSpread_AddFile8_Fig_S3.docx

Fig S3a. Boxplots colored by Target diet. The x-axis is grouped by pathogenic potential defined by the taxonomical distance to potential pathogens from the NCBI pathogen database. The y-axis is the number of unique Virulence Factors (VF) per sample.

Fig S3b. Group of boxplots per Phylum that are common to all target diets. The x-axis is grouped and colored by target diet. The y-axis has the calculated resistance-virulence risk metric.

Fig S3c. Density plots of the resistance-virulence risk for each common Phylum colored by target diet. The y-axis indicates the estimated probability density of the respective resistance-virulence risk in the x-axis. Density plots are calculated using the seaborn.kdeplot in Python 3.9.

Fig S3d. Boxplot for the number of antimicrobial resistance genes (ARGs) involved in plasmid-mediated horizontal gene transfer (HGT) events found per sample on the y-axis. The x-axis is grouped and colored by target diet.

Fig S3e. Boxplot for the number of Virulence Factors (VFs) involved in plasmid-mediated horizontal gene transfer (HGT) events found per sample on the y-axis. The x-axis is grouped and colored by target diet.

Additional File 9: 09_Kasmanas_gSpread_AddFile9_Table_S6.xlsx

Table S6a. List of identified antimicrobial resistance genes (ARGs) plasmid-mediated horizontal gene transfer (HGT) events. The library is the sample where the event was found, and Family1 and Family2 are the taxonomical Families involved in the event. The Gene_id column identifies the ARG name involved, and the Target column identifies the target diet from the respective Library.

Table S6b. List of identified virulence factors (VF) plasmid-mediated horizontal gene transfer (HGT) events. The Library is the sample where the event was found, and Family1 and Family2 are the taxonomical Families involved in the event. The Gene_id column identifies the VF name from the Victors database (https://phidias.us/victors/download.php) involved, and the Target column identifies the target diet from the respective Library.

Table S6c. Summary from the horizontal gene transfer (HGT) events per library per target diet after removing the libraries that recovered less than 12 metagenome-assembled genomes.

Table S6d. Bonferroni corrected t-test pairwise comparison between the number of antimicrobial resistance genes (ARGs) horizontal gene transfer (HGT) events grouped by the target diet after removing the libraries that recovered less than 12 metagenome-assembled genomes. Target 1 and Target 2 are the diets compared. T-statistic, P-value, and Adjusted P-value are the statistical test results.

Table S6e. Bonferroni corrected t-test pairwise comparison between the number of virulence factors (VF) horizontal gene transfer (HGT) events grouped by the target diet after removing the libraries that

recovered less than 12 metagenome-assembled genomes. Target 1 and Target 2 are the diets compared. T-statistic, P-value, and Adjusted P-value are the statistical test results.

Table S6f. Summary from the horizontal gene transfer (HGT) events per Family target diet after removing the libraries that recovered less than 12 metagenome-assembled genomes.

Table S6g. Bonferroni corrected t-test pairwise comparison between the number of antimicrobial resistance genes (ARGs) horizontal gene transfer (HGT) events per Family grouped by the target diet after removing the libraries that recovered less than 12 metagenome-assembled genomes. Target 1 and Target 2 are the diets compared for the respective Family. T-statistic, P-value, and Adjusted P-value are the statistical test results. Sample sizes indicate the number of samples used for each Target respectively.

Table S6h. Bonferroni corrected t-test pairwise comparison between the number of virulence factors (VF) horizontal gene transfer (HGT) events per Family grouped by the target diet after removing the libraries that recovered less than 12 metagenome-assembled genomes. Target 1 and Target 2 are the diets compared for the respective Family. T-statistic, P-value, and Adjusted P-value are the statistical test results. Sample sizes indicate the number of samples used for each Target respectively.

Declarations

List of abbreviations

AMR: antimicrobial resistance

ARB: antimicrobial-resistant bacteria

ARGs: antimicrobial resistance genes

HGT: horizontal gene transfer

MAGs: metagenome-assembled genomes

MLS: Macrolides, Lincosamides, Streptogramines

SRA: Sequence Read Archive

TOPSIS: Technique for Order Preference by Similarity to Ideal Solution

VGT: vertical gene transfer

WAP: weighted average prevalence

WGS: whole-genome sequencing

Ethics approval and consent to participate

Not applicable.

Consent for publication

Not applicable.

Competing interests

The authors declare that they have no competing interests.

Funding

JK was supported by the São Paulo Research Foundation (FAPESP; grant 2019/03396-9 and 2022/03534-5). This work was supported by the Helmholtz Young Investigator grant VH-NG-1248 Micro' Big Data', the Deutsche Forschungsgemeinschaft (DFG, German Research Foundation) – project number 460129525, and Canada's International Development Research Centre (IDRC) (Grant No. 109981).

Authors' contributions

JK: investigation, conceptualisation, formal analysis, visualisation, and writing. SM: methodology and critical review. JZ, KS, MS: critical review. PS, AC: supervision and critical review. UR: conceptualisation, supervision, visualisation, writing, and critical review. All authors reviewed and agreed to the content of the manuscript.

Acknowledgements

We thank the de.NBI (German Network for Bioinformatics Infrastructure) and the EVE cluster at the UFZ for their support and computer resources. We would also like to thank Dr. João Saraiva, Martin Bole, and Camila Lima Zanini for their discussions throughout the work development.

References

1. Haas CN, ROSE JB, GERBA CP. Quantitative microbial risk assessment. John Wiley & Sons; 2014.

2. Hamouda MA, Anderson WB, Van Dyke MI, Douglas IP, McFadyen SD, Huck PM. Scenario-based quantitative microbial risk assessment to evaluate the robustness of a drinking water treatment plant. Water Quality Research Journal. 2016;51:81–96.

3. Rantsiou K, Kathariou S, Winkler A, Skandamis P, Saint-Cyr MJ, Rouzeau-Szynalski K, et al. Next generation microbiological risk assessment: opportunities of whole genome sequencing (WGS) for foodborne pathogen surveillance, source tracking and risk assessment. Int J Food Microbiol. 2018;287:3–9.

4. Danko D, Bezdan D, Afshin EE, Ahsanuddin S, Bhattacharya C, Butler DJ, et al. A global metagenomic map of urban microbiomes and antimicrobial resistance. Cell. 2021;184:3376-3393.e17.

5. Lorenzo-Díaz F, Fernández-López C, Lurz R, Bravo A, Espinosa M. Crosstalk between vertical and horizontal gene transfer: plasmid replication control by a conjugative relaxase. Nucleic Acids Res. 2017;45:7774–85.

6. Soucy SM, Huang J, Gogarten JP. Horizontal gene transfer: building the web of life. Nature Reviews Genetics 2015 16:8. 2015;16:472–82.

7. Johnston C, Martin B, Fichant G, Polard P, Claverys JP. Bacterial transformation: distribution, shared mechanisms and divergent control. Nature Reviews Microbiology 2014 12:3. 2014;12:181–96.

8. Bhatt P, Bhandari G, Bhatt K, Maithani D, Mishra S, Gangola S, et al. Plasmid-mediated catabolism for the removal of xenobiotics from the environment. J Hazard Mater. 2021;420:126618.

9. Bottery MJ, Pitchford JW, Friman VP. Ecology and evolution of antimicrobial resistance in bacterial communities. The ISME Journal 2020 15:4. 2020;15:939–48.

10. World Health Organization. Global Antimicrobial Resistance and Use Surveillance System (GLASS) Report 2022. 2022.

11. Huddleston JR. Horizontal gene transfer in the human gastrointestinal tract: Potential spread of antibiotic resistance genes. Infect Drug Resist. 2014;7:167–76.

12. Thanner S, Drissner D, Walsh F. Antimicrobial resistance in agriculture. mBio. 2016;7.

13. Watkins RR, Smith TC, Bonomo RA. On the path to untreatable infections: colistin use in agriculture and the end of 'last resort' antibiotics. http://dx.doi.org/101080/1478721020161216314. 2016;14:785–8.

14. FDA. 2017 Summary Report On Antimicrobials Sold or Distributed for Use in Food-Producing Animals. 2017.

15. Verhaegen M, Bergot T, Liebana E, Stancanelli G, Streissl F, Mingeot-Leclercq MP, et al. On the use of antibiotics to control plant pathogenic bacteria: a genetic and genomic perspective. Front Microbiol. 2023;14:1221478.

16. Brito IL. Examining horizontal gene transfer in microbial communities. Nature Reviews Microbiology 2021 19:7. 2021;19:442–53.

17. Bondarczuk K, Markowicz A, Piotrowska-Seget Z. The urgent need for risk assessment on the antibiotic resistance spread via sewage sludge land application. Environ Int. 2016;87:49–55.

18. Ben Y, Fu C, Hu M, Liu L, Wong MH, Zheng C. Human health risk assessment of antibiotic resistance associated with antibiotic residues in the environment: A review. Environ Res. 2019;169:483–93.

19. Hasan MS, Liu Q, Wang H, Fazekas J, Chen B, Che D. GIST: Genomic island suite of tools for predicting genomic islands in genomic sequences. Bioinformation. 2012;8:203.

20. Langille MGI, Brinkman FSL. IslandViewer: an integrated interface for computational identification and visualization of genomic islands. Bioinformatics. 2009;25:664–5.

21. Podell S, Gaasterland T. DarkHorse: A method for genome-wide prediction of horizontal gene transfer. Genome Biol. 2007;8:1–18.

22. Zhu Q, Kosoy M, Dittmar K. HGTector: An automated method facilitating genome-wide discovery of putative horizontal gene transfers. BMC Genomics. 2014;15:1–18.

23. Bansal MS, Alm EJ, Kellis M. Efficient algorithms for the reconciliation problem with gene duplication, horizontal transfer and loss. Bioinformatics. 2012;28:i283–91.

24. David LA, Alm EJ. Rapid evolutionary innovation during an Archaean genetic expansion. Nature 2010 469:7328. 2010;469:93–6.

25. Song W, Wemheuer B, Zhang S, Steensen K, Thomas T. MetaCHIP: Community-level horizontal gene transfer identification through the combination of best-match and phylogenetic approaches. Microbiome. 2019;7:1–14.

26. Klümper U, Dechesne A, Riber L, Brandt KK, Gülay A, Sørensen SJ, et al. Metal stressors consistently modulate bacterial conjugal plasmid uptake potential in a phylogenetically conserved manner. The ISME Journal 2017 11:1. 2016;11:152–65.

27. Organism Groups - Pathogen Detection - NCBI. https://www.ncbi.nlm.nih.gov/pathogens/organisms/. Accessed 23 Jun 2023.

28. Chakraborty S. TOPSIS and Modified TOPSIS: A comparative analysis. Decision Analytics Journal. 2022;2:100021.

29. Kasmanas JC, Rocha UN da, Toscan R, Sanches DS, Magnusdottir S, Saraiva JP. Simulation of 69 microbial communities indicates sequencing depth and false positives are major drivers of bias in Prokaryotic metagenome-assembled genome recovery. bioRxiv. 2023;:2023.05.02.539054.

30. Meyer F, Fritz A, Deng ZL, Koslicki D, Lesker TR, Gurevich A, et al. Critical Assessment of Metagenome Interpretation: the second round of challenges. Nature Methods 2022 19:4. 2022;19:429–40.

31. Magnúsdóttir S, Saraiva JP, Bartholomäus A, Soheili M, Toscan RB, Zhang J, et al. Metagenome-assembled genomes indicate that antimicrobial resistance genes are highly prevalent among urban bacteria and multidrug and glycopeptide resistances are ubiquitous in most taxa. Front Microbiol. 2023;14:1037845.

32. Arango-Argoty G, Garner E, Pruden A, Heath LS, Vikesland P, Zhang L. DeepARG: A deep learning approach for predicting antibiotic resistance genes from metagenomic data. Microbiome. 2018;6:1–15.

33. Wicaksono WA, Kusstatscher P, Erschen S, Reisenhofer-Graber T, Grube M, Cernava T, et al. Antimicrobial-specific response from resistance gene carriers studied in a natural, highly diverse microbiome. Microbiome. 2021;9:1–14.

34. Krawczyk PS, Lipinski L, Dziembowski A. PlasFlow: predicting plasmid sequences in metagenomic data using genome signatures. Nucleic Acids Res. 2018;46:e35–e35.

35. Pellow D, Mizrahi I, Shamir R. PlasClass improves plasmid sequence classification. PLoS Comput Biol. 2020;16:e1007781.

36. Wang Z, Li S, You R, Zhu S, Zhou XJ, Sun F. ARG-SHINE: improve antibiotic resistance class prediction by integrating sequence homology, functional information and deep convolutional neural network. NAR Genom Bioinform. 2021;3.

37. Alcock BP, Huynh W, Chalil R, Smith KW, Raphenya AR, Wlodarski MA, et al. CARD 2023: expanded curation, support for machine learning, and resistome prediction at the Comprehensive Antibiotic Resistance Database. Nucleic Acids Res. 2023;51:D690.

38. Tang X, Shang J, Ji Y, Sun Y. PLASMe: a tool to identify PLASMid contigs from short-read assemblies using transformer. Nucleic Acids Res. 2023;51:e83–e83.

39. Der Sarkissian C, Velsko IM, Fotakis AK, Vågene ÅJ, Hübner A, Fellows Yates JA. Ancient Metagenomic Studies: Considerations for the Wider Scientific Community. mSystems. 2021;6.

40. Pinilla-Redondo R, Cyriaque V, Jacquiod S, Sørensen SJ, Riber L. Monitoring plasmid-mediated horizontal gene transfer in microbiomes: recent advances and future perspectives. Plasmid. 2018;99:56–67.

41. Nielsen TK, Browne PD, Hansen LH. Antibiotic resistance genes are differentially mobilized according to resistance mechanism. Gigascience. 2022;11:1–17.

42. de Nies L, Lopes S, Busi SB, Galata V, Heintz-Buschart A, Laczny CC, et al. PathoFact: a pipeline for the prediction of virulence factors and antimicrobial resistance genes in metagenomic data. Microbiome. 2021;9:1–14.

43. Xie R, Li J, Wang J, Dai W, Leier A, Marquez-Lago TT, et al. DeepVF: a deep learning-based hybrid framework for identifying virulence factors using the stacking strategy. Brief Bioinform. 2021;22:1–15.

44. Ji B, Pi W, Liu W, Liu Y, Cui Y, Zhang X, et al. HyperVR: a hybrid deep ensemble learning approach for simultaneously predicting virulence factors and antibiotic resistance genes. NAR Genom Bioinform. 2023;5.

45. Van Boeckel TP, Glennon EE, Chen D, Gilbert M, Robinson TP, Grenfell BT, et al. Reducing antimicrobial use in food animals. Science (1979). 2017;357:1350–2.

46. Randad PR, Larsen J, Kaya H, Pisanic N, Ordak C, Price LB, et al. Transmission of Antimicrobial-Resistant Staphylococcus aureus Clonal Complex 9 between Pigs and Humans, United States - Volume 27, Number 3—March 2021 - Emerging Infectious Diseases journal - CDC. Emerg Infect Dis. 2021;27:740–8.

47. Monger XC, Gilbert AA, Saucier L, Vincent AT. Antibiotic Resistance: From Pig to Meat. Antibiotics 2021, Vol 10, Page 1209. 2021;10:1209.

48. Blau K, Bettermann A, Jechalke S, Fornefeld E, Vanrobaeys Y, Stalder T, et al. The Transferable Resistome of Produce. mBio. 2018;9.

49. Reid CJ, Blau K, Jechalke S, Smalla K, Djordjevic SP. Whole Genome Sequencing of Escherichia coli From Store-Bought Produce. Front Microbiol. 2020;10.

50. Njage PMK, Buys EM. Quantitative assessment of human exposure to extended spectrum and AmpC β-lactamases bearing E. coli in lettuce attributable to irrigation water and subsequent horizontal gene transfer. Int J Food Microbiol. 2017;240:141–51.

51. Zhou SYD, Wei MY, Giles M, Neilson R, Zheng F, Zhang Q, et al. Prevalence of Antibiotic Resistome in Ready-to-Eat Salad. Front Public Health. 2020;8:513102.

52. Butler MS, Hansford KA, Blaskovich MAT, Halai R, Cooper MA. Glycopeptide antibiotics: Back to the future. The Journal of Antibiotics 2014 67:9. 2014;67:631–44.

53. Phillips I, Casewell M, Cox T, De Groot B, Friis C, Jones R, et al. Does the use of antibiotics in food animals pose a risk to human health? A critical review of published data. Journal of Antimicrobial Chemotherapy. 2004;53:28–52.

54. Dcosta VM, King CE, Kalan L, Morar M, Sung WWL, Schwarz C, et al. Antibiotic resistance is ancient. Nature 2011 477:7365. 2011;477:457–61.

55. Nesme J, Bastien Cé Cillon S, Delmont TO, Monier J-M, Vogel TM, Simonet P. Report Large-Scale Metagenomic-Based Study of Antibiotic Resistance in the Environment. Current Biology. 2014;24:1096–100.

56. Nguyen R, Khanna NR, Safadi AO, Sun Y. Bacitracin Topical. StatPearls. 2022.

57. Wang Q, Zheng H, Wan X, Huang H, Li J, Nomura CT, et al. Optimization of Inexpensive Agricultural By-Products as Raw Materials for Bacitracin Production in Bacillus licheniformis DW2. Appl Biochem Biotechnol. 2017;183:1146–57.

58. Granados-Chinchilla F, Rodríguez C. Tetracyclines in Food and Feedingstuffs: From Regulation to Analytical Methods, Bacterial Resistance, and Environmental and Health Implications. J Anal Methods Chem. 2017;2017.

59. Pyörälä S, Baptiste KE, Catry B, van Duijkeren E, Greko C, Moreno MA, et al. Macrolides and lincosamides in cattle and pigs: Use and development of antimicrobial resistance. The Veterinary Journal. 2014;200:230–9.

60. Nowacka-Kozak E, Gajda A, Gbylik-Sikorska M. Analysis of Aminoglycoside Antibiotics: A Challenge in Food Control. Molecules. 2023;28:4595.

61. Braun G, Braun M, Kruse J, Amelung W, Renaud FG, Khoi CM, et al. Pesticides and antibiotics in permanent rice, alternating rice-shrimp and permanent shrimp systems of the coastal Mekong Delta, Vietnam. Environ Int. 2019;127:442–51.

62. Shintani M, Nour E, Elsayed T, Blau K, Wall I, Jechalke S, et al. Plant Species-Dependent Increased Abundance and Diversity of IncP-1 Plasmids in the Rhizosphere: New Insights Into Their Role and Ecology. Front Microbiol. 2020;11:590776.

63. Wolters B, Hauschild K, Blau K, Mulder I, Heyde BJ, Sørensen SJ, et al. Biosolids for safe land application: does wastewater treatment plant size matters when considering antibiotics, pollutants,

microbiome, mobile genetic elements and associated resistance genes? Environ Microbiol. 2022;24:1573–89.

64. R Core Team. R: A Language and Environment for Statistical Computing. 2020.

65. Anaconda Software Distribution. Anaconda Documentation. 2020.

66. Chaumeil P-A, Mussig AJ, Hugenholtz P, Parks DH. GTDB-Tk v2: memory friendly classification with the genome taxonomy database. Bioinformatics. 2022;38:5315–6.

67. Parks DH, Imelfort M, Skennerton CT, Hugenholtz P, Tyson GW. CheckM: assessing the quality of microbial genomes recovered from isolates, single cells, and metagenomes. Genome Res. 2015;25:1043–55.

68. Sayers S, Li L, Ong E, Deng S, Fu G, Lin Y, et al. Victors: a web-based knowledge base of virulence factors in human and animal pathogens. Nucleic Acids Res. 2019;47:D693–700.

69. Liu B, Zheng D, Zhou S, Chen L, Yang J. VFDB 2022: a general classification scheme for bacterial virulence factors. Nucleic Acids Res. 2022;50:D912–7.

70. Camacho C, Coulouris G, Avagyan V, Ma N, Papadopoulos J, Bealer K, et al. BLAST+: Architecture and applications. BMC Bioinformatics. 2009;10:1–9.

71. Smillie CS, Smith MB, Friedman J, Cordero OX, David LA, Alm EJ. Ecology drives a global network of gene exchange connecting the human microbiome. Nature 2011 480:7376. 2011;480:241–4.

72. Allaire JJ, Xie Y, Dervieux C, McPherson J, Luraschi J, Ushey K, et al. rmarkdown: Dynamic Documents for R. 2023.

73. Kasmanas JC, Bartholomäus A, Corrêa FB, Tal T, Jehmlich N, Herberth G, et al. HumanMetagenomeDB: a public repository of curated and standardized metadata for human metagenomes. Nucleic Acids Res. 2021;49:D743–50.

74. Fellows Yates JA, Andrades Valtueña A, Vågene ÅJ, Cribdon B, Velsko IM, Borry M, et al. Community-curated and standardised metadata of published ancient metagenomic samples with AncientMetagenomeDir. Sci Data. 2021;8:1–8.

75. Kasmanas JC, Rocha U, Kallies R, Saraiva JP, Toscan RB, Štefanič P, et al. MuDoGeR: Multi-Domain Genome recovery from metagenomes made easy. Mol Ecol Resour. 2024;24:e13904.

76. Uritskiy G V., Diruggiero J, Taylor J. MetaWRAP - A flexible pipeline for genome-resolved metagenomic data analysis. Microbiome. 2018;6:158.

77. Bolger AM, Lohse M, Usadel B. Trimmomatic: a flexible trimmer for Illumina sequence data. Bioinformatics. 2014;30:2114–20.

78. Rotmistrovsky K, Agarwala R. BMTagger: Best Match Tagger for removing human reads from metagenomics datasets. Unpublished. 2011.

79. Nurk S, Meleshko D, Korobeynikov A, Pevzner PA. MetaSPAdes: A new versatile metagenomic assembler. Genome Res. 2017;27:824–34.

80. Kang DD, Li F, Kirton E, Thomas A, Egan R, An H, et al. MetaBAT 2: An adaptive binning algorithm for robust and efficient genome reconstruction from metagenome assemblies. PeerJ. 2019;2019.

81. Wu Y-W, Simmons BA, Singer SW. MaxBin 2.0: an automated binning algorithm to recover genomes from multiple metagenomic datasets. Bioinformatics. 2015;32:605–7.

82. Alneberg J, Bjarnason BS, De Bruijn I, Schirmer M, Quick J, Ijaz UZ, et al. Binning metagenomic contigs by coverage and composition. Nat Methods. 2014;11:1144–6.

83. BBMap download | SourceForge.net. https://sourceforge.net/projects/bbmap/. Accessed 11 Aug 2023.

84. Parks DH, Rinke C, Chuvochina M, Chaumeil P-AA, Woodcroft BJ, Evans PN, et al. Recovery of nearly 8,000 metagenome-assembled genomes substantially expands the tree of life. Nat Microbiol. 2017;2:1533–42.

FIGURE LEGENDS

FIG 1. gSpreadComp workflow. The minimal input necessary for gSpreadComp is the genome and its associated metadata. gSpreadComp offers the possibility to use the built-in prokaryotic taxonomy assignment using GTDBtk, prokaryotic quality estimation using CheckM, Plasmid identification using PlasFlow, and ARGs annotation using DeepARG. Alternatively, any other tool could be used outside gSpreadComp and later used as input to estimate gene spread, microbial resistance-virulence risk, and gene plasmid-mediated HGT events. The gSpreadComp can use the Victors or the VFDB to annotate virulence potential on target genomes and the NCBI human Pathogens Species database as a reference to estimate potential pathogens.

FIG 2. gSpreadComp estimated target gene spread in given metadata. (A) Boxplot from normalized ARG class prevalence per sample colored by diet. The ARG classes are sorted left to right in ascending order according to average ARG class prevalence. (B) Heatmap colored by WAP, used to estimate the spread at the Phylum level across all analyzed diets. Values from 0 to 0.25 are considered Sparse, 0.25 to 0.5 Common, 0.5 to 0.75 Widespread, and 0.75 to 1 Ubiquitous. (C) Boxplot from normalized Bacitracin Prevalence per Sample colored by diet. A pairwise comparison between the diets was made using Bonferroni-adjusted t-test. Statistically significant comparisons (adjusted p-value < 0.05) are indicated by *. The higher the number of *, the closer to 0 the adjusted p-value.

FIG 3. gSpreadComp estimates the resistance-virulence risk from metagenomic-assembled genomes (MAGs). (A) Network representation from the recovered MAGs (nodes) distributed according to the cooccurrence of Antimicrobial resistance genes (ARGs) for the five different diets. The node size represents the resistance-virulence risk of a MAG. The node color represents the Phyla. As expected, the potential pathogens (identified based on the NCBI Pathogen detection database), marked with a star, systematically have a high risk, but in the Ancient diet. The highest resistance-virulence MAG was found in the Omnivore diet, followed by *Proteobacteria* MAGs from Vegans. Interestingly, the number of ARGs in plasmids is the most significant metric to calculate the risk, followed by VFs in plasmids. This indicates that a higher resistance-virulence risk is associated with the presence of the observed

genes in mobile elements. This may be intuitive, as those MAGs are more likely to participate in plasmid-mediated horizontal transmission and contribute to a resistant microbiome. (B) Boxplot from MAGs grouped by pathogen potential on the X-axis and the number of unique ARGs annotated in the MAG on the Y-axis. A "High" pathogen potential indicates that the MAG is from a Specie present in the NCBI Pathogen Detection Database, and "Medium" and "Low" indicate a MAG from the same Genus and Family, respectively. The boxplot indicates high antimicrobial resistance from High potential pathogens compared with the other MAGs. (C) The density of MAGs from the *Bacteroidota* Phylum, based on the total number of annotated unique VFs. The density plot shows a significant negative skew for the Ketogenic diet, while the Ancient diet has a positive skewness, and the other diets tend to have a normal distribution. This indicates that the Ketogenic diet may potentially increase the resistance-virulence risk from *Bacteroidota*.

TABLE 1: Antimicrobial resistance genes (ARG) class spread summary for the common phyla across the different diets. The values represent ARG classes with a spread difference greater than 0.05 in the respective diet for the respective Phylum compared to other diets. While measures were taken to reduce false positives, some errors may still be present, particularly for ARGs underrepresented in databases (e.g., triclosan). Caution is advised when interpreting results from Ancient samples due to potential DNA degradation and contamination issues. It's important to note that despite the 0.05 difference threshold used here, most ARG classes fell into the same spread category (e.g., sparse, common, widespread, or ubiquitous) across all diets, indicating a general consistency in ARG distribution patterns.

**Diet**	**Phylum**		
	**Bacteroidota**	**Firmicutes**	**Proteobacteria**
Omnivore	MLS[a], beta-lactam, fluoroquinolone, multidrug, mupirocin	MLS, aminoglycoside, mupirocin, tetracycline	diaminopyrimidine
Vegan	aminoglycoside, diaminopyrimidine, phenicol, pleuromutilin	bacitracin, diaminopyrimidine	aminoglycoside, bacitracin, fluoroquinolone, pleuromutilin, tetracycline
Ketogenic	bacitracin, glycopeptide, peptide	-	-
Vegetarian	fosmidomycin, tetracycline	fluoroquinolone	mupirocin, phenicol
Ancient	sulfonamide	phenicol, sulfonamide	MLS, beta-lactam, fosmidomycin, glycopeptide, multidrug, peptide, sulfonamide, triclosan

[a] MLS – Macrolides, Lincosamides, Streptogramines

TABLE 2: Pairwise comparison of the number of plasmid-mediated horizontal gene transfer (HGT) events involving virulence factors (VFs) in which specific bacterial families participated. The comparison is made between samples from individuals following different diets. The columns represent the two diets being compared, the adjusted P-value for statistical significance, and the bacterial family involved.

**Diet 1**	**Diet 2**	**Adjusted P-value**[s]	**Family**
Omnivore	Vegetarian	0,0014	*Lachnospiraceae*
Omnivore	Vegan	0,0030	*Lachnospiraceae*
Omnivore	Vegan	0,0032	*Ruminococcaceae*
Vegetarian	Ketogenic	0,0051	*Lachnospiraceae*
Omnivore	Vegetarian	0,0136	*Oscillospiraceae*
Vegan	Ketogenic	0,0142	*Ruminococcaceae*
Vegetarian	Ketogenic	0,020432088	*Oscillospiraceae*
Vegan	Ketogenic	0,043336037	*Lachnospiraceae*
Omnivore	Vegetarian	0,043935883	*Ruminococcaceae*

[a] Bonferroni adjusted t-test

Manuscript marked

Click here to access/download;Manuscript;00_Kasmanas_gSpread_manusc

Streamlining microbial community analysis for potential resistance, virulence, and plasmid-mediated spread through integrated comparative genomics and relative risk ranking using gSpreadComp

Jonas Coelho Kasmanas[a,b,c], Stefanía Magnúsdóttir[a], Junya Zhang[d], Kornelia Smalla[e], Michael Schloter[f], Peter F. Stadler[c], André Carlos Ponce de Leon Ferreira de Carvalho[b], Ulisses Rocha[a]#

[a] Department of Applied and Environmental Microbiology, Helmholtz Centre for Environmental Research – UFZ, Leipzig, Germany.

[b] Institute of Mathematics and Computer Sciences, University of São Paulo, São Carlos, Brazil.

[c] Department of Computer Science and Interdisciplinary Center of Bioinformatics, University of Leipzig, Leipzig, Germany.

[d] Department of Isotope Biogeochemistry, Helmholtz Centre for Environmental Research – UFZ, Leipzig, Germany.

[e] Julius Kühn-Institut, Federal Research Centre for Cultivated Plants, Institute for Epidemiology and Pathogen Diagnostics, Braunschweig, Germany

[f] Helmholtz Center Munich, National Research Center for Environmental Health, Insitute for Comparative Microbiome Analysis, Neuherberg, Germany

#Address correspondence to Ulisses Rocha, ulisses.rocha@ufz.de

Abstract

Background

Comparative genomics, genetic spread analysis, and context-aware ranking are crucial in understanding microbial dynamics' impact on public health. gSpreadComp streamlines the path from in silico analysis to hypothesis generation. By integrating comparative genomics, genome annotation, normalization, plasmid-mediated gene transfer, and microbial resistance-virulence risk ranking into a unified workflow, gSpreadComp facilitates hypothesis generation from complex microbial datasets.

Findings

The gSpreadComp workflow works through six modular steps: taxonomy assignment, genome quality estimation, antimicrobial resistance (AMR) gene annotation, plasmid/chromosome classification, virulence factor annotation, and downstream analysis. Our workflow calculates gene spread using normalized weighted average prevalence and ranks potential resistance-virulence risk by integrating microbial resistance, virulence, and plasmid transmissibility data, and producing an HTML report. As a use case, we analyzed 3,566 metagenome-assembled genomes recovered from human gut microbiomes across diets. Our findings indicated consistent AMR across diets, with diet-specific resistance patterns, such as increased bacitracin in Vegans and tetracycline in Omnivores. Notably, Ketogenic diets showed a slightly higher resistance-virulence rank, while Vegan and Vegetarian encompassed more plasmid-mediated gene transfer.

Conclusion

The gSpreadComp workflow aims to facilitate hypothesis generation for targeted experimental validations by the identification of concerning resistant hotspots in complex microbial datasets. Our study raises attention to a more thorough study of the critical role of diet in microbial community dynamics and the spread of AMR. This research underscores the importance of integrating genomic data into public health strategies to combat AMR. The gSpreadComp workflow is available at https://gitfront.io/r/JonasK/VCX9CmPdTvk6/gSpreadComp/).

Commented [UNdR1]: Once the manuscript would be accepted, we will move this git front to our team's GitHub page:

https://github.com/mdsufz/

KEYWORDS Risk ranking, Comparative Genomics, Gene Spread, Human Microbiome, Virulence Factors, Horizontal Transmission, Metagenome-assembled Genomes, Antimicrobial Resistance.

Background

The microbial safety of food, water, and environmental matrices has been a critical concern for public health since the 1990s [1]. Different approaches, such as quantitative microbial risk assessment, have provided valuable insights and have been fundamental in evidence-based policy-making in public health. Typically, these approaches involve four steps: hazard identification, exposure assessment, dose-response analysis, and risk characterization [2]. However, traditional microbial safety approaches often focus on individual potential pathogens and may overlook community interactions.

Additionally, the advent of high-throughput sequencing technologies has improved our ability to study microbial communities with increased detail. Advances in sequencing technologies can potentially enhance our understanding of microbial ecology and improve microbial analysis's accuracy, precision, and speed [3]. Concomitantly to the advances in understanding microbial ecology, there is a growing need for community-focused approaches to assess relative impacts across diverse microbial populations. When integrated with exposure and dose-response data, such an approach would equip decision-makers and stakeholders with a more robust risk statement. Specifically, identifying antimicrobial resistance (AMR) spread, virulence factor (VF) spread, and genetic mobility factors are crucial for an enhanced microbial risk characterization [3, 4].

Essentially, genetic information is spread among entities by vertical gene transfer (VGT) and horizontal gene transfer (HGT). While VGT is relevant for preserving and stabilizing genetic material, HGT has a crucial role in the evolutionary and adaptive process [5]. Consequently, HGT allows microbes in microbial communities to perform functional leaps and rapidly adapt to new environments. There are three most recognized mechanisms of HGT in prokaryotes: conjugation, transformation, and transduction. Conjugation requires physical contact between the cells. Transformation is the uptake of exogenous DNA, mostly plasmids, from the environment. Transduction is the delivery of genetic material through viruses and virus-like agents[6]. However, even though transduction and transformation events are effective for gene exchange, plasmid-mediated conjugation is often

recognized as the most impactful HGT mechanism [7]. Plasmids often carry genes that allow potential selective advantages, e.g., AMR or heavy metal resistance, VFs, and degradation of xenobiotics [8, 9].

Specifically, the spread of AMR in clinical and natural environments is recognized as one of the most significant global threats [10, 11]. The misuse of antibiotics in agriculture, the environment, and human medicine creates selective pressure on antimicrobial-resistant bacteria (ARB), which may facilitate the HGT of those resistances. Antibiotics are extensively used for farm animals' and plant production [12, 13]. In 2015, a notable trend emerged in the USA, where 62% of antibiotics initially intended for use in food-producing animals were ultimately utilized in human medicine. Additionally, 70% of medically relevant antibiotics were sold for animal use. [14]. Furthermore, while the use of antibiotics in plant agriculture is generally considered lower than in human and veterinary medicine, recent studies suggest it may be more widespread than previously thought. Streptomycin, oxytetracycline, kasugamycin, oxolinic acid, and gentamicin are commonly used in crop protection, particularly in the American and Asian continents [15].

In addition, HGT events provide rapid adaptation to bacteria strains, including AMR, making the development of novel antimicrobials only a short-term palliative measure [16]. Minimizing problematic HGT and the dissemination of antimicrobial resistance genes (ARGs) is the potential long-term solution to the AMR problem. Inherently, advances in understanding plasmid-mediated HGT dynamics in complex microbiomes are a powerful tool to control horizontal dissemination [17, 18].

Although HGT events, specifically plasmid-mediated transfers, play a significant role in the evolution and adaptation of microbial populations, most of those events remain undetected. Consequently, several bioinformatics tools and algorithms were developed to tackle HGT events. For instance, GIST [19] and IslandViewer [20] use genome sequences' features to assign HGT. DarkHorse [21] and HGTector [22] use the "best matches" approach to identify HGT events based on reference genomes. Other methods, such as Ranger-DTL [23] and AnGST [24], require the reconciliation of gene trees with the corresponding species trees to make the HGT prediction. Finally, the MetaCHIP [25] tool combines the results of the similarity and phylogenetic approaches.

A significant limitation of most current HGT detection methods is that they are not directly applicable to the entire microbiome but more for single bacteria taxa. In addition, most methods require reference genomes. For instance, the HGTector [22] is restricted to HGT events from a defined distal group to designated self-group members, while DarkHorse [21] requires a reference genome, a bottleneck for uncultured microorganisms. MetaCHIP [25] can be applied at the community level, given a set of recovered genomes. However, MetaCHIP [25] does not directly integrate its results into relevant sample metadata (i.e., biome, clinical data, environmental condition), reducing its usage for comparative genomics. In addition, none of the mentioned tools allows for direct integration of plasmids-mediated transfer of annotated genes to potential pathogenic bacteria by using, e.g., comparative genomics, which creates a significant barrier for non-bioinformaticians, mainly clinicians, to use such data sets. Finally, plasmids have also been reported to be transferred over considerable taxonomic distances, adding complexity for HGT detection tools to identify plasmid-mediated transfer in complex microbial communities [25, 26].

We designed the gSpreadComp workflow to tackle the following bottlenecks: (a) reduce the barrier of comparative genomics by integrating genome annotation, normalization, and sequence comparison into a unified approach; (b) create a systematic approach to quantify gene spread; (c) integrate plasmid-mediated gene transfer annotation to target metadata with the whole microbiome community in a genome-reference independent approach; (d) provide a resistance-virulence risk ranking metric that considers gene spread, prokaryotic resistance potential, and virulence potential in the era of high-throughput microbial community sequencing. Consequently, gSpreadComp is a UNIX-based workflow for genome analysis (Fig. 1) that provides six modules to perform the following tasks: taxonomy assignment, genome quality estimation, ARGs annotation, plasmid/chromosome classification, VFs annotation, and in-depth downstream analysis.

To demonstrate the potential of the gSpreadComp workflow, we analyzed the spread of ARGs in the human gut microbiome from human subjects with different diets. To this end, we gathered publicly available metagenomes from the human gut containing information about the subjects` diet: (i) Ancient,

diet based on the analysis of ancient human fecal remains; (ii) Ketogenic, fecal samples from subjects with high-fat, and protein, low-carbohydrate diet; (iii) Omnivore, fecal samples from subjects with a diverse diet including both plant and animal-derived foods; (iv) Vegan, fecal samples from subjects with a plant-based diet excluding all animal-derived products; (v) Vegetarian, fecal samples from subjects with diet excluding meat but may include other animal-derived products. We then recovered the metagenomes-assembled genomes (MAGs) from those samples and annotated their ARGs and taxonomy. Finally, those MAGs were analyzed using gSpreadComp using the subjects's diet as the target metadata. Notably, the primary objective of this use case is not to draw definitive conclusions about the relationship between diet and antimicrobial resistance or virulence but to exemplify how gSpreadComp can be applied to complex metagenomic datasets.

Our data revealed antimicrobial resistance, particularly to multidrug and glycopeptide classes, to be widespread across all diets, with specific resistances like bacitracin being more prevalent in Vegans. Additionally, while all diets exhibited similar overall resistance spread, nuances like increased tetracycline resistance in Omnivores were observed. The study also highlighted a complex relationship between diet and VFs, with specific diets showing heightened resistance-virulence risks, like Ketogenic. Finally, Vegans and Vegetarians were associated with a higher potential to participate in plasmid-mediated HGT events, underscoring the significant role of diet in shaping microbial communities and antimicrobial resistance patterns. While further laboratory validation is required, gSpreadComp accelerates the identification of potential targets, streamlining the path from in silico analysis to hypothesis validation through experimental verification.

Findings

The gSpreadComp workflow. The gSpreadComp workflow is a UNIX-based integrated set of tools for genome analysis (Fig. 1). For such, it provides six modules to perform the following tasks: taxonomy assignment, genome quality estimation, ARGs annotation, plasmid/chromosome classification, VFs annotation, and in-depth downstream analysis. This downstream analysis includes target-based gene spread analysis, plasmid-mediated HGT of target genes and VFs, and a prokaryotic resistance-virulence

risk ranking within the analyzed genomes. It is important to note that gSpreadComp is essentially modular, allowing for the integration of new advances in its component methods and tools as they become available.

The spread of target genes was calculated using the genes' weighted average prevalence (WAP), which estimates the gene spread at different taxonomical levels or target groups (e.g., Omnivores, Vegans, Ketogenic). More details can be found in the methods section. For resistance-virulence risk ranking, we defined the "Resistance-virulence potential Factors" that consider target genes (ARGs, by default), virulence, and their plasmid transmissibility potential. Reference potential pathogens were identified by comparing genomes to the NCBI pathogens database [27]. Following, we used the average of the resistance-virulence factors from the reference potential pathogens, based on the NCBI Pathogens Organism groups, as weights and quantified the resistance-virulence risk using the Technique for Order Preference by Similarity to Ideal Solution (TOPSIS) [28], with the resistance-virulence factors serving as input vectors. After the complete downstream analysis, gSpreadComp produced an HTML report.

The gSpreadComp workflow includes an easy-to-use script that downloads and configures the required databases automatically. Consequently, if the user is interested in ARG spread, the only mandatory inputs for gSpreadComp are the genomes and their target metadata. Suppose the user is interested in a different target gene group. In that case, they should provide the annotation table formatted as described in the gSpreadComp documentation. A database update is scheduled to happen every January and July.

Part of gSpreadComp is a wrapper of several bioinformatic approaches. Its modular nature makes it possible to use the tools independently, allowing the use of the tools' main analysis and the related report without the need to annotate it within the software completely. Additionally, the modular nature of the software facilitates its update and allows the more experienced user to integrate only pieces of gSpreadComp into their pipeline. Consequently, gSpreadComp modularity can give the researcher flexibility in their analysis and facilitate the investigator's software management necessities. The

gSpreadComp workflow was designed to support Linux x64 systems. The complete software installation requires approximately 15 GB. The whole database currently requires around 92 GB.

Critical Usage and Key Considerations

Before presenting the experimental results, it is crucial to address specific methodological considerations and limitations in the methods. The gSpreadComp workflow can be used with both complete genomes and MAGs. In our use case, we applied gSpreadComp to MAGs, which are prone to higher potential bias [29], e.g., MAGs are subject to detection bias, particularly for low-abundance organisms, which may lead to the underrepresentation of certain species and their associated ARGs. Additionally, even high-quality MAGs (completeness > 90% and contamination < 5) may be exposed to contig binning error, causing contamination [30]. Finally, there are sample size effects. To mitigate the impact of sample size, gSpreadComp employs normalization techniques and weighted average prevalence for spread calculations [31]. Nevertheless, users should note that the resulting resistance-virulence risk ranking is relative to the analyzed community and not an absolute measure across environments.

The ARG annotation module provided within gSpreadComp uses a machine learning-based classification tool, named DeepARG [32]. While DeepARG has demonstrated high accuracy in ARG prediction, its performance can vary according to the antibiotic category and its representation in the training database. For long sequences (DeepARG-LS), the tool achieved precision and recall values equal to 0.99 in the prediction of different categories of ARGs. To minimize false positives, we followed benchmarked recommendations, including using a minimum 80% prediction probability, an e-value alignment lower than 1e-10, and a percent identity of 35% or higher [33]. It is important to note that the hyperparameters (e.g., prediction probability, e-value alignment) can be altered by the user. Users should interpret results with these constraints in mind. Similarly, for plasmid detection, we currently use PlasFlow [34]. While effective, Plasflow has limitations in classifying shorter sequences. We increased the classification threshold parameter (0.7 > threshold) in our analysis to improve precision, while maintaining the high sensitivity, or recall, offered by PlasFlow's models [34, 35]. However, it must be

observed that automatically classifying plasmids remains complex, with significant advances currently in development. Those approaches were selected because of their ability to streamline large-scale annotation and detection while having higher recall, which is of particular importance when dealing with MAGs.

The gSpreadComp workflow was designed to be modular and extendable, allowing a more straightforward incorporation of additional features in future versions, as the field is rapidly evolving. For instance, ARG detection tools like ARG-SHINE [36]or CARD-RGI [37] or plasmid classification tools like PlasClass [35] or PLASMe [38] can be used, and their results are integrated into gSpreadComp downstream analysis, provided that the users format their data according to the gSpreadComp documentation. We encourage users to consider the strengths and limitations of each tool when interpreting results and to validate findings through complementary experimental approaches when possible. It is important to note that gSpreadComp's downstream results rely on the tools' annotations, and results for simulated communities would closely follow their benchmarked performance.

Use Case: gSpreadComp in the human gut microbiome of subjects with different diets.

To show the potential of gSpreadComp to generate hypotheses, we analyzed the spread of ARGs and virulence factors in the human gut microbiome from subjects with different diets. It is important to mention that the primary objective of this use case is not to draw definitive conclusions about the relationship between diet and antimicrobial resistance or virulence, but to illustrate how gSpreadComp can be applied to complex metagenomic datasets to generate insights that could inform more comprehensive risk assessments.

We recovered MAGs of 17 Ketogenic, 10 Vegans, 40 Vegetarians, and 140 Omnivores subjects from the human gut. In addition, we recovered MAGs from 24 palaeofaeces samples dating from 1300 and 5300 years old (Additional file 1: Table S1). We recovered 3566 MAGs (1806 high and 1760 medium quality) from 231 samples (Additional file 2: Table S2). The taxonomic assignment indicated that the MAGs came from 637 species of 12 Phylum (Additional file 2: Table S2a). According to GTDB-

tk, 594 recovered species were assigned to previously recovered genomes, and 43 species groups found are potentially new.

Our analysis included ancient DNA samples, which present unique challenges. Ancient DNA is typically degraded and fragmented, potentially affecting gene annotation accuracy. Moreover, these samples are highly susceptible to contamination from modern sources and post-mortem microbial colonization. While we have taken steps to address these issues, distinguishing endogenous ancient DNA from contaminants remains challenging. These factors do not invalidate our findings but underscore the need for cautious interpretation, especially when comparing ancient and modern microbiomes [39].

We annotated 356 ARG subtypes distributed in 24 different ARG classes (Additional file 3: Table S3a). In the Ancient samples, we annotated 211 unique ARGs belonging to 22 unique ARG classes. In contrast, Ketogenic had 234 and 18, Omnivores had 320 and 22, Vegans had 238 and 21, and Vegetarians 246 and 20, respectively, in their gut microbiome. We also normalized ARG class prevalence per sample (Additional file 3: Table S3b). We kept only the samples that recovered more than six genomes for further prevalence analysis. Fig. 2A shows the normalized prevalence of the ARG classes per sample for all eating habits. In addition, we performed pairwise ARG class prevalence comparisons for all diets (Additional file 3: Table S3c and Additional file 4: Fig. S1). The bacitracin resistance boxplot comparisons can be found in Fig. 2C.

Further, we estimated the ARG class spread at the Phylum level in gut samples of subjects across the different diets (Additional file 5: Table S4a). We defined the following ranges to describe the distribution of ARG classes: Sparse (0 – 0.25), Common (0.25 – 0.5), Widespread (0.5 – 0.75), and Ubiquitous (0.75 – 1). A heatmap with the distribution at Phylum level value per ARG class for all diets can be found in Fig. 2B. Multidrug and glycopeptide resistance were ubiquitous in all subjects, irrespective of the diet. For further analysis, we excluded ARG classes exhibiting a distribution of less than 0.1 across all dietary patterns. The results revealed that among the diets, Omnivores exhibited the highest spread in seven ARG classes: multidrug, MLS (Macrolides, Lincosamides, Streptogramins),

phenicol, aminoglycoside, tetracycline, and mupirocin. In contrast, Vegans demonstrated the highest spread in four ARG classes: glycopeptide, bacitracin, diaminopyrimidine, and fluoroquinolone. For the remaining dietary patterns, the Ketogenic diet had the highest spread in two ARG classes (pleuromutilin and beta-lactam), the Vegetarian diet in two (peptide and fosmidomycin), and the Ancient subjects in one (sulfonamide). However, considering only the ARG classes with at least a 5% difference between all other diets, bacitracin is more spread in Vegans, tetracycline in Omnivore, and sulfonamide in Ancient. When we compared Ketogenic and Omnivore (Meat eaters) against Vegans and Vegetarians (Not meat eaters) according to the mean spread value, we observed that meat eaters had a higher spread for MLS, aminoglycoside, and mupirocin, and non-meat eaters for diaminopyrimidine.

Finally, gSpreadComp also allowed us to individually compare the spread of ARGs among Phylum (Additional file 5: Tables S4b-f and Additional file 6: Fig S2). The results are summarised in Table 1. The subsequent results that gSpreadComp provided were the annotation of VF. (Additional file 7: Table S5a). The average number of unique VFs annotated per diet were: 479.75 ± 116.41 for Ancient, 444.56 ± 88.03 for Ketogenic, 444.54 ± 106.24 for Omnivore, 475.86 ± 163.95 for Vegan, and 438.13 ± 108.40 for Vegetarian. We also verified the average number of unique VFs per Phylum per diet (Additional file 7: Table S5b). Specifically, *Bacteroidota* related to the Ketogenic diet had statistically more unique VFs than all the other diets (Fig. 3C and Additional file 7: Table S5c). Additionally, gSpreadComp calculated all the statistical significance comparisons associated with the unique number of VFs (Additional file 7: Table S5c). We verified, as expected, that MAGs with high pathogenic potential, irrespective of the diet, have a higher number of unique VFs in the gut samples (Additional file 2: Table S2a and Additional file 8: Fig S3a). More interestingly, we observed that irrespective of the diet, highly virulent bacteria were also the ones with statistically more ARGs in the respective gut samples (Fig. 3B and Additional file 7: Table S5d).

Finally, we rank the potential resistance-virulence risk for all recovered MAGs (Additional file 2: Table S2a). Fig. 3A shows a graph where the nodes are sized according to the risk criteria. For the risk criteria, we highlight the results found for the *Firmicutes* Phylum, where statistically significant

differences between Omnivores vs. Vegetarians and Vegans and between Ketogenic vs Vegetarians and Vegans, with an increased rank observed for the Vegetarians and Vegans MAGs were found. However, there was no difference between Omnivores and Ketogenic, nor between Vegans and Vegetarians (Additional file 7: Table S5e and Additional file 8: Fig. S3b-c). Finally, gSpreadComp compiled all potential plasmid-mediated HGT for the target gene (ARGs, in this use case) and the VFs at a defined taxonomical level (Additional file 9: Tables S6a for ARGs HGT events and Additional file 9: Table S6b for VFs HGT events). We removed the libraries that recovered less than 12 MAGs before the HGT analysis to reduce comparison bias due to limited MAG reconstruction. After filtering, all diets had an average of 26 MAGs per sample. However, Vegans and Vegetarians had 12 ARG plasmid-mediated HGT per sample, while Omnivores had 3.88 and Ketogenic 1,84 (Additional file 9: Table S6c). We observed a significant increase in the ARGs and VFs involved in potential plasmid-mediated HGT in the Vegans and Vegetarians compared to Ancient, Omnivore, and Ketogenic. Following, we performed pairwise Bonferroni statistical comparisons related to the HGT events between the diets (Additional file 9: Table S6c-e and Additional file 8: Fig. S3b-e). All pairwise comparisons against Vegans or Vegetarians were significant (adjusted p-value < 0.05), but there was no significant difference among any other comparison, nor between Vegans and Vegetarians. Similarly, Vegans and Vegetarians had significantly more VFs plasmid-mediated HGT events per sample (Additional file 9: Table S6d-e). Additionally, gSpreadComp allowed for the calculation of the pairwise comparisons related to the occurrence of HGT events per defined taxonomical level (Family) per diet (Additional file 9: Table S6f-h). We identified HGT events of VFs, and a significant difference was observed for the cases in Table 2. In the HGT events of ARGs, a significant difference was only accessed for *Ruminococcaceae* in Omnivore and Vegans and *Lachnospiraceae* in Vegetarians and Ketogenic.

Discussion

The gSpreadComp. gSpreadComp was designed for two main goals: (i) to facilitate comparative genomics and (ii) to integrate high throughput sequencing information into microbiome relative

resistance-virulence risk ranking, with a focus on the potential presence of antimicrobial resistance genes and virulence factors.

At its core, gSpreadComp integrates genome annotation, gene prevalence normalization, and sequence comparison into a streamlined approach, thereby reducing the complexities often associated with disparate tools. Furthermore, the tool introduced a systematic methodology to quantify gene spread, a crucial aspect in understanding gene dispersion populations.

Second, gSpreadComp effectively utilizes whole-genome sequencing (WGS) data by providing a standardized method to rank potential microbial communities of concern using metagenomic samples. By highlighting hotspots of resistance and virulence factors, it narrows the focus for subsequent hypothesis testing through laboratory-based assessments. While not performing risk assessments directly, gSpreadComp may guide more targeted and efficient laboratory studies, ultimately improving resource allocation and preventive measures. Finally, tracking plasmid-mediated HGT can contribute insights into antimicrobial resistance, or any target gene, transfer routes that remain largely uncharted. gSpreadComp also contributes to identifying key disseminating taxa and potential propagation pathways. Such knowledge is vital for developing strategies to combat the rise of antimicrobial-resistant pathogens and constructing more comprehensive microbial risk assessment models [40].

Naturally, while gSpreadComp's main strengths lie in its downstream analysis and unified workflow, it has limitations and biases that should be considered when interpreting results. These may stem from genome recovery techniques, reference databases, or machine learning algorithms used in the tool. As with any bioinformatic approach, we recommend a critical usage.

Critical Usage and Key Considerations

While not a standalone risk assessment tool, gSpreadComp provides a framework for comparing the relative rank associated with resistance and virulence genes across microbial populations. When used with established microbial risk assessment guidelines, gSpreadComp can enhance the depth and precision of risk-rank evaluations. By integrating genomic data analysis with traditional risk assessment

approaches, researchers may gain more comprehensive insights into potential microbial hazards, thereby supporting more informed decision-making in public health, environmental management, and food production contexts [1].

In particular, notice the distinction between relative resistance-virulence risk ranking, which gSpreadComp provides, and risk assessment. While our tool offers insights into the comparative potential resistance-virulence risks within microbial populations based on their genomic profiles, it does not account for all factors considered in a full risk assessment, such as exposure routes, dose-response relationships, and specific environmental conditions [3]. Users should view gSpreadComp's output as a starting point for prioritizing further investigation.

When considering ARG annotation using machine learning algorithms, one must know that ARG prediction accuracy varies per gene and class based on the representation and degree of similarity to known resistance genes in the training databases. For sequences with high identity scores (>50%) to the training data, both alignment-based methods, such as BLAST, and classification-based approaches, such as DeepARG or ARG-SHINE perform well, with around 95% of accuracy [36]. However, classification models tend to perform better for sequences with low identity scores. For instance, sequences conferring resistance to bacitracin, beta-lactams, and MLS are more represented in the databases and more accurately predicted by DeepARG than resistances such as triclosan or quinolone. The more drastic improvement of classification-based methods is in reducing false negative rates, while keeping overall high precision. For long ARG-like sequences, DeepARG-LS achieved 0.97 ± 0.03 precision and 0.99 ± 0.01 recall for bacitracin, beta-lactamase, chloramphenicol, and aminoglycoside, while the best hit approach achieved perfect precision, but 0.48 ± 0.2 recall [32]. This significant difference in recall is particularly crucial when annotating MAGs, which are often fragmented. Importantly, the presence of an ARG does not necessarily equate to phenotypic resistance but also depends on gene expression and host factors and potential bias in the resistance genotype–phenotype concordance on less characterized taxa [41].

Generally, using machine learning-based methods for the classification of biological sequences, while promising, have challenges and limitations. Classifying plasmids can be particularly challenging since they usually exhibit high genetic diversity [38] and shared sequence segments between plasmids and chromosomes. Tools like Plasflow and PlasClass provide a promising alternative for detecting more diverged plasmids via learning patterns beyond sequence similarity but tend to have decreased precision. On the other hand, hybrid methods, like PLASMe, tend to be computationally more costly. Consequently, users should be aware of these methodological differences when interpreting results and consider the strengths and limitations of each approach in the context of their specific research questions. For gSpreadComp, as an auxiliary tool for hypothesis generation, we decided to initially deploy it with the machine learning-based method Plasflow for its comparative results with PlasClass, but with slightly higher recall [35]. However, as the plasmid detection tools rapidly evolve, we expect to update the gSpreadComp plasmid detection module in the future.

Similarly, machine learning-based methods have been used for VF annotation [42–44]. However, to the best of our knowledge, less work has been done on the reliability of those tools when applied to MAGs, specifically when looking for individual VF. Therefore, for VF annotation, we implemented a best-hit-based method in gSpreadComp, potentially increasing the number of false negatives for the sake of precision.

Use Case: gSpreadComp in the human gut microbiome of subjects with different diets. Previous studies have suggested potential links between diet and antibiotic resistance patterns, with some focusing on meat consumption [45–47]. Simultaneously, growing evidence shows that uncooked produce could contribute to higher HGT events and potential antibiotic resistance spread [48–51]. While these findings provide interesting hypotheses, our use of gSpreadComp aims to demonstrate a streamlined approach for analyzing resistance gene spread across diverse groups and draw attention to potential resistance-virulence transmissibility hotspots rather than to draw definitive conclusions about diet-resistance relationships.

Antimicrobial resistance spread

We identified multidrug and glycopeptide resistance genes as ubiquitous in fecal samples from subjects of every diet, including Ancient. Glycopeptide antibiotics have been mainly used to treat multidrug-resistant Gram-positive infections, and increased resistance occurrence has already become a cause of concern [52]. Specifically, its overuse in the livestock industry has already been pointed out almost 20 years ago [53]. Glycopeptide resistance genes were, however, also found in permafrost from > 10,000 years ago [54]. In addition, an extensive metagenomic study of soil, ocean, and animal sources found that glycopeptide resistance-related genes were prevalent in all samples, accounting for 17% of global resistant sequences, second only to multidrug resistance efflux pumps [55].

When analyzing resistance with at least a 0.05 increase in the spread in one particular diet, we observed a specific increase in bacitracin resistance for Vegans (0.7 – widespread), followed by Omnivore (0.64 – widespread), and then the subjects from the other three diets (0.55 on average). Interestingly, bacitracin is not typically used orally but instead applied topically in ointments [56]. In addition, bacitracin has been extensively used as an animal feed additive [57]. Although still under the "low" widespread category previously established, tetracycline resistance genes were more disseminated in Omnivores, 0.51, while subjects preferring the other diets had a similar spread of 0.40, considered "common." Tetracycline is typically used for therapeutic purposes but is reportedly frequently added to livestock feed at doses below therapeutic levels, and it has been used as a growth enhancer for swine, poultry, and aquaculture mainly in the last century [58].

When we grouped the subjects with diets exposed to animal meat (Ketogenic and Omnivore) against the non-exposed (Vegans and Vegetarians), we saw an increase in spread for the MLS, aminoglycoside, and mupirocin resistance. It is relevant to notice that MLS was considered ubiquitous-widespread and aminoglycoside widespread-common in all diets. MLS has been used in cattle and pig husbandry in Europe [59]. Similarly, a 2023 study has explored aminoglycoside detection in several animal muscles, tissues, honey, milk, and other food sources. They were able to detect the antibiotic in 17% of the samples. Most of these samples were retrieved from cattle and swine [60]. The mupirocin

resistance was less spread than the others mentioned. We considered mupirocin in the sparse-common range for all diets.

In our investigation of ARG classes, we observed an elevated spread of diaminopyrimidines that exhibited a more pronounced distribution among vegetarians and vegans, closely followed by omnivores and a lower spread in the ketogenic diet group. A recent study found ubiquitously accumulating diaminopyrimidines, fluoroquinolones, and sulfonamides in rice farms [61]. The study found a higher accumulation of fluoroquinolones and sulfonamide. Consistent with our results, the ancient subjects exhibited the highest prevalence of sulfonamide, 0.37, followed by Vegans, 0.31, and Vegetarians, 0.24.

It is worth noticing that although there are specific differences in resistance spread, all modern diets showed a similar overall spread distribution. On the other hand, by calculating the average ARG class spread in the modern diets, we saw a systematic increase in spread in the modern samples compared to the Ancient diet (10-20% increase). These findings exemplify gSpreadComp's capacity to quantify and compare ARG spread across diverse samples. However, it's crucial to emphasize that these observations showcase the tool's capabilities rather than draw definitive conclusions about diet-resistance relationships. The patterns identified by gSpreadComp can serve as starting points for more comprehensive studies, incorporating additional data sources and experimental validation to fully understand the complex interplay between diet and antimicrobial resistance.

Virulence factor and resistance-virulence risk ranking

Our results revealed a nuanced relationship between diet, the distribution of VFs, and the calculated resistance-virulence potential risk in the human gut microbiome. The average number of unique VFs was statistically similar among the diets. However, *Bacteroidota* associated with subjects from the Ketogenic diet had a statistically higher number of unique VFs than subjects with other diets. Moreover, bacteria with high virulence potential consistently exhibited the highest number of unique antibiotic resistances, irrespective of the subject's diet. Although alarming, this might be expected, as pathogenic bacteria should constantly be exposed to selective pressure.

In ranking relative resistance-virulence potential risk in our dataset, the tool consistently ranked higher risk to known potential pathogenic species. Interestingly, the subtle effects of diet on risk are evidenced in the *Firmicutes* Phylum. A risk difference emerged between Omnivores and Vegetarians/Vegans, and similarly between those on the Ketogenic diet and Vegetarians/Vegans. However, no significant risk disparity was observed when comparing meat-consuming and non-meat diets. These observations demonstrate gSpreadComp's ability to detect nuanced patterns that could inform more targeted investigations.

Finally, our data indicated that Vegans and Vegetarians have significantly more ARGs and VFs involved in potential plasmid-mediated HGT than Ancient, Omnivore, and Ketogenic groups. Specifically, a higher HGT potential was observed for the *Ruminococcaceae* and *Lachnospiraceae* families. These findings echo some of the discoveries of Reid et al. [49], which highlighted the predilection of produce from supermarkets to harbor *E. coli* strains endowed with virulence plasmid carriage, thereby providing a potential conduit for HGT. Raid et al. also discussed the possibility of producing drug-resistant *E. coli* from animal manure fertilizers, contaminated irrigation water, and wildlife. Specifically, they characterized resistant *E. coli* from supermarket-bought, ready-to-eat cilantro, arugula, and mixed salad from two German cities [49]. Another study underscored produce as a reservoir of transferable antibiotic resistance genes, further elucidating the plausible link between plant-based diets and amplified incidences of ARG in plasmid-mediated HGT owing to higher exposure to the transferable resistome inherent in produce [48]. Blau et al. found an impressive diversity of self-transmissible multiple resistance plasmids in bacteria associated with produce that is consumed raw. Finally, Blau et al. discussed the possibility of multiple resistance plasmids being exogenously captured by *E. coli* and transferred to gut bacteria, thus spreading resistance [48].

Although, to the best of our knowledge, no direct study comparing the abundance of plasmids in the human gut and soil was made, several studies indicated the potential increase in the abundance of plasmids in soil environments [62, 63]. Therefore, we hypothesize that gut microbiomes from plant-

based diets have a higher chance of participating in plasmid-mediated HGT and indicate that targeted research should be performed to confirm or deny this hypothesis.

Tools Comparison. In comparative genomics, gSpreadComp gives a step forward as a tool that integrates genome annotation, gene spread calculation, virulence factor identification, plasmid-mediated HGT detection, and antimicrobial resistance-virulence risk ranking. While previously mentioned existing tools have limitations, such as applicability to single taxa or reliance on reference genomes, gSpreadComp offers a comprehensive approach to applying comparative genomics to the entire microbiome. To our knowledge, PathoFact [42] and MetaCHIP [25] are the closest counterparts to gSpreadComp; however, they have different focal points. PathoFact focuses on virulence and resistance gene prediction, while MetaCHIP can detect HGT events directly in a microbiome community in a reference-independent way. gSpreadComp bridges, while focusing on these approaches, offering a comprehensive analysis platform for microbial genomic studies.

gSpreadComp and PathoFact both target ARGS, VF, and MGE annotation in microbial genome analysis, sharing similar objectives. Both approaches utilize key tools like PlasFlow for plasmid identification, DeepARG for antimicrobial resistance genes annotation, and the Virulence Factors Database (VFDB) for annotating virulence factors, which yield similar results in these aspects. However, gSpreadComp adds a unique dimension with its resistance-virulence risk ranking using TOPSIS, and gene spread calculation, and detailed downstream analysis. PathoFact, on the other hand, emphasizes precision in virulence and toxin prediction through a blend of HMM profiles and machine-learning approaches.

Against MetaCHIP, gSpreadComp focuses on plasmid-mediated HGT. While MetaCHIP provides robust HGT detection by combining similarity and phylogenetic approaches, gSpreadComp adds value by directly linking these events to sample metadata, which is crucial for comparative genomics and useful for non-specialist users like clinicians. Naturally, the HGT events detected by gSpreadComp should be present in the results from MetaCHIP.

gSpreadComp's streamlined approach makes it a versatile tool that addresses gaps left by existing methodologies. The approach is particularly advantageous for non-bioinformaticians, as it simplifies complex analyses, making the data accessible and actionable for a broader audience. While gSpreadComp offers a comprehensive approach, it is not intended to replace specialized tools. Instead, it aims to complement existing methodologies by providing an integrated approach for microbial genomic analysis. Users should consider their specific research questions and requirements when choosing the most appropriate tool or combination of tools for their studies. The analyses performed using gSpreadComp are not conclusive, but serve to raise testable hypotheses and focus subsequent laboratory experimentation. By identifying potential antimicrobial resistance and virulence factors, along with their likely bacterial hosts, gSpreadComp narrows the search space for targeted experimental validation.

Conclusion

gSpreadComp combines genome annotation, gene prevalence normalization, and target (i.e., diet) analysis into a comprehensive workflow for quantifying gene spread and assessing potential resistance-virulence risk-ranking in microbial communities. The tool's modular design allows for flexibility and future updates. The tool's application to explore dietary impacts on gut microbiome antibiotic resistance demonstrated its ability to identify complex patterns across different dietary groups. Moreover, nuanced evidence suggested that meat and uncooked produce influence resistance-virulence spread, particularly concerning plasmid-mediated HGT, emphasizing the intricate relationship between diet and microbial dynamics in the human gut. However, it is crucial to emphasize that these findings are intended to showcase gSpreadComp's capabilities, rather than draw definitive conclusions about diet-resistance relationships.

The patterns identified by gSpreadComp can serve as valuable starting points for more comprehensive studies, incorporating larger sample sizes or focused experiments, additional data sources, and experimental validation. As with any bioinformatics tool, results should be interpreted cautiously and used to guide hypothesis generation and further investigation. gSpreadComp aims to

complement existing methodologies by providing an integrated platform for microbial genomic analysis, potentially benefiting a wide range of users.

Data and Methods

Implementation

The gSpreadComp. gSpreadComp is designed for UNIX-based systems. The user can refer to the Manual (https://gitfront.io/r/JonasK/VCX9CmPdTvk6/gSpreadComp/) for detailed instructions. Fundamentally, our approach works in six modular steps. (i) Prokaryotic genome taxonomy assignment, (ii) genome quality estimation, (iii) ARGs annotation, (iv) Plasmid and chromosome classification, (v) Virulence Factors annotation, and (vi) downstream analysis, which involves target-based gene spread analysis, plasmid-mediated HGT of the target gene and VF, prokaryotic resistance-virulence risk-ranking and report generation.

Each module can be applied separately. Consequently, as new sequence classification tools surge, gSpreadComp downstream analysis can continue to be used independently. Another advantage of a modular implementation is that the approach can be easily updated. Fig. 1 indicates the gSpreadComp structure. The approach was written in Bash and R (version 4.2.2) [64]. Finally, we use conda [65] (conda 22.11.1) environments to install all necessary software dependencies and third-party software wherever possible. Using conda allows software management with different and potentially conflicting dependencies in the same system.

In step (i), the user can directly assign taxonomy using GTDB-tk [66] and format the result table automatically. In step (ii), gSpreadComp orchestrates CheckM [67] to estimate prokaryotic genome quality and format the resulting files. Following step (iii), the user can automatically annotate ARG and format its resulting files. To minimize the risk of false positive ARG prediction, gSpreadComp uses the DeepARG-LS [32] with the following parameter values: a minimum of 80% prediction probability, an e-value alignment lower than 1e-10, and a percent identity of 35% or higher [33].

In step (iv), plasmids are predicted using PlasFlow with default parameters (i.e., 0.7 probability threshold) [34]. PlasFlow uses only genomic signatures to identify bacterial plasmids using a neural network model with increased performance compared to similar tools [34]. In addition, this tool is also optimized for metagenomic data, the type of data we expect to use mainly with gSpreadComp. Following, in step (v), we use the Victors' Virulence Factors (VF) database (Downloaded in December 2022) [68] and the Virulence Factors Database (Downloaded in December 2022) [69] to annotate VF on provided genomes. We use the protein sequences from both databases from their core dataset associated with experimentally verified virulence factors. We use BLASTX [70] with an e-value of 1e-50 as the cutoff to locate the VFs.

Finally, in step (vi), gSpreadComp starts by optionally filtering out genomes based on the quality (Completeness – 5*Contamination > 50). It can then remove samples based on the total number of genomes per sample (by default, no sample is removed). Next, we calculated the normalized prevalence of the target gene in a defined group ($\boldsymbol{P_{group,\,gene}}$). It considers the presence or absence of the target gene in a genome divided by the total number of genomes in a group, similar to the definition used by Danko et al. [4]. A Bonferroni-adjusted t-test is used pairwise to compare the target gene prevalence across the groups. When the adjusted p-value was less than 0.05, we assigned a significant difference between the groups. The user can refer to the Manual (https://gitfront.io/r/JonasK/VCX9CmPdTvk6/gSpreadComp/) for a detailed description of the intermediate files generated.

$$\boldsymbol{P_{group,\,gene}} = \frac{\sum \boldsymbol{Genome_{group,gene}}}{\sum \boldsymbol{Genome_{group}}}$$

We use the defined weighted average prevalence (WAP) to estimate the gene spread per taxonomical level per target metadata group, as described by Magnúsdóttir et al. [31]. $\boldsymbol{P_i}$ is the gene prevalence per specified taxonomical group, $\boldsymbol{T}$ is the number of unique taxa in the defined taxonomical level.

$$\boldsymbol{WAP} = \sum_{i=1}^{T} \frac{\boldsymbol{P_i} \times \sum \boldsymbol{Genome_i}}{\boldsymbol{T}}$$

Finally, gSpreadComp extracts what we defined as "Resistance-virulence Risk Factors" for each genome. Those are the genetic potential related to the target gene – represented by the number of unique target genes – the virulence potential – represented by the number of unique VFs – the potential of transmitting the target gene – represented by the number of unique target genes located in plasmids – the potential of transmitting virulence potential – represented by the number of unique VFs located in plasmids. We use the taxonomical distances to the species in the NCBI pathogens database [27] to define the reference potential pathogens. Finally, we use the Technique for Order Preference by Similarity to Ideal Solution (TOPSIS) [28] to rank the resistance-virulence risk from the genomes. Essentially, we extract from each genome ($\boldsymbol{g_i}$) its resistance-virulence risk factors ($\boldsymbol{f_j}$), $\boldsymbol{g_i} = \{\boldsymbol{f}_{i,1}, \boldsymbol{f}_{i,2}, \ldots, \boldsymbol{f}_{i,n}\}$, with n resistance-virulence risk factors.

Following this, we normalized the resistance-virulence risk factors using:

$$\boldsymbol{f_{ij}} = \frac{\boldsymbol{f_{ij}}}{\sqrt{\sum_{i=1}^{m} \boldsymbol{f_{ij}^2}}}$$

Where $\boldsymbol{f_{ij}}$ is the value of the $\boldsymbol{j^{th}}$ risk factor for the $\boldsymbol{i^{th}}$ genome, an $\boldsymbol{m}$ is the total number of genomes. Then, we computed the weighted normalized decision matrix. The defined weights, $\boldsymbol{W} = \{\boldsymbol{w_1}, \boldsymbol{w_2}, \ldots \boldsymbol{w_n}\}$, as the average of the resistance-virulence risk factors extracted from the reference potential pathogens. The weighted normalized decision matrix is represented by

$$\boldsymbol{v_{ij}} = \boldsymbol{w_j} \times \boldsymbol{r_{ij}}$$

We defined the ideal, $\boldsymbol{A}^* = \{\boldsymbol{v_1^*}, \boldsymbol{v_2^*}, \ldots \boldsymbol{v_n^*}\}$, and the negative-ideal, $\boldsymbol{A}^- = \{\boldsymbol{v_1^-}, \boldsymbol{v_2^-}, \ldots \boldsymbol{v_n^-}\}$, solutions as $\boldsymbol{v_j^*} = \max_i(\boldsymbol{v_{ij}})$ and $\boldsymbol{v_j^-} = \min_i(\boldsymbol{v_{ij}})$.

Next, for each genome, we calculate the separation from the ideal solution ($\boldsymbol{S_i^*}$) and from the negative-ideal solution ($\boldsymbol{S_i^-}$) as:

$$\boldsymbol{S_i^*} = \sqrt{\sum_{j=1}^{n} \left(\boldsymbol{v_{ij}} - \boldsymbol{v_j^*}\right)^2}$$

$$\boldsymbol{S_i^-} = \sqrt{\sum_{j=1}^{n} \left(\boldsymbol{v_{ij}} - \boldsymbol{v_j^-}\right)^2}$$

Finally, the prokaryotic risk ($\boldsymbol{R_i}$) is the relative closeness to the ideal solution.

$$\boldsymbol{R_i} = \frac{\boldsymbol{S_i^-}}{\boldsymbol{S_i^*} + \boldsymbol{S_i^-}}$$

The genome with the highest $\boldsymbol{R_i}$ value ranks higher in the microbial community resistance-virulence risk scale. We used the TOPSIS implementation in the MCDA R package.

To extract the plasmid-mediated HGT events, we implemented a similar heuristic in gSpreadComp as defined by Smillie et al. [71]. Briefly, one recent HGT event could be identified between two distantly related genomes (from a defined taxonomical level) through the shared region of DNA corresponding to an annotated sequence with 99% or greater similarity.

Lastly, gSpreadComp uses the files, metrics, and figures to generate an HTML report automatically from the rmarkdown [72] package.

Use Case: gSpreadComp in the human gut microbiome of subjects with different diets.

The gSpreadComp approach requires genomes or MAGs in fasta format, the genomes metadata table, including the identification of its source sample and the target feature to be compared, a genome taxonomic assignment table, a genome quality assignment table, and a target gene annotation table.

Metagenome data selection. Initially, we selected metagenomic samples from the human gut of subjects over 18 years old containing information about the host diet using the HumanMetagenomeDB (HMgDB) [73]. We selected only WGS libraries available in the Sequence Read Archive (SRA) (https://www.ncbi.nlm.nih.gov/sra/). After filtering, we remained with metagenomic samples from the

following BioProjects: PRJNA340216, PRJNA397112, PRJNA324129, and PRJNA529487. Afterward, we examined the sample's metadata information on the original studies and assigned the libraries in "Omnivore", "Vegetarian", "Vegan", and "Ketogenic" diet types according to the original studies' definitions. Additionally, we included metagenomic libraries from the AncientMetagenomeDir v20.12 [74]. From the libraries provided on the ancientmetagenome-hostassociated file, we selected those with the following parameters: "sample_host" equal to "Homo sapiens", "community_type" equal to "gut", and "archive" equal to "ENA" or "SRA". We assigned libraries that originated from the AncientMetagenomeDir as "Ancient". The complete table of libraries and accompanying metadata used is in Additional File 1: Table S1. Finally, we downloaded the library reads from the SRA using the SRAtoolkit version 2.10.9 (https://github.com/ncbi/sra-tools).

Data preparation. The Metagenome-assembled genomes (MAGs) were recovered using the Multi-Domain Genome Recovery tool (MuDoGeR) [75]. The raw reads were quality-controlled using metaWrap [76] with default parameters. The reads trimming was performed using TrimGalore [77] with the default settings. After, BMTagger [78] was used with the human build 38 patch release 13 (GRCh38.p13 - https://www.ncbi.nlm.nih.gov/data-hub/genome/GCF_000001405.39/) to remove potential host genomes using default parameters. Following, reads were assembled using metaSpades [79] from within the MuDoGeR approach. Once assembled, the sequence contigs were binned using Metabat2 [80], Maxbin2 [81], and CONCOCT [82]. Then, the recovered bins were refined and dereplicated using MuDoGeR. The bins were quality-checked using CheckM [67], taxonomically assigned using GTDB-tk [66], and assembly statistics calculated with BBTools [83]. Finally, the bins were filtered for MAGs based on the following criteria: at least 50% completeness, less than 10% contamination based on CheckM results, and a quality score higher or equal to 50, where quality score = completeness-5*contamination" [84]. High-quality MAGs were defined as completeness > 90% and contamination < 5. Medium-quality MAGs were defined as completeness >=50 and contamination < 10%. Following, we used the ARG annotation workflow from gSpreadComp to annotate ARGs in each MAG. This means we used DeepARG-LS with a minimum of 80% prediction probability, an e-

value alignment lower than 1e-10, and a percent identity of 35% or higher to minimize the risk of false positives. Next, we used the gSpreadComp methods described in 2.1 to classify plasmid sequences and annotate and format VFs. We removed samples with less than six genome representatives to calculate the gene prevalence per sample. Finally, we integrated the recovered MAGs and the following tables into the gSpreadComp approach: formatted taxonomic assessment, the prokaryotic quality estimation, the ARGs annotation, the plasmid identification, the VFs annotation, and the library metadata. In addition, we also used the gSpreadComp approach to estimate the spread of the ARGs antibiotic resistance group, e.g., bacitracin and glycopeptide, hereafter referred to as ARGs classes.

Availability and requirements

Project name: gSpreadComp

Project home page: https://gitfront.io/r/JonasK/VCX9CmPdTvk6/gSpreadComp/

Operating system(s): Linux.

Programming language: C, Shell, R, Python

Other requirements: Bash, Conda, Mamba, and other packages automatically installed with gSpreadComp

License: GNU GPL v3.0

Data Availability

Metagenome-assembled genomes (MAGs), plasmid, chromosomes identified sequences, antimicrobial resistance genes (ARGs) alignments and database sequences, and virulence factor (VF) annotation and reference database sequences generated and used in this study can be downloaded at https://www.ufz.de/record/dmp/archive/14212 (DOI: 10.48758/ufz.14212). All MAGs are publicly available on the NIH under the BioProject PRJNA1032156.

Additional Files

Additional File 1: 01_Kasmanas_gSpread_AddFile1_Table_S1.xlsx

Table S1. Metadata table from the selected Whole-genome Sequencing (WGS) samples. Columns are standardized as described by Kasmanas et al. (https://webapp.ufz.de/hmgdb/). Samples collected from

the AncientMetagenomeDir had the host_diet assigned as "Ancient". The "sample" column is equivalent to the SRA project_id.

Additional File 2: 02_Kasmanas_gSpread_AddFile2_Table_S2.xlsx

Table S2a. Summary information retrieved from the recovered metagenome-assembled genomes (MAGs). Completeness, Contamination, and Strain.heterogeneity are assigned with CheckM through MuDoGeR (https://github.com/mdsufz/MuDoGeR). Quality and quality.score are determined as described in Methods. The Target column refers to the source patient's diet. The taxonomical information was assigned with GTDBtk through MuDoGeR. Pathogen potential is determined based on the taxonomical distance to reference potential pathogens from the NCBI pathogen database. The risk_criteria ranks the relative resistance-virulence risk calculated as described in Methods. The columns named "unique_*" are defined as "Resistance-virulence Risk Factors" and are used to rank the relative resistance-virulence risk. The Factors are systematically named as follows: "unique_", virulence factors (vf), or target gene (ARGs in our use case), "_in_", sequence type location (i.e., chromosome, plasmids, or unclassified). The last 19 columns are assembly statistics extracted using BBTools (https://sourceforge.net/projects/bbmap/).

Table S2b. Distribution of the number of metagenome-assembled genomes (MAGs) per diet per quality.

Additional File 3: 03_Kasmanas_gSpread_AddFile3_Table_S3.xlsx

Table S3a. DeepARG (https://github.com/gaarangoa/deeparg) antimicrobial resistance gene (ARG) annotation table. gSpreadComp expects to receive a gene annotation csv table in a similar format, indicating the Genome column as "Genome", the target gene column as "Gene_id", and the sequence name from the fasta file where the gene was annotated as "Gene_sequence_location". The probability and identity columns are defined by DeepARG. The "probability" column is the probability that the gene annotation is correct according to their highly accurate ARG predicting model.

Table S3b. Target gene prevalence normalization table per sample (Library). The target gene was the antimicrobial resistance gene (ARG) class (Gene_class) from the DeepARG annotation table. The

present.gene column indicates how many metagenome-assembled genomes (MAGs) in that Library had the specified Gene_class annotated. The Target column indicated the diet from the Library. The t_mags column indicates the total number of MAGs recovered and the gene.genome.prev column indicates the prevalence of the Gene_class

Table S3c. Bonferroni-adjusted t-test pairwise comparison from the antimicrobial resistance genes (ARG) class (Gene_class) prevalence per diet. The y column shows the variable's name used in comparing group1 and group2. The n1 and n2 columns show the number of samples compared. The statistic column is the resulting t-test statistic, and df is the degree of freedom associated with the test. The p is the p-value from the comparison, p.adj is the Bonferroni-adjusted result, and p.adj.signif is an indication of significance ($p < 0.05$).

Additional File 4: 04_Kasmanas_gSpread_AddFile4_Fig_S1.docx

FIG S1. Boxplots from the ARG class prevalence per sample (y-axis) colored by Target Diet. The boxplot title is the ARG class. The statistically significant pairwise comparisons are indicated with the * symbol.

Additional File 5: 05_Kasmanas_gSpread_AddFile5_Table_S4.xlsx

Table S4a. Antimicrobial resistance genes (ARG) class, as assigned by DeepARG (https://github.com/gaarangoa/deeparg), spread at the Phylum level per target diet. The spread was calculated using the weighted average prevalence (WAP).

Table S4b. The antimicrobial resistance genes (ARG) class spread, calculated using weighted average prevalence (WAP) per phyla for the Ancient diet

Table S4c. The antimicrobial resistance genes (ARG) class spread, calculated using weighted average prevalence (WAP) per phyla for the Ketogenic diet

Table S4d. The antimicrobial resistance genes (ARG) class spread, calculated using weighted average prevalence (WAP) per phyla for the Omnivore diet

Table S4e. The antimicrobial resistance genes (ARG) class spread, calculated using weighted average prevalence (WAP) per phyla for the Vegan diet

Table S4f. The antimicrobial resistance genes (ARG) class spread, calculated using weighted average prevalence (WAP) per phyla for the Vegetarian diet

Additional File 6: 06_Kasmanas_gSpread_AddFile6_Fig_S2.docx

FIG S2. Heatmaps containing the spread, calculated as weighted average prevalence (WAP) of the antimicrobial resistance genes (ARG) classes (rows) per phyla (columns) per target diet (title). The number between paratheses after the phyla indicates the number of genomes used for the calculation from that phylum. The number between parentheses from the ARG classes is the average spread for that ARG class.

Additional File 7: 07_Kasmanas_gSpread_AddFile7_Table_S5.xlsx

Table S5a. Virulence Factors (VFs) from the Victors' virulence factors database (https://phidias.us/victors/download.php) (downloaded on December 2022) annotated on the Genomes (Genome column) recovered from the whole-genome sequence (WGS) samples (Library) using BLASTX. Sequence_id indicates the sequence header where the VF (Victor_VF_found) was aligned. Victor_VF_class is the class of the VF given by Victor's database. The values evalue, and bitscore are aligning metrics provided by BLASTX.

Table S5b. The average number of unique Virulence Factors (VFs) per Phylum per Target diet (column avg_unique_VFs). The n column indicates the number of samples used for the calculation, and the column sd_unique_VFs shows the standard deviation from the calculated metrics.

Table S5c. All statistically significant Bonferroni-adjusted t-test pairwise comparisons from the unique number of Virulence Factors (VFs) grouped per Phylum per Target diet. The comparison was made between the diets indicated in group1 and group2. The n1 and n2 columns show the number of samples compared. The p is the p-value from the comparison, p.adj is the Bonferroni-adjusted result, and

p.adj.signif is an indication of significance ($p < 0.05$). The unique number of VFs per Genome can be found in Table S2a.

Table S5d. All statistically significant Bonferroni-adjusted t-test pairwise comparisons from the unique number of antimicrobial resistance genes (ARGs) grouped per pathogenic potential based on the NCBI pathogens database. The comparison was made between the Pathogenic potential indicated in group1 and group2. The n1 and n2 columns show the number of samples compared for group1 and group 2, respectively. The p is the p-value from the comparison, p.adj is the Bonferroni-adjusted result, and p.adj.signif is an indication of significance ($p < 0.05$). Values equal to 0 were extremely close to 0. The unique number of ARGs per Genome can be found in Table S2a.

Table S5e. All statistically significant Bonferroni-adjusted t-test pairwise comparisons from the resistance-virulence risk per Phylum grouped per target diet. The comparison was made between the target diets indicated in Diet 1 and Diet 2. The p.adj is the Bonferroni-adjusted p-value result. The resistance-virulence risk value per Genome can be found in Table S2a.

Additional File 8: 08_Kasmanas_gSpread_AddFile8_Fig_S3.docx

Fig S3a. Boxplots colored by Target diet. The x-axis is grouped by pathogenic potential defined by the taxonomical distance to potential pathogens from the NCBI pathogen database. The y-axis is the number of unique Virulence Factors (VF) per sample.

Fig S3b. Group of boxplots per Phylum that are common to all target diets. The x-axis is grouped and colored by target diet. The y-axis has the calculated resistance-virulence risk metric.

Fig S3c. Density plots of the resistance-virulence risk for each common Phylum colored by target diet. The y-axis indicates the estimated probability density of the respective resistance-virulence risk in the x-axis. Density plots are calculated using the seaborn.kdeplot in Python 3.9.

Fig S3d. Boxplot for the number of antimicrobial resistance genes (ARGs) involved in plasmid-mediated horizontal gene transfer (HGT) events found per sample on the y-axis. The x-axis is grouped and colored by target diet.

Fig S3e. Boxplot for the number of Virulence Factors (VFs) involved in plasmid-mediated horizontal gene transfer (HGT) events found per sample on the y-axis. The x-axis is grouped and colored by target diet.

Additional File 9: 09_Kasmanas_gSpread_AddFile9_Table_S6.xlsx

Table S6a. List of identified antimicrobial resistance genes (ARGs) plasmid-mediated horizontal gene transfer (HGT) events. The library is the sample where the event was found, and Family1 and Family2 are the taxonomical Families involved in the event. The Gene_id column identifies the ARG name involved, and the Target column identifies the target diet from the respective Library.

Table S6b. List of identified virulence factors (VF) plasmid-mediated horizontal gene transfer (HGT) events. The Library is the sample where the event was found, and Family1 and Family2 are the taxonomical Families involved in the event. The Gene_id column identifies the VF name from the Victors database (https://phidias.us/victors/download.php) involved, and the Target column identifies the target diet from the respective Library.

Table S6c. Summary from the horizontal gene transfer (HGT) events per library per target diet after removing the libraries that recovered less than 12 metagenome-assembled genomes.

Table S6d. Bonferroni corrected t-test pairwise comparison between the number of antimicrobial resistance genes (ARGs) horizontal gene transfer (HGT) events grouped by the target diet after removing the libraries that recovered less than 12 metagenome-assembled genomes. Target 1 and Target 2 are the diets compared. T-statistic, P-value, and Adjusted P-value are the statistical test results.

Table S6e. Bonferroni corrected t-test pairwise comparison between the number of virulence factors (VF) horizontal gene transfer (HGT) events grouped by the target diet after removing the libraries that

recovered less than 12 metagenome-assembled genomes. Target 1 and Target 2 are the diets compared. T-statistic, P-value, and Adjusted P-value are the statistical test results.

Table S6f. Summary from the horizontal gene transfer (HGT) events per Family target diet after removing the libraries that recovered less than 12 metagenome-assembled genomes.

Table S6g. Bonferroni corrected t-test pairwise comparison between the number of antimicrobial resistance genes (ARGs) horizontal gene transfer (HGT) events per Family grouped by the target diet after removing the libraries that recovered less than 12 metagenome-assembled genomes. Target 1 and Target 2 are the diets compared for the respective Family. T-statistic, P-value, and Adjusted P-value are the statistical test results. Sample sizes indicate the number of samples used for each Target respectively.

Table S6h. Bonferroni corrected t-test pairwise comparison between the number of virulence factors (VF) horizontal gene transfer (HGT) events per Family grouped by the target diet after removing the libraries that recovered less than 12 metagenome-assembled genomes. Target 1 and Target 2 are the diets compared for the respective Family. T-statistic, P-value, and Adjusted P-value are the statistical test results. Sample sizes indicate the number of samples used for each Target respectively.

Declarations

List of abbreviations

AMR: antimicrobial resistance

ARB: antimicrobial-resistant bacteria

ARGs: antimicrobial resistance genes

HGT: horizontal gene transfer

MAGs: metagenome-assembled genomes

MLS: Macrolides, Lincosamides, Streptogramines

SRA: Sequence Read Archive

TOPSIS: Technique for Order Preference by Similarity to Ideal Solution

VGT: vertical gene transfer

WAP: weighted average prevalence

WGS: whole-genome sequencing

Ethics approval and consent to participate

Not applicable.

Consent for publication

Not applicable.

Competing interests

The authors declare that they have no competing interests.

Funding

JK was supported by the São Paulo Research Foundation (FAPESP; grant 2019/03396-9 and 2022/03534-5). This work was supported by the Helmholtz Young Investigator grant VH-NG-1248 Micro' Big Data', the Deutsche Forschungsgemeinschaft (DFG, German Research Foundation) – project number 460129525, and Canada's International Development Research Centre (IDRC) (Grant No. 109981).

Authors' contributions

JK: investigation, conceptualisation, formal analysis, visualisation, and writing. SM: methodology and critical review. JZ, KS, MS: critical review. PS, AC: supervision and critical review. UR: conceptualisation, supervision, visualisation, writing, and critical review. All authors reviewed and agreed to the content of the manuscript.

Acknowledgements

We thank the de.NBI (German Network for Bioinformatics Infrastructure) and the EVE cluster at the UFZ for their support and computer resources. We would also like to thank Dr. João Saraiva, Martin Bole, and Camila Lima Zanini for their discussions throughout the work development.

References

1. Haas CN, ROSE JB, GERBA CP. Quantitative microbial risk assessment. John Wiley & Sons; 2014.

2. Hamouda MA, Anderson WB, Van Dyke MI, Douglas IP, McFadyen SD, Huck PM. Scenario-based quantitative microbial risk assessment to evaluate the robustness of a drinking water treatment plant. Water Quality Research Journal. 2016;51:81–96.

3. Rantsiou K, Kathariou S, Winkler A, Skandamis P, Saint-Cyr MJ, Rouzeau-Szynalski K, et al. Next generation microbiological risk assessment: opportunities of whole genome sequencing (WGS) for foodborne pathogen surveillance, source tracking and risk assessment. Int J Food Microbiol. 2018;287:3–9.

4. Danko D, Bezdan D, Afshin EE, Ahsanuddin S, Bhattacharya C, Butler DJ, et al. A global metagenomic map of urban microbiomes and antimicrobial resistance. Cell. 2021;184:3376-3393.e17.

5. Lorenzo-Díaz F, Fernández-López C, Lurz R, Bravo A, Espinosa M. Crosstalk between vertical and horizontal gene transfer: plasmid replication control by a conjugative relaxase. Nucleic Acids Res. 2017;45:7774–85.

6. Soucy SM, Huang J, Gogarten JP. Horizontal gene transfer: building the web of life. Nature Reviews Genetics 2015 16:8. 2015;16:472–82.

7. Johnston C, Martin B, Fichant G, Polard P, Claverys JP. Bacterial transformation: distribution, shared mechanisms and divergent control. Nature Reviews Microbiology 2014 12:3. 2014;12:181–96.

8. Bhatt P, Bhandari G, Bhatt K, Maithani D, Mishra S, Gangola S, et al. Plasmid-mediated catabolism for the removal of xenobiotics from the environment. J Hazard Mater. 2021;420:126618.

9. Bottery MJ, Pitchford JW, Friman VP. Ecology and evolution of antimicrobial resistance in bacterial communities. The ISME Journal 2020 15:4. 2020;15:939–48.

10. World Health Organization. Global Antimicrobial Resistance and Use Surveillance System (GLASS) Report 2022. 2022.

11. Huddleston JR. Horizontal gene transfer in the human gastrointestinal tract: Potential spread of antibiotic resistance genes. Infect Drug Resist. 2014;7:167–76.

12. Thanner S, Drissner D, Walsh F. Antimicrobial resistance in agriculture. mBio. 2016;7.

13. Watkins RR, Smith TC, Bonomo RA. On the path to untreatable infections: colistin use in agriculture and the end of ‘last resort’ antibiotics. http://dx.doi.org/101080/1478721020161216314. 2016;14:785–8.

14. FDA. 2017 Summary Report On Antimicrobials Sold or Distributed for Use in Food-Producing Animals. 2017.

15. Verhaegen M, Bergot T, Liebana E, Stancanelli G, Streissl F, Mingeot-Leclercq MP, et al. On the use of antibiotics to control plant pathogenic bacteria: a genetic and genomic perspective. Front Microbiol. 2023;14:1221478.

16. Brito IL. Examining horizontal gene transfer in microbial communities. Nature Reviews Microbiology 2021 19:7. 2021;19:442–53.

17. Bondarczuk K, Markowicz A, Piotrowska-Seget Z. The urgent need for risk assessment on the antibiotic resistance spread via sewage sludge land application. Environ Int. 2016;87:49–55.

18. Ben Y, Fu C, Hu M, Liu L, Wong MH, Zheng C. Human health risk assessment of antibiotic resistance associated with antibiotic residues in the environment: A review. Environ Res. 2019;169:483–93.

19. Hasan MS, Liu Q, Wang H, Fazekas J, Chen B, Che D. GIST: Genomic island suite of tools for predicting genomic islands in genomic sequences. Bioinformation. 2012;8:203.

20. Langille MGI, Brinkman FSL. IslandViewer: an integrated interface for computational identification and visualization of genomic islands. Bioinformatics. 2009;25:664–5.

21. Podell S, Gaasterland T. DarkHorse: A method for genome-wide prediction of horizontal gene transfer. Genome Biol. 2007;8:1–18.

22. Zhu Q, Kosoy M, Dittmar K. HGTector: An automated method facilitating genome-wide discovery of putative horizontal gene transfers. BMC Genomics. 2014;15:1–18.

23. Bansal MS, Alm EJ, Kellis M. Efficient algorithms for the reconciliation problem with gene duplication, horizontal transfer and loss. Bioinformatics. 2012;28:i283–91.

24. David LA, Alm EJ. Rapid evolutionary innovation during an Archaean genetic expansion. Nature 2010 469:7328. 2010;469:93–6.

25. Song W, Wemheuer B, Zhang S, Steensen K, Thomas T. MetaCHIP: Community-level horizontal gene transfer identification through the combination of best-match and phylogenetic approaches. Microbiome. 2019;7:1–14.

26. Klümper U, Dechesne A, Riber L, Brandt KK, Gülay A, Sørensen SJ, et al. Metal stressors consistently modulate bacterial conjugal plasmid uptake potential in a phylogenetically conserved manner. The ISME Journal 2017 11:1. 2016;11:152–65.

27. Organism Groups - Pathogen Detection - NCBI. https://www.ncbi.nlm.nih.gov/pathogens/organisms/. Accessed 23 Jun 2023.

28. Chakraborty S. TOPSIS and Modified TOPSIS: A comparative analysis. Decision Analytics Journal. 2022;2:100021.

29. Kasmanas JC, Rocha UN da, Toscan R, Sanches DS, Magnusdottir S, Saraiva JP. Simulation of 69 microbial communities indicates sequencing depth and false positives are major drivers of bias in Prokaryotic metagenome-assembled genome recovery. bioRxiv. 2023;:2023.05.02.539054.

30. Meyer F, Fritz A, Deng ZL, Koslicki D, Lesker TR, Gurevich A, et al. Critical Assessment of Metagenome Interpretation: the second round of challenges. Nature Methods 2022 19:4. 2022;19:429–40.

31. Magnúsdóttir S, Saraiva JP, Bartholomäus A, Soheili M, Toscan RB, Zhang J, et al. Metagenome-assembled genomes indicate that antimicrobial resistance genes are highly prevalent among urban bacteria and multidrug and glycopeptide resistances are ubiquitous in most taxa. Front Microbiol. 2023;14:1037845.

32. Arango-Argoty G, Garner E, Pruden A, Heath LS, Vikesland P, Zhang L. DeepARG: A deep learning approach for predicting antibiotic resistance genes from metagenomic data. Microbiome. 2018;6:1–15.

33. Wicaksono WA, Kusstatscher P, Erschen S, Reisenhofer-Graber T, Grube M, Cernava T, et al. Antimicrobial-specific response from resistance gene carriers studied in a natural, highly diverse microbiome. Microbiome. 2021;9:1–14.

34. Krawczyk PS, Lipinski L, Dziembowski A. PlasFlow: predicting plasmid sequences in metagenomic data using genome signatures. Nucleic Acids Res. 2018;46:e35–e35.

35. Pellow D, Mizrahi I, Shamir R. PlasClass improves plasmid sequence classification. PLoS Comput Biol. 2020;16:e1007781.

36. Wang Z, Li S, You R, Zhu S, Zhou XJ, Sun F. ARG-SHINE: improve antibiotic resistance class prediction by integrating sequence homology, functional information and deep convolutional neural network. NAR Genom Bioinform. 2021;3.

37. Alcock BP, Huynh W, Chalil R, Smith KW, Raphenya AR, Wlodarski MA, et al. CARD 2023: expanded curation, support for machine learning, and resistome prediction at the Comprehensive Antibiotic Resistance Database. Nucleic Acids Res. 2023;51:D690.

38. Tang X, Shang J, Ji Y, Sun Y. PLASMe: a tool to identify PLASMid contigs from short-read assemblies using transformer. Nucleic Acids Res. 2023;51:e83–e83.

39. Der Sarkissian C, Velsko IM, Fotakis AK, Vågene ÅJ, Hübner A, Fellows Yates JA. Ancient Metagenomic Studies: Considerations for the Wider Scientific Community. mSystems. 2021;6.

40. Pinilla-Redondo R, Cyriaque V, Jacquiod S, Sørensen SJ, Riber L. Monitoring plasmid-mediated horizontal gene transfer in microbiomes: recent advances and future perspectives. Plasmid. 2018;99:56–67.

41. Nielsen TK, Browne PD, Hansen LH. Antibiotic resistance genes are differentially mobilized according to resistance mechanism. Gigascience. 2022;11:1–17.

42. de Nies L, Lopes S, Busi SB, Galata V, Heintz-Buschart A, Laczny CC, et al. PathoFact: a pipeline for the prediction of virulence factors and antimicrobial resistance genes in metagenomic data. Microbiome. 2021;9:1–14.

43. Xie R, Li J, Wang J, Dai W, Leier A, Marquez-Lago TT, et al. DeepVF: a deep learning-based hybrid framework for identifying virulence factors using the stacking strategy. Brief Bioinform. 2021;22:1–15.

44. Ji B, Pi W, Liu W, Liu Y, Cui Y, Zhang X, et al. HyperVR: a hybrid deep ensemble learning approach for simultaneously predicting virulence factors and antibiotic resistance genes. NAR Genom Bioinform. 2023;5.

45. Van Boeckel TP, Glennon EE, Chen D, Gilbert M, Robinson TP, Grenfell BT, et al. Reducing antimicrobial use in food animals. Science (1979). 2017;357:1350–2.

46. Randad PR, Larsen J, Kaya H, Pisanic N, Ordak C, Price LB, et al. Transmission of Antimicrobial-Resistant Staphylococcus aureus Clonal Complex 9 between Pigs and Humans, United States - Volume 27, Number 3—March 2021 - Emerging Infectious Diseases journal - CDC. Emerg Infect Dis. 2021;27:740–8.

47. Monger XC, Gilbert AA, Saucier L, Vincent AT. Antibiotic Resistance: From Pig to Meat. Antibiotics 2021, Vol 10, Page 1209. 2021;10:1209.

48. Blau K, Bettermann A, Jechalke S, Fornefeld E, Vanrobaeys Y, Stalder T, et al. The Transferable Resistome of Produce. mBio. 2018;9.

49. Reid CJ, Blau K, Jechalke S, Smalla K, Djordjevic SP. Whole Genome Sequencing of Escherichia coli From Store-Bought Produce. Front Microbiol. 2020;10.

50. Njage PMK, Buys EM. Quantitative assessment of human exposure to extended spectrum and AmpC β-lactamases bearing E. coli in lettuce attributable to irrigation water and subsequent horizontal gene transfer. Int J Food Microbiol. 2017;240:141–51.

51. Zhou SYD, Wei MY, Giles M, Neilson R, Zheng F, Zhang Q, et al. Prevalence of Antibiotic Resistome in Ready-to-Eat Salad. Front Public Health. 2020;8:513102.

52. Butler MS, Hansford KA, Blaskovich MAT, Halai R, Cooper MA. Glycopeptide antibiotics: Back to the future. The Journal of Antibiotics 2014 67:9. 2014;67:631–44.

53. Phillips I, Casewell M, Cox T, De Groot B, Friis C, Jones R, et al. Does the use of antibiotics in food animals pose a risk to human health? A critical review of published data. Journal of Antimicrobial Chemotherapy. 2004;53:28–52.

54. Dcosta VM, King CE, Kalan L, Morar M, Sung WWL, Schwarz C, et al. Antibiotic resistance is ancient. Nature 2011 477:7365. 2011;477:457–61.

55. Nesme J, Bastien Cé Cillon S, Delmont TO, Monier J-M, Vogel TM, Simonet P. Report Large-Scale Metagenomic-Based Study of Antibiotic Resistance in the Environment. Current Biology. 2014;24:1096–100.

56. Nguyen R, Khanna NR, Safadi AO, Sun Y. Bacitracin Topical. StatPearls. 2022.

57. Wang Q, Zheng H, Wan X, Huang H, Li J, Nomura CT, et al. Optimization of Inexpensive Agricultural By-Products as Raw Materials for Bacitracin Production in Bacillus licheniformis DW2. Appl Biochem Biotechnol. 2017;183:1146–57.

58. Granados-Chinchilla F, Rodríguez C. Tetracyclines in Food and Feedingstuffs: From Regulation to Analytical Methods, Bacterial Resistance, and Environmental and Health Implications. J Anal Methods Chem. 2017;2017.

59. Pyörälä S, Baptiste KE, Catry B, van Duijkeren E, Greko C, Moreno MA, et al. Macrolides and lincosamides in cattle and pigs: Use and development of antimicrobial resistance. The Veterinary Journal. 2014;200:230–9.

60. Nowacka-Kozak E, Gajda A, Gbylik-Sikorska M. Analysis of Aminoglycoside Antibiotics: A Challenge in Food Control. Molecules. 2023;28:4595.

61. Braun G, Braun M, Kruse J, Amelung W, Renaud FG, Khoi CM, et al. Pesticides and antibiotics in permanent rice, alternating rice-shrimp and permanent shrimp systems of the coastal Mekong Delta, Vietnam. Environ Int. 2019;127:442–51.

62. Shintani M, Nour E, Elsayed T, Blau K, Wall I, Jechalke S, et al. Plant Species-Dependent Increased Abundance and Diversity of IncP-1 Plasmids in the Rhizosphere: New Insights Into Their Role and Ecology. Front Microbiol. 2020;11:590776.

63. Wolters B, Hauschild K, Blau K, Mulder I, Heyde BJ, Sørensen SJ, et al. Biosolids for safe land application: does wastewater treatment plant size matters when considering antibiotics, pollutants,

microbiome, mobile genetic elements and associated resistance genes? Environ Microbiol. 2022;24:1573–89.

64. R Core Team. R: A Language and Environment for Statistical Computing. 2020.

65. Anaconda Software Distribution. Anaconda Documentation. 2020.

66. Chaumeil P-A, Mussig AJ, Hugenholtz P, Parks DH. GTDB-Tk v2: memory friendly classification with the genome taxonomy database. Bioinformatics. 2022;38:5315–6.

67. Parks DH, Imelfort M, Skennerton CT, Hugenholtz P, Tyson GW. CheckM: assessing the quality of microbial genomes recovered from isolates, single cells, and metagenomes. Genome Res. 2015;25:1043–55.

68. Sayers S, Li L, Ong E, Deng S, Fu G, Lin Y, et al. Victors: a web-based knowledge base of virulence factors in human and animal pathogens. Nucleic Acids Res. 2019;47:D693–700.

69. Liu B, Zheng D, Zhou S, Chen L, Yang J. VFDB 2022: a general classification scheme for bacterial virulence factors. Nucleic Acids Res. 2022;50:D912–7.

70. Camacho C, Coulouris G, Avagyan V, Ma N, Papadopoulos J, Bealer K, et al. BLAST+: Architecture and applications. BMC Bioinformatics. 2009;10:1–9.

71. Smillie CS, Smith MB, Friedman J, Cordero OX, David LA, Alm EJ. Ecology drives a global network of gene exchange connecting the human microbiome. Nature 2011 480:7376. 2011;480:241–4.

72. Allaire JJ, Xie Y, Dervieux C, McPherson J, Luraschi J, Ushey K, et al. rmarkdown: Dynamic Documents for R. 2023.

73. Kasmanas JC, Bartholomäus A, Corrêa FB, Tal T, Jehmlich N, Herberth G, et al. HumanMetagenomeDB: a public repository of curated and standardized metadata for human metagenomes. Nucleic Acids Res. 2021;49:D743–50.

74. Fellows Yates JA, Andrades Valtueña A, Vågene ÅJ, Cribdon B, Velsko IM, Borry M, et al. Community-curated and standardised metadata of published ancient metagenomic samples with AncientMetagenomeDir. Sci Data. 2021;8:1–8.

75. Kasmanas JC, Rocha U, Kallies R, Saraiva JP, Toscan RB, Štefanič P, et al. MuDoGeR: Multi-Domain Genome recovery from metagenomes made easy. Mol Ecol Resour. 2024;24:e13904.

76. Uritskiy G V., Diruggiero J, Taylor J. MetaWRAP - A flexible pipeline for genome-resolved metagenomic data analysis. Microbiome. 2018;6:158.

77. Bolger AM, Lohse M, Usadel B. Trimmomatic: a flexible trimmer for Illumina sequence data. Bioinformatics. 2014;30:2114–20.

78. Rotmistrovsky K, Agarwala R. BMTagger: Best Match Tagger for removing human reads from metagenomics datasets. Unpublished. 2011.

79. Nurk S, Meleshko D, Korobeynikov A, Pevzner PA. MetaSPAdes: A new versatile metagenomic assembler. Genome Res. 2017;27:824–34.

80. Kang DD, Li F, Kirton E, Thomas A, Egan R, An H, et al. MetaBAT 2: An adaptive binning algorithm for robust and efficient genome reconstruction from metagenome assemblies. PeerJ. 2019;2019.

81. Wu Y-W, Simmons BA, Singer SW. MaxBin 2.0: an automated binning algorithm to recover genomes from multiple metagenomic datasets. Bioinformatics. 2015;32:605–7.

82. Alneberg J, Bjarnason BS, De Bruijn I, Schirmer M, Quick J, Ijaz UZ, et al. Binning metagenomic contigs by coverage and composition. Nat Methods. 2014;11:1144–6.

83. BBMap download | SourceForge.net. https://sourceforge.net/projects/bbmap/. Accessed 11 Aug 2023.

84. Parks DH, Rinke C, Chuvochina M, Chaumeil P-AA, Woodcroft BJ, Evans PN, et al. Recovery of nearly 8,000 metagenome-assembled genomes substantially expands the tree of life. Nat Microbiol. 2017;2:1533–42.

FIGURE LEGENDS

FIG 1. gSpreadComp workflow. The minimal input necessary for gSpreadComp is the genome and its associated metadata. gSpreadComp offers the possibility to use the built-in prokaryotic taxonomy assignment using GTDBtk, prokaryotic quality estimation using CheckM, Plasmid identification using PlasFlow, and ARGs annotation using DeepARG. Alternatively, any other tool could be used outside gSpreadComp and later used as input to estimate gene spread, microbial resistance-virulence risk, and gene plasmid-mediated HGT events. The gSpreadComp can use the Victors or the VFDB to annotate virulence potential on target genomes and the NCBI human Pathogens Species database as a reference to estimate potential pathogens.

FIG 2. gSpreadComp estimated target gene spread in given metadata. (A) Boxplot from normalized ARG class prevalence per sample colored by diet. The ARG classes are sorted left to right in ascending order according to average ARG class prevalence. (B) Heatmap colored by WAP, used to estimate the spread at the Phylum level across all analyzed diets. Values from 0 to 0.25 are considered Sparse, 0.25 to 0.5 Common, 0.5 to 0.75 Widespread, and 0.75 to 1 Ubiquitous. (C) Boxplot from normalized Bacitracin Prevalence per Sample colored by diet. A pairwise comparison between the diets was made using Bonferroni-adjusted t-test. Statistically significant comparisons (adjusted p-value < 0.05) are indicated by *. The higher the number of *, the closer to 0 the adjusted p-value.

FIG 3. gSpreadComp estimates the resistance-virulence risk from metagenomic-assembled genomes (MAGs). (A) Network representation from the recovered MAGs (nodes) distributed according to the cooccurrence of Antimicrobial resistance genes (ARGs) for the five different diets. The node size represents the resistance-virulence risk of a MAG. The node color represents the Phyla. As expected, the potential pathogens (identified based on the NCBI Pathogen detection database), marked with a star, systematically have a high risk, but in the Ancient diet. The highest resistance-virulence MAG was found in the Omnivore diet, followed by *Proteobacteria* MAGs from Vegans. Interestingly, the number of ARGs in plasmids is the most significant metric to calculate the risk, followed by VFs in plasmids. This indicates that a higher resistance-virulence risk is associated with the presence of the observed

genes in mobile elements. This may be intuitive, as those MAGs are more likely to participate in plasmid-mediated horizontal transmission and contribute to a resistant microbiome. (B) Boxplot from MAGs grouped by pathogen potential on the X-axis and the number of unique ARGs annotated in the MAG on the Y-axis. A "High" pathogen potential indicates that the MAG is from a Specie present in the NCBI Pathogen Detection Database, and "Medium" and "Low" indicate a MAG from the same Genus and Family, respectively. The boxplot indicates high antimicrobial resistance from High potential pathogens compared with the other MAGs. (C) The density of MAGs from the *Bacteroidota* Phylum, based on the total number of annotated unique VFs. The density plot shows a significant negative skew for the Ketogenic diet, while the Ancient diet has a positive skewness, and the other diets tend to have a normal distribution. This indicates that the Ketogenic diet may potentially increase the resistance-virulence risk from *Bacteroidota*.

TABLE 1: Antimicrobial resistance genes (ARG) class spread summary for the common phyla across the different diets. The values represent ARG classes with a spread difference greater than 0.05 in the respective diet for the respective Phylum compared to other diets. While measures were taken to reduce false positives, some errors may still be present, particularly for ARGs underrepresented in databases (e.g., triclosan). Caution is advised when interpreting results from Ancient samples due to potential DNA degradation and contamination issues. It's important to note that despite the 0.05 difference threshold used here, most ARG classes fell into the same spread category (e.g., sparse, common, widespread, or ubiquitous) across all diets, indicating a general consistency in ARG distribution patterns.

**Diet**	**Phylum**		
	**Bacteroidota**	**Firmicutes**	**Proteobacteria**
Omnivore	MLS[a], beta-lactam, fluoroquinolone, multidrug, mupirocin	MLS, aminoglycoside, mupirocin, tetracycline	diaminopyrimidine
Vegan	aminoglycoside, diaminopyrimidine, phenicol, pleuromutilin	bacitracin, diaminopyrimidine	aminoglycoside, bacitracin, fluoroquinolone, pleuromutilin, tetracycline
Ketogenic	bacitracin, glycopeptide, peptide	-	-
Vegetarian	fosmidomycin, tetracycline	fluoroquinolone	mupirocin, phenicol
Ancient	sulfonamide	phenicol, sulfonamide	MLS, beta-lactam, fosmidomycin, glycopeptide, multidrug, peptide, sulfonamide, triclosan

[a] MLS – Macrolides, Lincosamides, Streptogramines

TABLE 2: Pairwise comparison of the number of plasmid-mediated horizontal gene transfer (HGT) events involving virulence factors (VFs) in which specific bacterial families participated. The comparison is made between samples from individuals following different diets. The columns represent the two diets being compared, the adjusted P-value for statistical significance, and the bacterial family involved.

**Diet 1**	**Diet 2**	**Adjusted P-value**[s]	**Family**
Omnivore	Vegetarian	0,0014	*Lachnospiraceae*
Omnivore	Vegan	0,0030	*Lachnospiraceae*
Omnivore	Vegan	0,0032	*Ruminococcaceae*
Vegetarian	Ketogenic	0,0051	*Lachnospiraceae*
Omnivore	Vegetarian	0,0136	*Oscillospiraceae*
Vegan	Ketogenic	0,0142	*Ruminococcaceae*
Vegetarian	Ketogenic	0,020432088	*Oscillospiraceae*
Vegan	Ketogenic	0,043336037	*Lachnospiraceae*
Omnivore	Vegetarian	0,043935883	*Ruminococcaceae*

[a] Bonferroni adjusted t-test

Figure 1

Click here to access/download;Figure;01_Kasmanas_gSpread_Fig_1.png

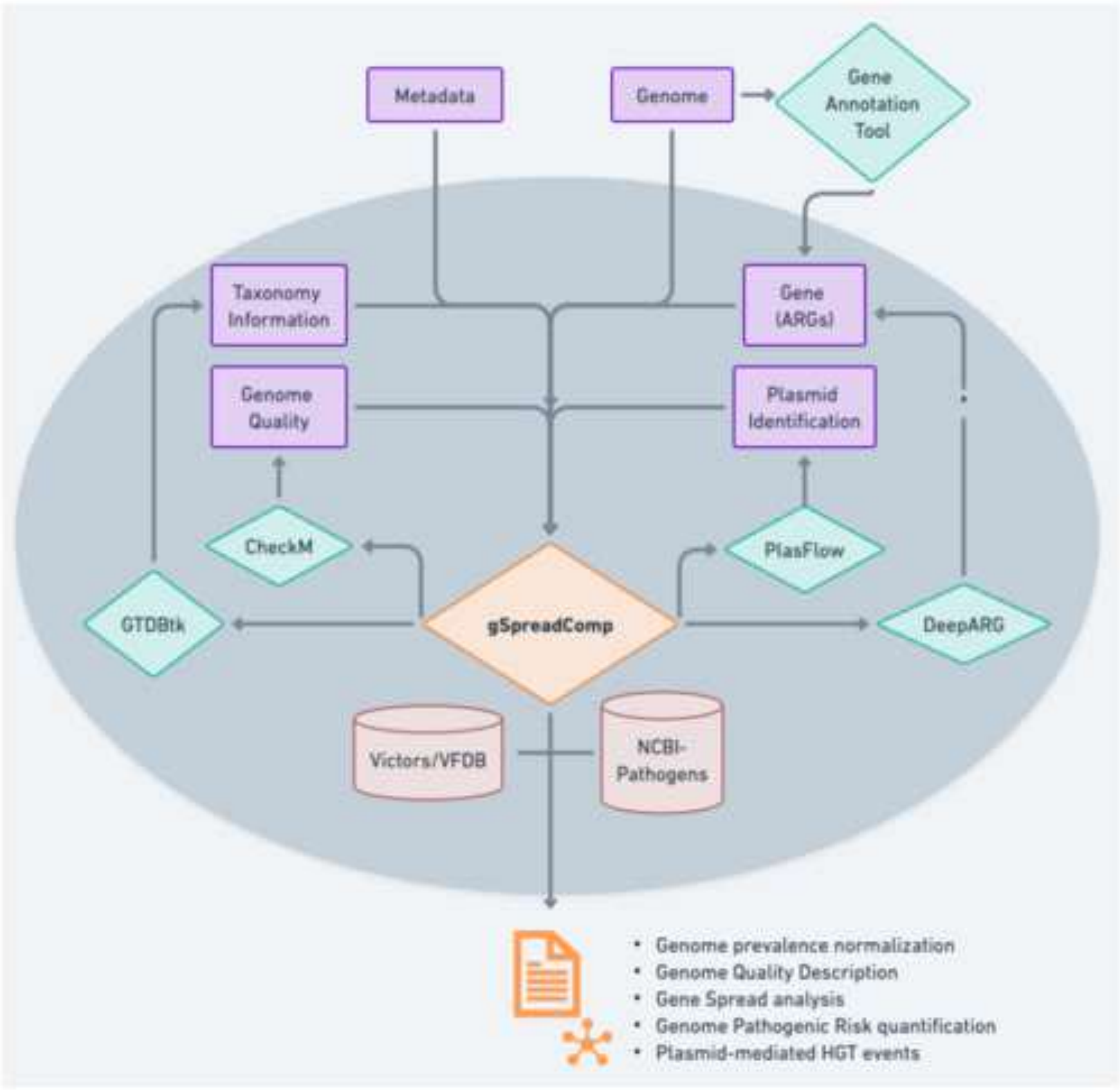

Figure 2

Click here to access/download;Figure;02_Kasmanas_gSpread_Fig_2.png

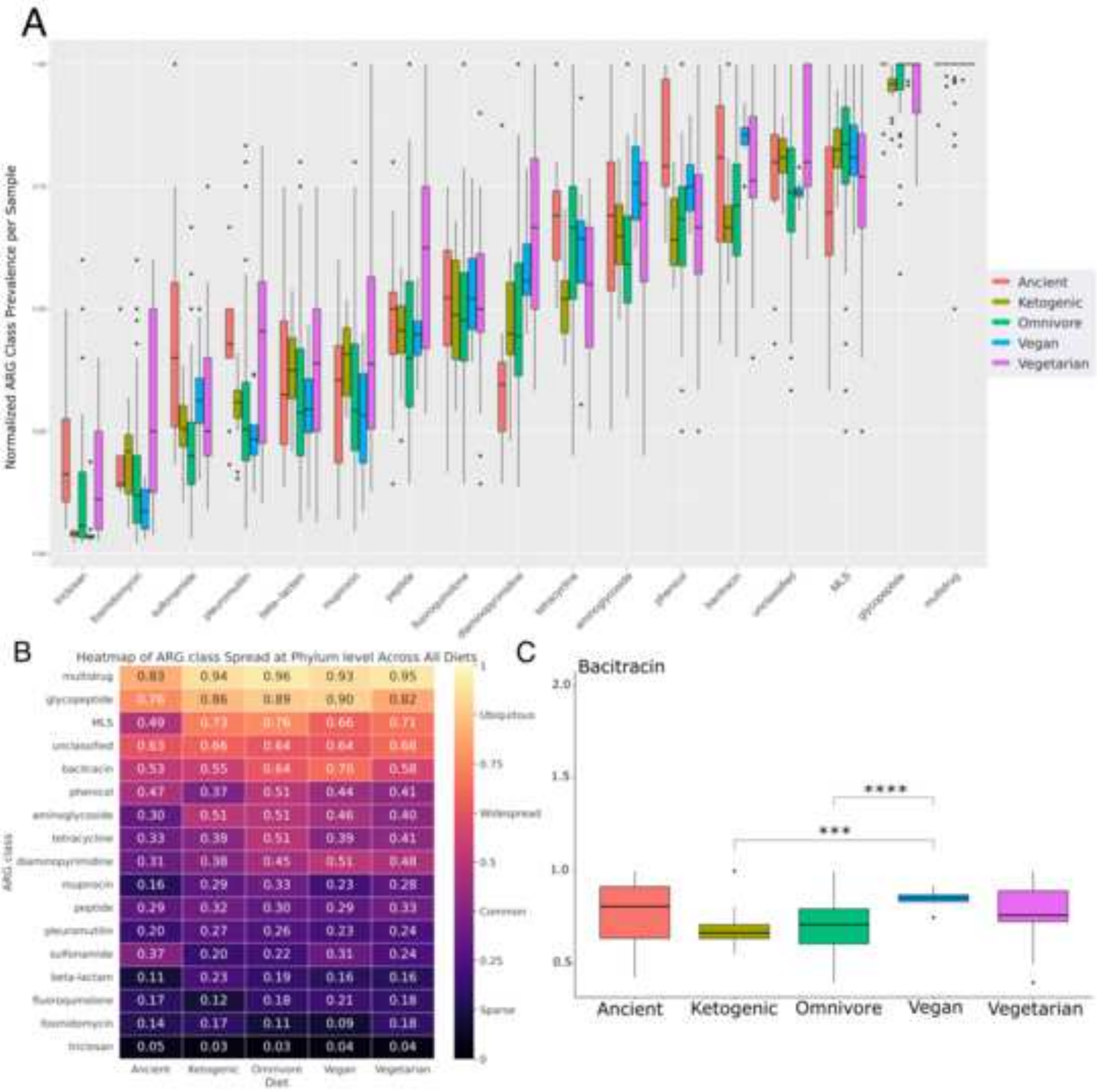

Figure 3

Click here to access/download;Figure;03_Kasmanas_gSpread_Fig_3.png

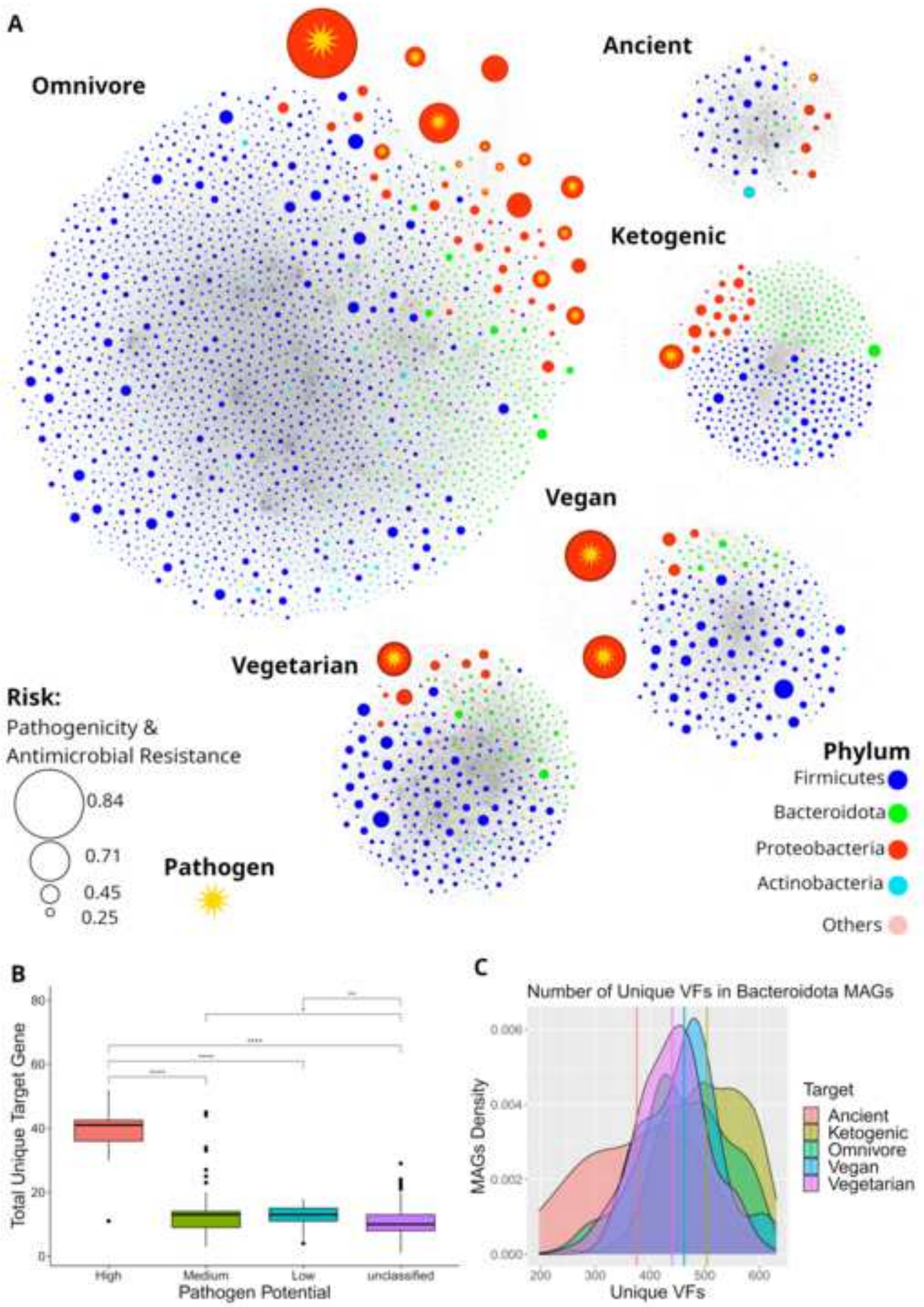

Additional File 1 - Table S1

Click here to access/download
Supplementary Material
01_Kasmanas_gSpread_AddFile1_Table_S1.xlsx

Additional File 2 - Table S2

Click here to access/download
Supplementary Material
02_Kasmanas_gSpread_AddFile2_Table_S2.xlsx

Additional File 3 - Table S3

Click here to access/download

Supplementary Material

03_Kasmanas_gSpread_AddFile3_Table_S3.xlsx

Additional File 4 - Figure S1

Click here to access/download
Supplementary Material
04_Kasmanas_gSpread_AddFile4_Fig_S1.pdf

Additional File 5 - Table S4

Click here to access/download
Supplementary Material
05_Kasmanas_gSpread_AddFile5_Table_S4.xlsx

Additional File 6- Figure S2

Click here to access/download
Supplementary Material
06_Kasmanas_gSpread_AddFile6_Fig_S2.pdf

Additional File 7- Table S5

Click here to access/download
Supplementary Material
07_Kasmanas_gSpread_AddFile7_Table_S5.xlsx

Additional File 8- Figure S3

Click here to access/download
Supplementary Material
08_Kasmanas_gSpread_AddFile8_Fig_S3.pdf

Additional File 9- Table S6

Click here to access/download
Supplementary Material
09_Kasmanas_gSpread_AddFile9_Table_S6.xlsx

Response letter

Click here to access/download

Supplementary Material

00_Kasmanas_gSpread_Cover_Letter_ReviewReply__final.pdf
