# Supplementary material for: Integrating comparative genomics and risk classification by assessing virulence, antimicrobial resistance, and plasmid spread in microbial communities with gSpreadComp
Source: Gigascience. 2025 Jun 26;14:giaf072. doi: 10.1093/gigascience/giaf072 (PMC12199706; doi:10.1093/gigascience/giaf072)
Supplement: giaf072_Authors_Response_To_Reviewer_Comments_original_submission [file giaf072_authors_response_to_reviewer_comments_original_submission.pdf]

Author's Response To Reviewer Comments

POINT-BY-POINT REPLY TO REVIEWERS

GIGA-D-24-00045
gSpreadComp: a flexible pipeline for prokaryotic quantitative risk assessment, gene spread, and plasmid-mediated horizontal transmission to pathogens in microbial communities
Jonas Kasmanas; Stefanía Magnúsdóttir; Junya Zhang; Kornelia Smalla; Michael Schloter; Peter F. Stadler; André Carlos Ponce de Leon Ferreira de Carvalho; Ulisses Rocha
GigaScience

Editor (main comment)

Comment 1
The reviewer's report is below. One very important point is that the reviewer was not able to access the github repository - please check this to make sure the tool and documentation are accessible.

Reply 1:
In the previous manuscript version, we submitted a link using the gitfront interface to share the repository privately with the reviewers before making it public. We have moved the repository to its permanent public address under the Microbial Data Science (MDS) GitHub account:
https://github.com/mdsufz/gSpreadComp/.

Reviewer Report:

Reviewer #1:

Comment 2:
The manuscript by titled " Streamlining microbial community analysis for potential resistance, virulence, and plasmid-mediated spread through integrated comparative genomics and relative risk ranking using gSpreadComp" by Kasmanas et al. introduces gSpreadComp, a UNIX-based workflow that aims to simplify comparative genomics by integrating multiple analytical steps—taxonomic assignment, genome quality checks, AMR and VF gene annotation, plasmid classification, and risk ranking—into a single, coherent pipeline. The manuscript's well-structured methodology and extensive supplementary materials reflect a high standard of technical rigor, while the use case involving human gut microbiomes under diverse dietary conditions, including ancient samples, effectively illustrates the workflow's ability to identify potential resistance and virulence hotspots. The manuscript is clearly written, logically organised, and provides sufficient methodological detail to inspire confidence in the tool's potential for guiding more targeted laboratory investigations and advancing comparative genomics research. However, difficulties in accessing the repository currently limit the ability to test the tool directly.

Reply 2:
We thank the reviewer for the relevant observation and appreciate the recognition of our work. We have corrected the repository availability issue. The tool is now publicly accessible at
https://github.com/mdsufz/gSpreadComp/. Previously, the repository was private and shared through the gitfront interface, which probably caused this technical issue. The repository is now publicly available on GitHub under the Microbial Data Science account.
Major Comments:

Comment 3
The manuscript references a detailed online manual (which is comprehensive and easy to follow) and multiple supplementary files. While this documentation is commendable, I was unable to access the GitHub repository:
o git clone https://github.com/JotaKas/gSpreadComp.git
Cloning into 'gSpreadComp'...

remote: Repository not found.
fatal: repository 'https://github.com/JotaKas/gSpreadComp.git/' not found
The authors must ensure that the repository URL is correct and public, or provide an updated, accessible link. Without this I cannot test if the tool works as described and that raises immediate reproducibility concerns.

Reply 3:
We thank the reviewer for the comment. We made the repository publicly available on the Microbial Data Science GitHub page: https://github.com/mdsufz/gSpreadComp/

Comment 4
Lines 460-493 (Tool Comparison): I suggest adding a table that highlights the advantages gSpreadComp offers compared to PathoFact and MetaCHIP (e.g., inputs, analysis types, key outputs)

Reply 4:
We thank the reviewer for the suggestion. We created Table 3 (Line 468) and included interpretability to the information suggested by the reviewer.

Comment 5
Line 231-237: given that ancient samples are directly compared, adding a sentence on how sample uncertainties influence the interpretation of ARGs and HGT results would be helpful.

Reply 5:
We thank the reviewer for the suggestion. We have addressed this concern by including more detail in lines 234-236 about how the unique challenges of ancient DNA samples can influence the interpretation of our results.

Minor comments:

Comment 6
Lines 524-526: The manuscript mentions conda environments, but the authors should consider also noting if containerised distributions (e.g., Docker/Singularity) are planned or available.

Reply 6:
We thank the reviewer for the suggestion. We plan to develop a Singularity container for gSpreadComp to facilitate further deployment across different computing environments. We added this information to the updated manuscript (lines 528-529).

Comment 7
Line 627-628: The authors mention removing samples with fewer than six genome representatives before calculating gene prevalence. Please provide a brief rationale as to why this cutoff was chosen.

Reply 7:
We thank the reviewer for the comment. We selected the threshold of six genome representatives to reduce statistical bias in the prevalence analyses. A lower number of recovered genomes typically indicates insufficient sequencing depth in a sample, which can skew the prevalence analyses and can result in unreliable prevalence estimates. We have clarified this rationale in the revised manuscript (lines 630-632).

Comment 8
How does the tool handle error? Does it provide any diagnostic logs (i.e., which steps passed/failed?)

Reply 8:
We thank the reviewer for the question. gSpreadComp is designed with a modular architecture in which each operation is executed sequentially with provenance tracking. This design allows users to quickly identify at which step an error occurred. Therefore, if an error occurs due to wrong formatting, the workflow breaks and displays built-in error messages targeted to a specific workflow step, facilitating troubleshooting.

For modules that incorporate third-party software (e.g., GTDBtk for taxonomy assignment), error management is handled by the respective tools. gSpreadComp displays the step in which an error occurred within its workflow and captures the third-party tool-specific exit status and informative messages.

Comment 9
The current title is rather lengthy, suggest shortening:
Suggestion: "Integrating comparative genomics and risk classification to assess microbial resistance, virulence, and plasmid spread with gSpreadComp"

Reply 9:
We thank the reviewer for the suggestion. Incorporating most of the suggestions made by the reviewer, we decided to change the title to:
"Integrating comparative genomics and risk classification by assessing virulence, antimicrobial resistance, and plasmid spread in microbial communities with gSpreadComp"
This new title indicates the integration of comparative genomics and risk classification. We decided to use "by assessing" to help the reader realize quickly what aspects the integration will resolve. We switched the order of "virulence" and "antimicrobial resistance" to avoid misunderstandings by the reader (e.g., "antimicrobial resistance, virulence…" could indicate that we assessed "antimicrobial virulence"). We also added "in microbial communities" because it may help the reader identify that gSpreadComp deals with complex analyses that may arise from antimicrobial resistance spread in a complex community.

em Editorial Manager®

Cookie Preference Center

We use cookies which are necessary to make our site work. We may also use additional cookies to analyze, improve and personalize our content and your digital experience. For more information, see our Cookie Policy.

You may choose not to allow some types of cookies. However, blocking some types may impact your experience of our site and the services we are able to offer. See the different category headings below to find out more or change your settings.

Allow all

Manage Consent Preferences

Strictly Necessary Cookies

Always active

These cookies are necessary for the website to function and cannot be switched off in our systems. They are usually only set in response to actions made by you which amount to a request for services, such as setting your privacy preferences, logging in or filling in forms. You can set your browser to block or alert you about these cookies, but some parts of the site will not then work. These cookies do not store any personally identifiable information.

Functional Cookies

These cookies enable the website to provide enhanced functionality and personalisation. They may be set by us or by third party providers whose services we have added to our pages. If you do not allow these cookies then some or all of these services may not function properly.

Performance Cookies

These cookies allow us to count visits and traffic sources so we can measure and improve the performance of our site. They help us to know which pages are the most and least popular and see how visitors move around the site.

Targeting Cookies

These cookies may be set through our site by our advertising partners. They may be used by those companies to build a profile of your interests and show you relevant adverts on other sites. If you do not allow these cookies, you will experience less targeted advertising.

Cookie List

Clear

Apply Cancel

Consent Leg.Interest

Confirm my choices

Powered by onetrust
