# Supplementary material for: Integrating comparative genomics and risk classification by assessing virulence, antimicrobial resistance, and plasmid spread in microbial communities with gSpreadComp
Source: Gigascience. 2025 Jun 26;14:giaf072. doi: 10.1093/gigascience/giaf072 (PMC12199706; doi:10.1093/gigascience/giaf072)
Supplement: giaf072_Reviewer_1_Report_Original_Submission — Veronica Jarocki -- 12/9/2024 [file giaf072_reviewer_1_report_original_submission.pdf]

**Reviewer Report**

**Title: Integrating comparative genomics and risk classification by assessing virulence, antimicrobial resistance, and plasmid spread in microbial communities with gSpreadComp**

**Version: Original Submission** **Date:** 12/9/2024

**Reviewer name: Veronica Jarocki**

**Reviewer Comments to Author:**

The manuscript by titled " Streamlining microbial community analysis for potential resistance, virulence, and plasmid-mediated spread through integrated comparative genomics and relative risk ranking using gSpreadComp" by Kasmanas et al. introduces gSpreadComp, a UNIX-based workflow that aims to simplify comparative genomics by integrating multiple analytical stepsâ€”taxonomic assignment, genome quality checks, AMR and VF gene annotation, plasmid classification, and risk rankingâ€”into a single, coherent pipeline. The manuscript's well-structured methodology and extensive supplementary materials reflect a high standard of technical rigor, while the use case involving human gut microbiomes under diverse dietary conditions, including ancient samples, effectively illustrates the workflow's ability to identify potential resistance and virulence hotspots. The manuscript is clearly written, logically organised, and provides sufficient methodological detail to inspire confidence in the tool's potential for guiding more targeted laboratory investigations and advancing comparative genomics research. However, difficulties in accessing the repository currently limit the ability to test the tool directly.Major comments:* The manuscript references a detailed online manual (which is comprehensive and easy to follow) and multiple supplementary files. While this documentation is commendable, I was unable to access the GitHub repository:o git clone https://github.com/JotaKas/gSpreadComp.gitCloning into 'gSpreadComp'...remote: Repository not found.fatal: repository 'https://github.com/JotaKas/gSpreadComp.git/' not foundThe authors must ensure that the repository URL is correct and public, or provide an updated, accessible link. Without this I cannot test if the tool works as described and that raises immediate reproducibility concerns.* Lines 460-493 (Tool Comparison): I suggest adding a table that highlights the advantages gSpreadComp offers compared to PathoFact and MetaCHIP (e.g., inputs, analysis types, key outputs)* Line 231-237: given that ancient samples are directly compared, adding a sentence on how sample uncertainties influence the interpretation of ARGs and HGT results would be helpful.Minor comments:* Lines 524-526: The manuscript mentions conda environments, but the authors should consider also noting if containerised distributions (e.g., Docker/Singularity) are planned or available.* Line 627-628: The authors mention removing samples with fewer than six genome representatives before calculating gene prevalence. Please provide a brief rationale as to why this cutoff was chosen.* How does the tool handle error? Does it provide any diagnostic logs (i.e., which steps passed/failed?)* The current title is rather lengthy, suggest shortening:o Suggestion: "Integrating comparative genomics and risk ranking to assess microbial resistance, virulence, and plasmid spread with gSpreadComp"

**Level of Interest**

Please indicate how interesting you found the manuscript: Choose an item.

**Quality of Written English**

Please indicate the quality of language in the manuscript: Choose an item.

**Declaration of Competing Interests**

Please complete a declaration of competing interests, considering the following questions:

- Have you in the past five years received reimbursements, fees, funding, or salary from an organisation that may in any way gain or lose financially from the publication of this manuscript, either now or in the future?
- Do you hold any stocks or shares in an organisation that may in any way gain or lose financially from the publication of this manuscript, either now or in the future?
- Do you hold or are you currently applying for any patents relating to the content of the manuscript?
- Have you received reimbursements, fees, funding, or salary from an organization that holds or has applied for patents relating to the content of the manuscript?
- Do you have any other financial competing interests?
- Do you have any non-financial competing interests in relation to this paper?

If you can answer no to all of the above, write 'I declare that I have no competing interests' below. If your reply is yes to any, please give details below.

I declare that I have no competing interests

I agree to the open peer review policy of the journal. I understand that my name will be included on my report to the authors and, if the manuscript is accepted for publication, my named report including any attachments I upload will be posted on the website along with the authors' responses. I agree for my report to be made available under an Open Access Creative Commons CC-BY license (http://creativecommons.org/licenses/by/4.0/). I understand that any comments which I do not wish to be included in my named report can be included as confidential comments to the editors, which will not be published.

Choose an item.

To further support our reviewers, we have joined with Publons, where you can gain additional credit to further highlight your hard work (see: https://publons.com/journal/530/gigascience). On publication of this paper, your review will be automatically added to Publons, you can then choose whether or not to claim your Publons credit. I understand this statement.

Yes Choose an item.
